# Learning heterogeneous reaction kinetics from X-ray videos pixel by pixel

**DOI:** 10.1038/s41586-023-06393-x

**Published:** 2023-09-13

**Authors:** Hongbo Zhao, Haitao Dean Deng, Alexander E. Cohen, Jongwoo Lim, Yiyang Li, Dimitrios Fraggedakis, Benben Jiang, Brian D. Storey, William C. Chueh, Richard D. Braatz, Martin Z. Bazant

**Affiliations:** 1 Department of Chemical Engineering, Massachusetts Institute of Technology, Cambridge, MA USA; 2 Department of Materials Science and Engineering, Stanford University, Stanford, CA USA; 3 Toyota Research Institute, Cambridge, MA USA; 4 Stanford Institute for Materials and Energy Sciences, SLAC National Accelerator Laboratory, Menlo Park, CA, USA; 5 Department of Mathematics, Massachusetts Institute of Technology, Cambridge, MA USA

**Keywords:** Energy modelling, Applied mathematics, Theory and computation

## Abstract

Reaction rates at spatially heterogeneous, unstable interfaces are notoriously difficult to quantify, yet are essential in engineering many chemical systems, such as batteries^1^ and electrocatalysts^2^. Experimental characterizations of such materials by operando microscopy produce rich image datasets^3–6^, but data-driven methods to learn physics from these images are still lacking because of the complex coupling of reaction kinetics, surface chemistry and phase separation^7^. Here we show that heterogeneous reaction kinetics can be learned from in situ scanning transmission X-ray microscopy (STXM) images of carbon-coated lithium iron phosphate (LFP) nanoparticles. Combining a large dataset of STXM images with a thermodynamically consistent electrochemical phase-field model, partial differential equation (PDE)-constrained optimization and uncertainty quantification, we extract the free-energy landscape and reaction kinetics and verify their consistency with theoretical models. We also simultaneously learn the spatial heterogeneity of the reaction rate, which closely matches the carbon-coating thickness profiles obtained through Auger electron microscopy (AEM). Across 180,000 image pixels, the mean discrepancy with the learned model is remarkably small (<7%) and comparable with experimental noise. Our results open the possibility of learning nonequilibrium material properties beyond the reach of traditional experimental methods and offer a new non-destructive technique for characterizing and optimizing heterogeneous reactive surfaces.

## Abstract (web summary)

Analysis of a large dataset of scanning transmission X-ray microscopy images of carbon-coated lithium iron phosphate nanoparticles shows that the heterogeneous reaction kinetics of battery materials can be learned from such videos pixel by pixel.

## Main

Many processes in nature are controlled by defects, from the atomic scale to the macroscale^8^. As such, quantifying and explaining heterogeneities is a critical task in all branches of science and engineering. Most heterogeneities are nontrivial because they compound the effects of thermodynamics and kinetics and are path dependent. This is especially true in dynamically evolving systems, such as batteries and catalysts^1^. The state-of-the-art approach uses operando microscopy (that is, optical, electron and X-ray) to record dynamically evolving patterns^3–6,9–11^. Ideally, measuring many dynamic properties at once can explain heterogeneities, such as bulk and surface chemistry, as well as reaction kinetics and local chemistry. However, this is experimentally challenging owing to the orthogonal requirements of measurement methods. An alternative approach recognizes that such videos, even of just a single property, harbour a huge amount of information and uses image-learning methods to explain heterogeneities and reveal hidden physics^12,13^.

One of the most challenging heterogeneities to explain is that of reaction kinetics at interfaces. For example, in intercalation materials for batteries and electrocatalysts^1,2^, pattern formation at the solid–liquid interface is a convolution of bulk phase separation thermodynamics, nanoscale variation in surface/coating chemistry and nonlinear reaction kinetics^7,14^. At present, there is no single characterization technique that can decouple and reveal all three aspects directly at the same time. This complex interplay of heterogeneity impedes the assimilation of full-image data into the development of new physical models. Such is the case in LFP^15^, a widely used Li-ion battery positive electrode material. Decades of theoretical and experimental research at various length scales have produced valuable insight into the mechanisms behind the favourable electrochemical performance of LFP^16–18^. However, so far, no theoretical models have been directly validated on a pixel-by-pixel basis.

**Fig. 1:**
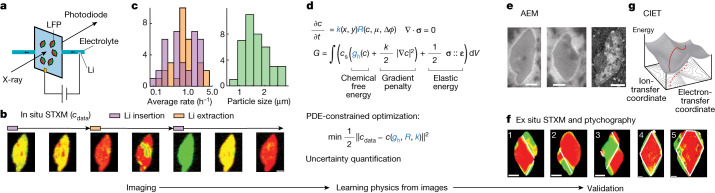
Workflow of learning the physics of battery nanoparticles from images. **a**,**b**, Using in situ STXM, spatiotemporal mapping of lithium concentration in LFP nanoparticles averaged in the [010] (*b*) direction during battery cycling is obtained for 39 particles. Images show the (010) (*a*–*c*) plane. Each particle may have undergone several charge and discharge half-cycles. **c**, Histograms of the particle size (major axis length) and average charge and discharge rates of all imaged particles, defined to be the average rate of change in the Li fraction from the first to the last image of each half-cycle. **d**, In situ STXM images are used as training data *c*_data_ in the optimization, which minimizes the squared sum of the errors at all the pixels subject to the constraint of the model, with respect to the unknowns—homogeneous free energy *g*_h_(*c*), reaction rate *R* and spatial heterogeneity *k*(*x*, *y*)—highlighted in blue. Cross-validation and uncertainty quantification are performed subsequently. **e**, The results are further validated using AEM, which shows the distribution of carbon on the particle surfaces compared with the heterogeneity *k*(*x*, *y*). **f**, Comparison of the morphology of the phase-separated domains from ex situ STXM (particles 1–3) and ptychography images (particles 4 and 5) of fully relaxed particles with model-predicted interfaces indicated by the white lines (defined to be the contour curve of *c*(*x*, *y*) = 0.5), which validates the mechanical model. **g**, Comparison of the reaction kinetics with CIET theory. The red curve is a sketch of the reaction pathway over the energy landscape as electron transfer from carbon to the Fe^3+/2+^ redox sites and Li^+^ ion transfer from carbon to LFP occur (adapted with permission from ref. ^51^). Scale bars, 500 nm.

In this work, we develop a framework that consists of imaging, modelling and data analytics to explain the behaviour of LFP nanoparticles and demonstrate the inverse learning of physics from X-ray videos on a pixel-by-pixel basis (Fig. 1). By recognizing that such videos encode all of the information of interest, our framework successfully separates each contribution to heterogeneity: phase separation, coating and nonlinear reaction kinetics, which are then validated by theoretical models and alternative measurements. Our framework of learning hidden physics and explaining complex heterogeneities from videos is generalizable and readily applicable to new systems. Our approach elevates the multiscale insights that can be extracted from state-of-the-art microscopy.

## Experimental images

In our study, LFP platelet particles in a microfluidic electrochemical cell are imaged using STXM. LFP and lithium metal are the two electrodes of the cell (Fig. 1a). During discharge, lithium ions intercalate into the LFP crystal lattice from the electrolyte (Li^+^ + FePO_4_ + *e*^−^ → LiFePO_4_), whereas during charge, the reaction moves in the opposite direction. Images of local X-ray absorption are converted to the Li concentration field depth-averaged in the [010] (*b*) direction, revealing the dynamics of lithium insertion and extraction (Fig. 1b).

At low reaction rates or at equilibrium, the lithium in a single particle is not uniformly distributed. Instead, heterogeneity arises from phase-separated regions that are either lithium poor (Li_*δ*_FePO_4_) or rich (Li_1−*δ*_FePO_4_), for which *δ* is around 0.035 at room temperature^19–22^ (Fig. 1f). Phase separation is suppressed at high reaction rates and during lithium insertion, which results in a more uniform lithium concentration field^1^ (Fig. 1b). Images of 39 particles are obtained, each of which undergoes one or more half-cycles of charge or discharge (Fig. 1c). In total, our data contain 62 half-cycles, each consisting of 4–10 images in time (Supplementary Information section 1.3). The cell is cycled at rates that correspond to charging all LFP particles in the cell to the full theoretical capacity in 20 min to 5 h (3.0 C to 0.2 C). The particles are found to be on average 1.2 μm^2^ in size, which corresponds to 490 pixels for a total dataset size of 1.8 × 10^5^ pixels.

## Theoretical framework

Within the thin LFP platelet particles (about 150 nm in the *b* direction), lithium concentration is typically modelled using a depth-averaged, reaction-limited Allen–Cahn reaction model^7,19,20,23,24^, which captures the evolution of the depth-averaged Li fraction *c* in the (010) (*a*–*c*) plane (Fig. 1b):

1 $$\frac{\partial c}{\partial t}=k(x,y)R(c,\eta ),$$

in which *k*(*x*, *y*) is a prefactor that describes the heterogeneity on the imaged 2D plane (*x*, *y*), *R* is the reaction rate for a spatially uniform system and *η* is the overpotential.

The total free energy of the LFP particle, *G*, consists of the homogeneous chemical free-energy density, *g*_h_(*c*), to be learned from images, the gradient penalty (modelling nonlocal effects in mean-field theory) and the elastic coherency strain energy^7,20,25^ (Fig. 1d). The constitutive law for the mechanical model is obtained from another study based on correlative images of lithium fraction and strain and is explained in Supplementary Information section 2.2 (ref. ^26^). The (diffusional) chemical potential of Li in LFP is defined as the variational derivative of free energy $\mu \equiv {c}_{{\rm{s}}}^{-1}\delta G[c]/\delta c$, in which *c*_s_ is the lattice site density.

We impose a minimal assumption about the functional form of the reaction rate, *R*, that it follows Butler–Volmer (BV) kinetics^27^, or $R={j}_{0}(c)\left({e}^{-\alpha \widetilde{\eta }}-{e}^{\left(1-\alpha \right)\widetilde{\eta }}\right)$, in which *j*_0_(*c*) is the exchange current (normalized by the Faraday constant), to be learned from the images, *α* is the symmetry factor (*α* = 0.5 (ref. ^28^)), $\widetilde{\eta }\equiv \,(\mu -{\mu }_{{{\rm{Li}}}^{+}}+e\Delta \phi )\,/\,({k}_{{\rm{B}}}T)\,\equiv e\eta /({k}_{{\rm{B}}}T)$ is the normalized overpotential (the local driving force for the reaction), ${\mu }_{{{\rm{Li}}}^{+}}$ is the chemical potential of Li^+^ in the electrolyte and Δ*ϕ* is the interfacial voltage. Recently, the theory of coupled ion-electron transfer (CIET), which considers the simultaneous transfer of ion and electron and the associated energy landscape, has been shown to predict the electrochemical reaction kinetics of non-phase-separating materials, such as lithium transition metal oxides, well with very few parameters but has not been fully validated for heterogeneous phase-separating materials, such as the LFP nanoparticles considered here^29,30^ (Fig. 1g). In the low-overpotential regime, CIET and BV kinetics predict similar overpotential dependence (see Supplementary Information section 9.5 for the distribution of overpotential, which has a standard deviation of 2.7*k*_B_*T*/*e*). We adopt BV kinetics, which has a separable dependence on *c* and *η*, infer the unknown *j*_0_(*c*) from image data and later compare with the exchange current *j*_0_(*c*) predicted by the CIET theory. See Supplementary Information section 2.1 for the full set of governing equations.

The model above assumes that particles have spatially uniform properties. However, previous work has suggested that certain domains react faster than others during both charge and discharge^1^. We propose that these kinetic hotspots can be modelled by a spatially varying, dimensionless multiplicative prefactor *k*(*x*, *y*) for the local reaction rate *R*, for which *k*(*x*, *y*) is unique for each particle and remains identical for all charge and discharge cycles of the same particle. With few exceptions, the spatial heterogeneity estimated on the basis of either charge or discharge data of the same particle show good agreement (Supplementary Information section 4.2), justifying *k*(*x*, *y*) as being a multiplicative prefactor. Later, we show that this spatial prefactor is not arbitrary but strongly correlated with the existence of non-uniform carbon coatings on the particles, which serve as the electron conductor.

Combining the reaction kinetics *j*_0_(*c*), thermodynamic free energy *g*_h_(*c*) and spatial heterogeneity *k*(*x*, *y*), the model is a generic formulation of reaction-limited phase separation for a nanoparticle with a heterogeneous surface that may arise from particle coatings or other experimental heterogeneities.

## Full-image inverse problem

On the basis of the model, we infer the unknown constitutive laws and the spatial heterogeneity from all pixels in the images using PDE-constrained optimization^31,32^. The unknown *g*_h_(*c*) and *j*_0_(*c*) are parameterized by a parameter vector **p**_global_ shared by all particles. Spatial heterogeneity of the individual particle *i* is parameterized by **Z**_*i*_. These parameters represent coefficients of orthogonal basis functions that describe *g*_h_(*c*), ln*j*_0_(*c*) and ln*k*(*x*, *y*) (Supplementary Information sections 2.3 and 4.1).

To find the maximum a posteriori (MAP) estimate, we formulate an optimization problem based on Bayesian inference. The likelihood assumes that the experimental error at each pixel follows an independent and identically distributed normal distribution (Supplementary Information section 5.2), whereas a Gaussian prior is used for the parameters (Supplementary Information section 2.3). The objective function to be minimized is given by:

2 $$\mathop{\min }\limits_{{{\bf{p}}}_{{\rm{global}}},{{\bf{Z}}}_{i}}\left[\sum _{i}\sum _{j}\parallel {c}_{i,j}({{\bf{p}}}_{{\rm{global}}},{{\bf{Z}}}_{i})-{c}_{{\rm{data}},i,j}{\parallel }^{2}+{\rho }_{1}\parallel {{\bf{p}}}_{{\rm{global}}}{\parallel }^{2}+{\rho }_{2}\sum _{i}\parallel {{\bf{Z}}}_{i}{\parallel }^{2}\right],$$

in which *c*_*i*,*j*_ and *c*_data,*i*,*j*_ are the model prediction and experimental concentration field of the *j*th half-cycle of particle *i*, respectively, the norm of their difference is defined to be the sum of squared errors of all pixels inside the particles (Supplementary Information section 6.1) and *ρ*_1_ and *ρ*_2_ are regularization parameters. The minimization is subject to the constraint of equation (1) and the BV reaction kinetics. Further details on the optimization algorithm, parameter identifiability and the regularization parameters can be found in Methods.

**Fig. 2:**
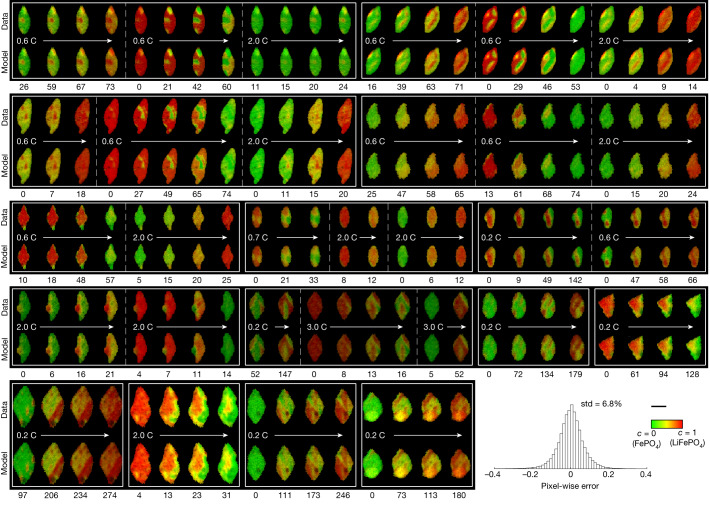
Experimental and simulated Li concentration maps for key frames of selected particles (see Supplementary Fig. 57 for the full list of particles, cycles and frames and Supplementary Video 1 for the full comparison interpolated over time). The rows labelled ‘Data’ and ‘Model’ are experimental images and the prediction of the model trained on the entire dataset at the optimal regularization coefficient *ρ*_2_ = 0.88, respectively. The number below each frame is the time in minutes elapsed since the initial frame of the half-cycle. The inferred model shows close agreement with the experimental data. The C rate (defined as the time in hours to fully charge or discharge to the theoretical capacity) of the cell is labelled below each half-cycle. Different half-cycles are separated by dashed lines. At the bottom right is the histogram of training error (the difference between data and model, *c*_data_ − *c*) of all 1.5 × 10^5^ pixels in the dataset (excluding the initial frames), including those not shown, and the colour bar and the scale bar are shared by all the images in this figure. Scale bar, 1 μm.

Next, we perform *k*-fold cross-validation (Supplementary Information section 8.5) on particles with two and three half-cycles (Supplementary Table 1) to find the optimal regularization parameter. The model is trained on *k* − 1 half-cycles and validated on the remaining one. Using root mean squared error (RMSE) at all pixels to evaluate the errors, we find that the minimum validation error with threefold cross-validation is 9.6 ± 0.9% (Supplementary Information section 8.6 and Supplementary Fig. 41) and the training error at the corresponding optimal regularization parameter is 6.8%, close to the experimental error of 7% (Supplementary Information section 1.2). Figure 2 presents a comparison between the data and model trained at the optimal regularization parameter for a selected number of frames and particles and the histogram of pixel-wise error. See Supplementary Fig. 57 for the inversion results of all particles and their inferred spatial heterogeneities.

**Fig. 3:**
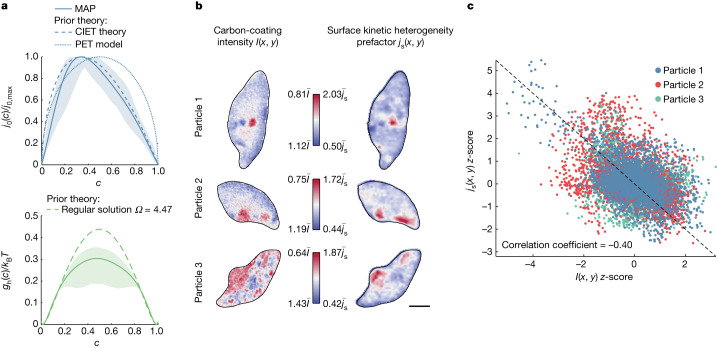
Validation of the learned physics. **a**, Comparison of the constitutive laws of reaction kinetics and thermodynamics obtained from image inversion with theoretical models. Solid lines are MAP results of exchange current *j*_0_(*c*)/*j*_0,max_ and homogeneous free energy *g*_h_(*c*) that correspond to Fig. 2. Shaded regions are their 99% confidence interval obtained from bootstrapping. The learned exchange current (top) is compared to CIET theory (equation (3))^29^ for electron-coupled ion transfer^34^ as used in multiphase porous electrode theory (MPET) (ref. ^36^) (dashed curve), as well as the empirical form ${j}_{0}(c)\propto \sqrt{c(1-c)}$ used to model lithium intercalation with Butler-Volmer kinetics^39^ in classical PET (ref. ^27^) (dotted curve). The learned free energy (bottom) is compared to the regular solution model (Ω = 4.47, dashed curve)^20,39^. **b**, Comparison of the AEM carbon signal *I*(*x*, *y*) and inferred spatial variation of surface reaction rate *j*_s_(*x*, *y*). The colour maps for *I*(*x*, *y*) and *j*_s_(*x*, *y*) range from the minimum to the maximum values scaled by the spatial mean $\bar{I}$ and ${\bar{j}}_{{\rm{s}}}$ for each particle, respectively. *j*_s_(*x*, *y*) = *k*(*x*, *y*)*h*(*x*, *y*), in which *k*(*x*, *y*) is obtained from inversion using the optimal regularization parameter and *h*(*x*, *y*) is the optical density normalized by the mean, which is proportional to the particle thickness. Scale bar, 500 nm. **c**, Pixel-wise comparison of rescaled *j*_s_(*x*, *y*) and AEM image intensity *I*(*x*, *y*) for the three particles.

We then estimate the uncertainty of the inferred quantities using bootstrapping^33^, which repeats the optimization by randomly sampling the 39 particles with replacement (see Supplementary Information section 9.1 for details and uncertainty quantification using Hamiltonian Monte Carlo (HMC)). In Fig. 3a, the shaded region represents the 99% confidence region of *g*_h_(*c*) and *j*_0_(*c*) obtained through bootstrapping. The solid curves correspond to the MAP estimation in Fig. 2. Notably, the bootstrapping samples consistently indicate an asymmetric shape for the normalized exchange current *j*_0_(*c*) (see Supplementary Fig. 46).

## Validation of the learned physics

### Reaction kinetics

The inferred normalized exchange current agrees remarkably well with the prediction of CIET theory^29,34^ (dashed line in Fig. 3a), which can be approximated by the simple formula^35,36^ (see Supplementary Information section 9.5),

3 $${j}_{0,{\rm{CIET}}}(c)={j}_{{\rm{r}}}^{* }\left(1-c\right)\frac{{\widetilde{c}}_{+}c}{{\widetilde{c}}_{+}+c}{\rm{erfc}}\left(\frac{\widetilde{\lambda }-\sqrt{1+\sqrt{\widetilde{\lambda }}+{{\rm{ln}}}^{2}\left(\frac{{\widetilde{c}}_{+}}{c}\right)}}{2\sqrt{\widetilde{\lambda }}}\right)$$

in the case of rate-limiting electron transfer (or ‘electron-coupled ion transfer’, ECIT (ref. ^34^)) from the metallic carbon coating to the Fe^3+/2+^ redox site^28^ coupled with Li^+^ ion transfer from the coating into the crystal lattice, excluding one site^7^, in which ${\widetilde{c}}_{+}$ is the fractional coverage of reactive surface sites by absorbed Li^+^, $\widetilde{\lambda }$ is the Marcus reorganization energy normalized by *k*_B_*T* (refs. ^37,38^) and ${j}_{{\rm{r}}}^{* }$ is a constant that depends on the ion-transfer energy, electronic coupling and temperature^29^. CIET theory closely matches the inferred normalized exchange current without any adjustable parameters (Fig. 3a), using the literature value $\widetilde{\lambda }=8.3$ for LFP^28^ and a strong surface adsorption ${\widetilde{c}}_{+}=1$ (see Supplementary Information section 9.5 for comparison of CIET theory and inferred reaction kinetics versus overpotential $\widetilde{\eta }$ and ${\widetilde{c}}_{+}$). The successful validation of the concentration dependence of the CIET reaction rate could not be accomplished without resolving spatial heterogeneity by image inversion.

To validate that the inverted exchange current captures the key qualitative features of the images—the uniformity of the Li concentration field and that the result was not compounded by the spatial heterogeneity—we remove the spatial information and train the model using only a metric called the ‘uniformity coefficient’ based on the variance of *c*(*x*, *t*) in space^1^, instead of the full images. HMC sampling confirms that the inferred exchange current remains asymmetric, albeit with higher uncertainty compared with using full images (Supplementary Fig. 14).

The consistency of the inferred *j*_0_(*c*) indicates its important role in controlling the spatial pattern. When the autocatalytic rate of the reaction *s* ≡ ∂*R*/∂*c* is negative, linear stability analysis shows that phase separation is suppressed, an effect known as electroautocatalysis^14^. When d*j*_0_/d*c* < 0, *j*_0_(*c*) has an autoinhibitory effect during insertion (*R* > 0) and an autocatalytic effect during extraction (*R* < 0), and the opposite effect when d*j*_0_/d*c* > 0. Our result in Fig. 3a shows that Li intercalation in LFP has a *j*_0_(*c*) that is skewed towards lower *c*; as a result, Li concentration is more uniform during lithium insertion than extraction^1,19^, as shown consistently by both experiments and simulations (Supplementary Figs. 12 and 55). We also find that, given only the uniformity coefficient, *j*_0_(*c*) and *g*_h_(*c*) lie on a manifold that is defined by the contours of the normalized autocatalytic rate *s*/*R* (Supplementary Information section 5.1 and Supplementary Fig. 13).

In comparison, the exchange current model, ${j}_{0}(c)\propto \sqrt{c\left(1-c\right)}$, commonly assumed in porous electrode theory^39^ (dotted line in Fig. 3a), cannot account for the asymmetry and is inconsistent with the inferred asymmetric exchange current and the general predictions of CIET theory. Furthermore, the ECIT limit contains no fitting parameters besides the reorganization energy, which was obtained from separate measurements. Hence, the close agreement with the inferred reaction kinetics provides stronger evidence for the validity of the theory.

### Nonequilibrium thermodynamics

The inferred free-energy landscape *g*_h_(*c*) in Fig. 3a is consistent with the standard regular solution model for LFP^7,20^, ${g}_{{\rm{h}}}(c)/{k}_{{\rm{B}}}T\,=\,c\,{\rm{ln}}\,c\,+$ $\left(1-c\right){\rm{ln}}\left(1-c\right)+\varOmega c\left(1-c\right)$ (dashed line, *Ω* = 4.47), although it exhibits a smaller nucleation barrier (maximum between the binodal points). We warn that the inferred *g*_h_(*c*) can depend on the choice of the model and *κ*, which—in this study—is chosen to be the literature value owing to its non-identifiability, as explained in Methods.

### Chemomechanics

The insertion of Li causes anisotropic deformation of the LFP lattice^26,40,41^. As a result, the minimum energy state of intraparticle phase separation corresponds to an interface with a preferred orientation that is determined by the misfit strain, stiffness tensor and interfacial energy^20,42^.

Given the geometry of the experimentally observed particle and its average Li fraction, we numerically find the minimum energy state using our model through relaxation from either the experimental image or a uniform concentration field (Supplementary Information section 3). Figure 1f shows the simulated interface between Li-rich and Li-poor phases indicated by the white lines. The consistency between STXM and ptychography images^26^ of relaxed particles and simulations (taking into account the reflection symmetry) validates the chemomechanical model.

### Surface heterogeneity

The inclusion of *k*(*x*, *y*) in the model accounts for the observation of fast and slow regions. We further validate this modelling choice by full-image inversion, which shows that *k*(*x*, *y*) trained on different half-cycles of the same particle shows good agreement (Supplementary Information section 9.2 and Supplementary Figs. 47 and 48). We found that, at the optimal regularization parameter *ρ*_2_, the square root of the interparticle, intraparticle and overall variance of ln*k*(*x*, *y*) in space are 0.58, 0.21 and 0.62, respectively (see equation (54) for definition). The quantification of the variability can be useful in full-electrode simulations to identify modes of failure owing to kinetic hotspots^36^.

To understand the origin of the heterogeneity, we first remove the effect of non-uniform particle thickness *h*(*x*, *y*), which is proportional to the STXM optical density map, and obtain the effect of surface heterogeneity based on the depth-averaged model, *k*(*x*, *y*) = *j*_s_(*x*, *y*)/*h*(*x*, *y*), in which *j*_s_(*x*, *y*) accounts for spatial variation in the surface-reaction rate. Next, *j*_s_(*x*, *y*) is compared with the AEM intensity *I*(*x*, *y*) of surface carbon (Fig. 3b), for which higher intensity indicates thicker carbon coating, whose spatial variation results from non-uniform coating of the carbon precursor (sucrose) on LFP particles owing to particle-to-particle contact and local gas flow during the coating process. Remarkably, the two quantities are closely correlated in space (Fig. 3c), with a pixel-to-pixel correlation coefficient of −0.4 (Supplementary Information section 9.3). The kinetically fast domains are linked to low AEM intensity, suggesting that a thicker carbon coating impedes the reaction, which is consistent with the reduced rate capability and increased charge-transfer resistance previously observed in electrodes with thicker carbon coating and is attributed to slower Li^+^ transport^43–45^. Because LFP is an insulator with much lower intercalation rate in the absence of carbon coating^46^, this result implies the existence of an optimal carbon-coating thickness below the observed minimum value for these particles.

## Conclusion

We have demonstrated the possibility of learning nanoscale physics of a heterogeneous, chemically reactive, phase-separating material by inverting images of its dynamics far from equilibrium. The nonequilibrium thermodynamics, reaction kinetics and surface heterogeneity of LFP nanoparticles used in Li-ion batteries are extracted by direct inversion of in situ STXM images on a pixel-by-pixel basis. Simulated images with the learned model are almost indistinguishable from the experimental images, with 6.8% training error and 9.6 ± 0.9% validation error at optimal regularization.

To our knowledge, this is the first experimental mapping of the inaccessible, unstable region of the free-energy landscape for a pattern-forming system. Our inversion methodology also enables the measurement of the concentration dependence of the reaction rate during phase separation, which provides strong evidence for CIET as the mechanism for lithium intercalation. Inferred spatial heterogeneities in the reaction rate are shown to be correlated with carbon-coating thickness.

These results open new directions for interfacial engineering of batteries and other chemically reactive systems. Images of other crystallographic planes, different electrolytes, surface coatings, charging protocols and measurements of local potential would enable even more precise determination of physics, including lateral or surface diffusion, dynamics in the depth direction and the dependence of the reaction kinetics on the driving force. Leveraging advancements in scientific machine learning and uncertainty quantification^47–50^, the pixel-by-pixel image inversion paves way for the data-driven and physics-informed learning of constitutive laws and alternative methods of non-destructive imaging in fields including energy materials, soft matter and biology^12,13^, in which image data are abundant and await to be fully exploited.

## Methods

### Experimental methods

Micron-sized LiFePO_4_ platelets were synthesized using a solvothermal method^1,26^. LiFePO_4_ particles were mixed with sucrose at a ratio of 5:1 and heated to 600 °C for 5 h in a tube furnace under flowing Ar to yield the carbon-coated LiFePO_4_. The STXM experiment was conducted at beamlines 11.0.2.2 and 5.3.2.1 of the Advanced Light Source (ALS). LFP particles were dispersed on the working electrode of the liquid STXM imaging platform. X-ray absorption at Fe(II) and Fe(III) *L*_3_ edge was measured in situ and used to obtain the depth-averaged Li fraction map for each particle.

The experimental uncertainty of Li fraction *c* is estimated by inferring *c* based on the X-ray absorption at several energy levels with bootstrapping, which returns a standard deviation of *c*. The average of the standard deviation over all pixels and particles used in the uncertainty analysis is 0.072. A previous estimate of the standard deviation of Li fraction based on reference images is 0.06 (ref. ^1^). Therefore, we estimate that the standard deviation of the error of *c* is *σ*_*ϵ*_ ≈ 0.07.

More details on the synthesis, structure and electrochemical characterization, STXM and AEM imaging, data processing and analysis of experimental uncertainty can be found in Supplementary Information sections 1.1, 1.2 and 6.2.

### Chemomechanics

The presence of elastic energy in the free-energy functional arises from the lattice deformation occurring as Li intercalates into the LFP crystal. The elastic constants *C*_*ijkl*_ can be found in Supplementary Information section 2.2. The constitutive law for the chemomechanical coupling relates the misfit strain tensor (or the deformation of the crystal lattice when it is stress free) to the local lithium concentration *ε*^0^(*c*). The elastic stress is ${\sigma }_{ij}={C}_{ijkl}({\varepsilon }_{kl}-{\varepsilon }_{kl}^{0}(c))$, in which *ε*_*kl*_ is the total strain.

The misfit strain of LFP is anisotropic—insertion of Li expands the lattice in the *a* direction and contracts in the *c* direction. To minimize the elastic energy, the interface between Li-rich and Li-poor phases at equilibrium tends to be aligned in the [101] direction^20,25^ (Supplementary Information section 2.2). Therefore, it is crucial to determine the principal directions (*a* and *c*) for each imaged particle. By comparing images of relaxed particles and the simulation result based on the same particle geometry, we find that the major axis aligns well with the *c* axis, and the largest deviation between the two among all examples is found to be 10° (Supplementary Information section 3). Hence we align the *c*-axis direction to the major axis in our simulations.

### Parameterization

For the PDE-constrained optimization, we need to parameterize the unknown homogeneous free energy *g*_h_(*c*) and exchange current *j*_0_(*c*). Because the LFP solubility *δ* is smaller than the experimental error *σ*_*ϵ*_ = 0.07, *δ* and the unknown constitutive laws cannot be determined from the images. Instead, we use previous knowledge in the literature to inform the binodal composition and the single-phase region (*c* < *δ* and *c* > 1 − *δ*), that is, we represent *μ*_h_(*c*) = d*g*_h_/d*c* as the sum of the ideal mixing entropy ${\rm{ln}}\frac{c}{1-c}$ and Legendre polynomials while subject to the constraint of the experimentally measured values of the binodal compositions (*c* = *δ* and 1 − *δ*)^31,32^. ln*j*_0_(*c*) is parameterized by Legendre polynomials to ensure that *j*_0_(*c*) is positive. See Supplementary Information sections 2.3 and 6.8 for details of the parameterizations and prior of *μ*_h_(*c*) and *j*_0_(*c*).

Because of the presence of both intraparticle and interparticle heterogeneity, we assume that the spatial heterogeneity $\psi ({\bf{x}})\equiv {\rm{l}}{\rm{n}}k({\bf{x}})$ follows a prior distribution of a Gaussian random field with spatial covariance *C*(**x**_1_ – **x**_2_) with an offset *ψ*_0_ that follows a normal distribution independently, where **x **= (x,y) is the spatial coordinate. We set the correlation length *l* of the Gaussian random field to be the width of a pixel, 50 nm, because spatial variation at the subpixel scale cannot be identified. With this prior distribution, the overall variance of the heterogeneity in space is ${\mathbb{E}}\,[\int {(\psi ({\bf{x}})-{\mathbb{E}}[\bar{\psi }])}^{2}{\rm{d}}{\bf{x}}]\,/\,\int {\rm{d}}{\bf{x}}={\sigma }_{\psi }^{2}+{\sigma }_{{\psi }_{0}}^{2}$, in which we define the mean $\bar{\psi }=\int \psi ({\bf{x}}){\rm{d}}{\bf{x}}/\,\int {\rm{d}}{\bf{x}}$ and the interparticle variation of the mean is ${\mathbb{V}}{\rm{ar}}\left[\bar{\psi }\right]\approx {\sigma }_{{\psi }_{0}}^{2}$ when *l* is much smaller than the size of the particle.

Using Karhunen–Loève expansion, *ψ*(**x**) can be parameterized as

4 $$\psi ({\bf{x}})={\sigma }_{{\psi }_{0}}{Z}_{0}+\mathop{\sum }\limits_{i=1}^{{N}_{{\rm{K}}{\rm{L}}}}\sqrt{{\lambda }_{i}}{\phi }_{i}({\bf{x}}){Z}_{i}$$

in which the basis functions *ϕ*_*i*_(**x**) are eigenfunctions of the covariance function and *λ*_*i*_ is the corresponding eigenvalue,

5 $$\int C({{\bf{x}}}_{1}-{{\bf{x}}}_{2}){\phi }_{i}({{\bf{x}}}_{2}){\rm{d}}{{\bf{x}}}_{2}={\lambda }_{i}{\phi }_{i}({{\bf{x}}}_{1}),$$

and ${Z}_{0},\ldots ,{Z}_{{N}_{{\rm{KL}}}}$ are components of the parameter **Z**_*j*_ used in the optimization for particle *j*. Supplementary Information section 8.4 discusses in detail the regularization of these parameters given *σ*_*ψ*_ and ${\sigma }_{{\psi }_{0}}$.

### PDE-constrained optimization

The optimization is constrained by the PDE model described in the main text and Supplementary Information section 2.1. The coupled PDEs are discretized in space using finite elements (Supplementary Information section 6.3) to obtain a set of differential-algebraic equations (DAEs). The set of DAEs is then solved using numerical differentiation formula with adaptive time stepping and variable order method. See Supplementary Information sections 6.4 and 6.5 for details on the choice of time and spatial accuracy.

The initial condition for *c*(*x*) is set as the initial frame of each half-cycle. For mechanical equilibrium, we enforce a zero-traction boundary condition. Because $\mu ={\mu }_{{\rm{h}}}(c)-\kappa {\nabla }^{2}c-{c}_{{\rm{s}}}^{-1}{\boldsymbol{\sigma }}::{{\boldsymbol{\varepsilon }}}^{0{\prime} }(c)$ (refs. ^7,20,24^), a boundary condition for *c* is needed. The Neumann boundary condition on the normal gradient **n** ⋅ ∇*c*, which relies on knowledge of the surface energy between the solid and the electrolyte, cannot be applied in our case. Hence, to avoid determining the gradient from the image, we impose the Dirichlet boundary condition based on boundary values from the images interpolated in time^31,32^ (Supplementary Information section 6.9 shows that this results in a smaller error compared with the no-wetting boundary condition, in which **n** ⋅ ∇*c* = 0).

The reaction rate *R* is directly determined by the local overpotential *η* and concentration *c*. In simulations, we can specify the voltage Δ*ϕ* as a control variable. However, in experimental settings, it is challenging to precisely measure the local overpotential owing to unknown electric potential losses caused by ohmic and contact resistance, as well as the overpotential loss resulting from concentration polarization. Therefore, we adopt an alternative approach by constraining the total reaction rate of each particle, $I(t)=\int \frac{\partial c}{\partial t}{\rm{d}}V=\frac{{\rm{d}}\bar{c}(t)}{{\rm{d}}t}$, through interpolation of the time-dependent trajectory of the average concentration $\bar{c}(t)$ observed in the experiment. Using this approach, the local potential Δ*ϕ* in the simulation becomes an algebraic variable that satisfies the constraint of the total reaction rate. See Supplementary Information section 6.7 for the analysis of error introduced by the interpolation of $\bar{c}(t)$. We note that the dependence of the rate on the overpotential can only be tested if a precise measurement of the local potential at the particle can be obtained.

We use the trust-region method to solve the PDE-constrained optimization, perform forward sensitivity analysis to compute the gradient of the objective function and use Gauss–Newton approximation for its Hessian^31,32,52^. The optimizer updates **p**_global_ and **Z**_*i*_ (*i* = 1, …, *N*) concurrently. During each iteration, the forward evaluation of the model and its sensitivities for all half-cycles and particles are performed independently and in parallel. By contrast, the adjoint sensitivity analysis computes the gradient of the objective function much faster than forward sensitivity analysis, however, without the approximation for the Hessian, gradient descent converges to a suboptimal solution (Supplementary Information section 6.6).

### Identifiability

We examine the identifiability of the parameters in the model based on a simulated dataset that contains three particles using two approaches. The first approach is HMC sampling of the posterior distribution. At the current level of pixel error *σ*_*ϵ*_ = 0.07, the 99% confidence region of *μ*_h_(*c*) and *j*_0_(*c*) is tightly distributed around the truth, indicating that they can be correctly identified in the neighbourhood of the minimum objective function (Supplementary Fig. 31). A higher error *σ*_*ϵ*_ = 0.3 causes the truth to lie outside the 99% confidence region. The second approach is to run the optimizations starting at different initial guesses. Supplementary Fig. 32 shows that, when the optimizer tolerance is sufficiently low, all of the final RMSE from the optimizer can be below the model solver error. However, if the gradient penalty coefficient *κ* in the chemical potential is unknown and included as a parameter in the optimization, over 80% of the final RMSE is above the solver error (Supplementary Information section 7). Therefore, we use the literature value for *κ* = 5.02 × 10^−10^ J m^−1^ to avoid non-identifiability. See Supplementary Information section 7 for further details.

### Regularization

In our study, the number of parameters for **p**_global_, which represents *g*_h_(*c*) and *j*_0_(*c*), is substantially lower than the number of pixels (Supplementary Information section 5.2). Sensitivity analysis of *g*_h_(*c*) and *j*_0_(*c*) reveals that the variance of the MAP estimate of *g*_h_(*c*) and *j*_0_(*c*) scales inversely with the number of particles, denoted as *N* (Supplementary Information section 8.2 and Supplementary Fig. 34). Also, many particles consist of both charge and discharge cycles, which further decreases the uncertainty of *g*_h_(*c*) and *j*_0_(*c*) (ref. ^32^). We performed regularization on a simulated dataset of three particles with the same level of noise at *σ*_*ϵ*_ = 0.07, which exhibited a negligible reduction in validation error owing to regularization on *g*_h_(*c*) and *j*_0_(*c*) (Supplementary Information section 8.1 and Supplementary Fig. 33). We anticipate an even smaller impact on our dataset with *N* = 39. Therefore, we set *ρ*_1_ = 0 to minimize the bias of **p**_global_ and let **p**_global_ be informed by the available data.

In contrast to **p**_global_, regularization on *k*(*x*, *y*) is necessary, particularly for particles with nearly uniform Li concentration field (Supplementary Information section 8.3). As the rate (*R*/*k*) increases, leading to a more uniform *c*(*x*) profile^7,14,19,53^, the uniformity of *c*(*x*) can provide information about the magnitude of *k*(*x*, *y*). However, as *c*(*x*) becomes nearly uniform, the uncertainty in *k*(*x*, *y*) increases. As a result, the squared error associated with these particles shows insensitivity or lacks local minima with respect to the long-wavelength modes of *ψ*(*x*, *y*), which are constant and smoothly varying in space (Supplementary Information section 8.3). Hence, to constrain the long-wavelength modes, we assume a common prior distribution for *ψ*(*x*, *y*) across all particles. Consequently, the regularization parameter *ρ*_2_ is shared by the **Z**_*i*_ of all particles. Regularization on *k*(*x*, *y*) is also critical to prevent overfitting and leads to a marked reduction in validation error and the error of MAP compared with the truth (Supplementary Information section 8.1 and Supplementary Fig. 33).

We also test using different regularization coefficients for the constant *ψ*_0_ and spatially varying components of *ψ*(*x*, *y*) and find that minimum validation error is reached around when the regularization coefficients are equal (Supplementary Information section 8.4). Because *k*(*x*, *y*) multiplies with *j*_0_(*c*), we impose a normalization constraint on *k*(*x*, *y*), that is, the average of *ψ*(*x*, *y*) over all particles weighted by area is 0 (Supplementary Information section 4.1).

### Cross-validation

We consider two approaches for the cross-validation at different values of *ρ*_2_: (1) allowing **p**_global_ to vary as *ρ*_2_ changes or (2) fixing **p**_global_ at its MAP values when ${\rho }_{2}={\sigma }_{{\epsilon }}^{2}/{\sigma }_{\psi }^{2}=0.01$, in which *σ*_*ϵ*_ ≈ 0.7 was first estimated using finite differences (see Supplementary Information section 4.2). Studies using simulated datasets show that using a small *ρ*_2_ reduces the bias of **p**_global_ (Supplementary Fig. 35). Results on simulated datasets show that the two approaches lead to similar optimal *ρ*_2_ and a comparable reduction in the validation error and MAP error. In the case of experimental datasets, we also perform cross-validation using these two approaches and find that, although the first approach leads to a lower training error, the second approach has a substantially smaller validation error (Supplementary Information section 8.5 and Supplementary Fig. 40). Therefore, we adopt the latter approach, which also allows us to perform *k*-fold cross-validation independently for each particle, as only **Z**_*i*_ is updated while varying *ρ*_2_ (Supplementary Information section 8.6).

We then performed *k*-fold cross-validation, training the model on *k* − 1 half-cycles and validated on the other one. Using the one-standard-error rule^54^, we determine the optimal *ρ*_2_ = 0.88. As mentioned in the main text, the training RMSE at *ρ*_2_ = 0.88 is 6.8%. By contrast, the training RMSE at *ρ*_2_ = 0.01 is 6.0%, suggesting overfitting. Conversely, the training RMSE at *ρ*_2_ → *∞* is 10.6% (Supplementary Table 3 and Supplementary Information section 9.4), that is, the kinetic prefactor is spatially uniform (*k*(*x*, *y*) = 1), which indicates underfitting. At larger *ρ*_2_, the bias in *g*_h_(*c*) and *j*_0_(*c*) is also expected to be large because the bias increases with *ρ*_2_ regardless of the number of particles (Supplementary Information section 8.2 and Supplementary Fig. 35). For a visual comparison of the RMSE using different parameters, refer to Supplementary Fig. 50.

Most of the particles with three half-cycles show a strong correlation among *k*(*x*, *y*) trained on the basis of the three training datasets, and all three validation curves have clear minima around the optimal *ρ*_2_. The presence of a few particles that show a lack of consistency among *k*(*x*, *y*) in cross-validation reflects the possibility of unmodelled effects in certain half-cycles in the heterogeneous dataset (Supplementary Information section 8.6). Therefore, to avoid bias, the reported spatial heterogeneities *k*(*x*, *y*) in Supplementary Fig. 57 are obtained by training on all half-cycles with equal weights using the optimal regularization coefficient.

### Learning from the uniformity coefficient

Using the uniformity coefficient only as the training data and hence discarding spatial information, we use both the full 2D continuum model on a square and a simplified ‘0D’ model that excludes all spatial correlation (spatial heterogeneity is included in both models but only *σ*_*ψ*_ is an unknown parameter, whereas *k*(*x*, *y*) is a fixed and randomly generated Gaussian random field). HMC sampling shows that the posterior distribution of parameters of *j*_0_(*c*) and *μ*_h_(*c*) approximately lies on a manifold that is defined by the contours of the terms in the normalized autocatalytic rate *s*/*R* ((ln*j*_0_)′ and ${j}_{0}{\mu }_{{\rm{h}}}^{{\prime} }$; see Supplementary Fig. 13), resulting in coupling between the magnitude of *j*_0_ and the slope of *μ*_h_(*c*) and between parameters of *j*_0_(*c*). Despite the coupling, the persistent features that arise from the HMC sampling are the non-monotonicity of *μ*_h_(*c*) and the asymmetry of *j*_0_(*c*).

We also include ohmic film resistance, which alters the asymptotic relationship between rate and overpotential at high overpotential, and found that it has a broad posterior distribution over many orders of magnitude and has little correlation with other parameters. Hence, ohmic film resistance is omitted in the full-image inversion.

## Online content

Any methods, additional references, Nature Portfolio reporting summaries, source data, extended data, supplementary information, acknowledgements, peer review information; details of author contributions and competing interests; and statements of data and code availability are available at 10.1038/s41586-023-06393-x.

## Supplementary information

Supplementary Information

Supplementary Video 1 Comparison of experimental and simulated Li concentration maps for all particles and half-cycles. The experimental maps are interpolated in time. The time since the initial frame is indicated by the hour and minute arms.

## Data Availability

STXM and AEM images used in this study and the inversion results of all particles are available at 10.6084/m9.figshare.23682429. Ptychography images are available online at data.matr.io/6.

## Code availability

Codes and methods used in this study are described in refs. ^31,32^.
