# Supplementary material for: Learning heterogeneous reaction kinetics from X-ray videos pixel by pixel
Source: Nature. 2023 Sep 13;621(7978):289–94. doi: 10.1038/s41586-023-06393-x (PMC10499602; doi:10.1038/s41586-023-06393-x)
Supplement: Supplementary file 1 — Supplementary Information [file 41586_2023_6393_MOESM1_ESM.pdf]

nature portfolio

https://doi.org/10.1038/s41586-023-06393-x

Supplementary information

Learning heterogeneous reaction kinetics from X-ray videos pixel by pixel

In the format provided by the
authors and unedited

Learning heterogeneous reaction kinetics from X-ray movies pixel-by-pixel: Supplementary Information

Hongbo Zhao[1], Haitao Dean Deng[3], Alexander E. Cohen[1], Jongwoo Lim[3], Yiyang Li[3], Dimitrios Fraggedakis[1], Benben Jiang[1], Brian D. Storey[4], William C. Chueh[3], Richard D. Braatz[1], and Martin Z. Bazant *[1,2]

[1] *Department of Chemical Engineering, Massachusetts Institute of Technology, Cambridge, MA 02139*

[2] *Department of Mathematics, Massachusetts Institute of Technology, Cambridge, MA 02139*

[3] *Department of Materials Science and Engineering, Stanford University, Stanford, CA 94305*

[4] *Toyota Research Institute, Cambridge, MA 02139*

July 15, 2023

Contents

1 Experimental data **2**
- 1.1 Materials and methods . . . 2
- 1.2 Experimental uncertainty . . . 3
- 1.3 Data statistics . . . 4

2 Model **4**
- 2.1 Governing equations . . . 4
- 2.2 Chemomechanics . . . 6
- 2.3 Parameterization of free energy and exchange current . . . 7

3 Relaxation toward equilibrium **10**

4 Spatial heterogeneity **14**
- 4.1 Model . . . 14
- 4.2 Estimates of intra- and inter-particle variance . . . 18

5 Inference based on different objective functions **20**
- 5.1 Inference based on uniformity coefficient . . . 20
- 5.2 Comparison of different objective functions and the number of parameters . . . 26

*Corresponding author. bazant@mit.edu

6 **Optimization and numerical methods** 28
6.1 Objective function . . . 28
6.2 Data preprocessing . . . 29
6.3 Numerical solver . . . 30
6.4 Temporal accuracy . . . 31
6.5 Spatial accuracy . . . 33
6.6 Optimization algorithm . . . 37
6.7 Interpolation of average composition . . . 37
6.8 Function representation . . . 41
6.9 Boundary condition . . . 41

7 **Identifiability** 42

8 **Cross-validation** 45
8.1 Regularization . . . 45
8.2 Variance and bias of MAP estimate . . . 45
8.3 Sensitivity analysis of spatial heterogeneity . . . 48
8.4 Regularization of Z_0 and higher order parameters . . . 49
8.5 Method of regularization . . . 51
8.6 k-fold cross-validation . . . 52

9 **Validation** 57
9.1 Uncertainty quantification . . . 57
9.2 Comparison of spatial heterogeneity estimated using finite difference and inversion . . . 58
9.3 Comparison with auger electron microscopy (AEM) . . . 61
9.4 Comparison of different models . . . 64
9.5 Reaction kinetics . . . 65
9.6 Variation in concentration field . . . 68

10 **Inversion result** 68

1 Experimental data

1.1 Materials and methods

We briefly describe the details on materials synthesis, electrode assembly, and characterization. More details can be found in [1, 26].

Synthesis. $LiFePO_4$ platelets were synthesized using a solvothermal method. All the precursors were purchased from Sigma-Aldrich. 6 mL of 1 M H_3PO_4 was mixed with 24 mL of polyethylene glycol 400. Then, 18 mL of 1 M LiOH(aq) was added to precipitate Li_3PO_4. The mixture was constantly bubbled with dry N_2 at a flow rate of 50 mL/min for 16 h for deoxygenation. Next, 12 mL of deoxygenated H_2O was added via a Schlenk line to $FeSO_4 7H_2O$ powder, which was pre-dried under vacuum. The $FeSO_4$ solution was then injected into the Li_3PO_4 suspension without oxygen exposure, and the entire mixture was transferred to a 100-mL Teflon-lined autoclave. The autoclave was heated to 140 °C for 1 h and then to 210 °C for 17 h. The sample was annealed under Ar gas at 720 °C for 5 h to eliminate anti-site defects[55, 56].

In this study, we employed the platelet particle morphology for three reasons: (1) matching particle size to the spatial resolution of the microscope, (2) aligning the particles crystallographically to the X-ray beam, and (3) ensuring a short solid-state transport distance. These three considerations are essential for image version. Additionally, the platelet morphology is also used extensively for fundamental studies in the literature [57, 58, 41, 59, 60, 26].

Carbon coating. The white $LiFePO_4$ particles were mixed with sucrose at a ratio of 5:1 ($LiFePO_4$:sucrose). This sample was then heated to 600 °C for 5 hr in a tube furnace under flowing Ar to yield the carbon-coated $LiFePO_4$.

Structure and electrochemical characterization. The carbon-coated LFP particles were first electrochemically tested in standard CR2032 lithium half cells, yielding a specific capacity of 150mAh/g at a rate of C/10. For structural and morphological characterization, scanning electron microscopy (SEM), X-ray diffraction (XRD), and atomic force microscopy (AFM) analyses of the pristine LFP samples were performed (see Refs. [1, 26]).

STXM experiment. The STXM experiment was conducted at beam line 11.0.2.2 and 5.3.2.1 of Advanced Light Source (ALS). The operando STXM at 11.0.2.2 utilizes a 45-nm zone plate. High-resolution images were raster-scanned in 50-nm-steps, with a dwell time of 1 ms per pixel. The additional operando STXM at 5.3.2.1 utilizes 60-nm zone plate. For imaging, a single layer of 10,000 to 50,000 particles was dispersed on the working electrode of the liquid STXM imaging platform. During the experiment, Fe(II) and Fe(III) L_3 edge was repeatedly scanned to quantify the local oxidation state of Fe and ultimately Li concentration. STXM scans the LFP particles in 50nm steps, generating about 200 to 1400 pixels per particle, which is sufficient resolution for resolving spatial variation within single particles. The large, micron-sized LFP particles are separated from one another so individual particles can be clearly segmented out from the images. In addition, these LFP particles have a thin platelet morphology with a thickness of about 150 nm [1]. The morphology of the particles and the resolution of STXM imaging enables us to directly compare images taken in the a-c plane which reveals the depth-average Li concentration with our depth-averaged 2D single particle model [19, 7, 14, 24], perform the image inversion, and make inferences on the spatial heterogeneity.

AEM imaging. After the in-situ STXM experiment, the chip with the platelet LFP was transferred under Ar-sealed bags and directly imaged under a PHI 700 Auger Scanning Probe (ULVAC-PHI Inc.) with a depth sensitivity of up to 5 nm. Principles of AEM can be found in Refs. [61, 62]. First, we performed SEM to identify and ensure the particles to be analyzed were identical to the ones imaged under in-situ STXM experiment. Next, we obtain the auger electron spectra of a select area on the particle to identify the peak energy positions for C (270 eV), Fe (703 eV), O (510 eV) of the identified particle. Finally, elemental maps of the entire particle were obtained by monitoring the peaks of specific elemental energy transitions.

1.2 Experimental uncertainty

The depth-averaged Li fraction map is obtained from the X-ray absorption map via STXM imaging. As established previously [1, 63], the optical density $S \equiv -\ln I_X/I_{X0}$ (where I_X and I_{X0} are the intensity of the transmitted X-ray with and without the particle) at an energy level i (S_i) can be expressed as a linear combination of optical densities of pure LFP (O_{ia}) and FP (O_{ib}) based on the local composition: $S_i = O_{ia}a + O_{ib}b$, and $a/(a+b)$ is the Li fraction.

At least two energy levels are needed to uniquely determine a and b. To assess the uncertainty of the Li fraction, we measure the X-ray adsorption at 5 selected energy levels (700 eV, 706.2 eV, 706.8 eV, 711.2 eV, and 713.4 eV, covering 1 pre-edge, 2 rising edges, and 2 falling edges), we obtain an overdetermined system of equations in terms of a and b. Since physically we require $a > 0$ and $b > 0$, we then used a non-negative least square optimization algorithm to solve for a and b and hence the Li fraction. We perform bootstrapping, that is, randomly sampling multiple energy levels and use the corresponding optical density to solve for the Li fraction using the optimization algorithm. From the ensemble of bootstrapping samples, we obtain an estimate for the variance of the Li fraction for each pixel σ_{pixel}. The result is plotted in Fig. S1, where we show the maps of σ_{pixel} for each particle used for the uncertainty analysis, which have different states of charge, as well as the histogram of σ_{pixel} over all pixels and particles. The average of σ_{pixel} is 0.072.

Previously in Ref. [1], an estimate of the experimental error on Li fraction is obtained by calculating the standard deviation of the Li fraction values of all pixels in the reference images, in which the average Li fractions of the particles are 0.97 and 0.03, and the particles have uniform Li concentration field. The standard deviation is 0.06. Based on the bootstrapping result and the standard deviation of Li fraction in the reference images, we estimate that the standard deviation of the experimental error of Li fraction is about $\sigma_\epsilon \approx 0.07$.

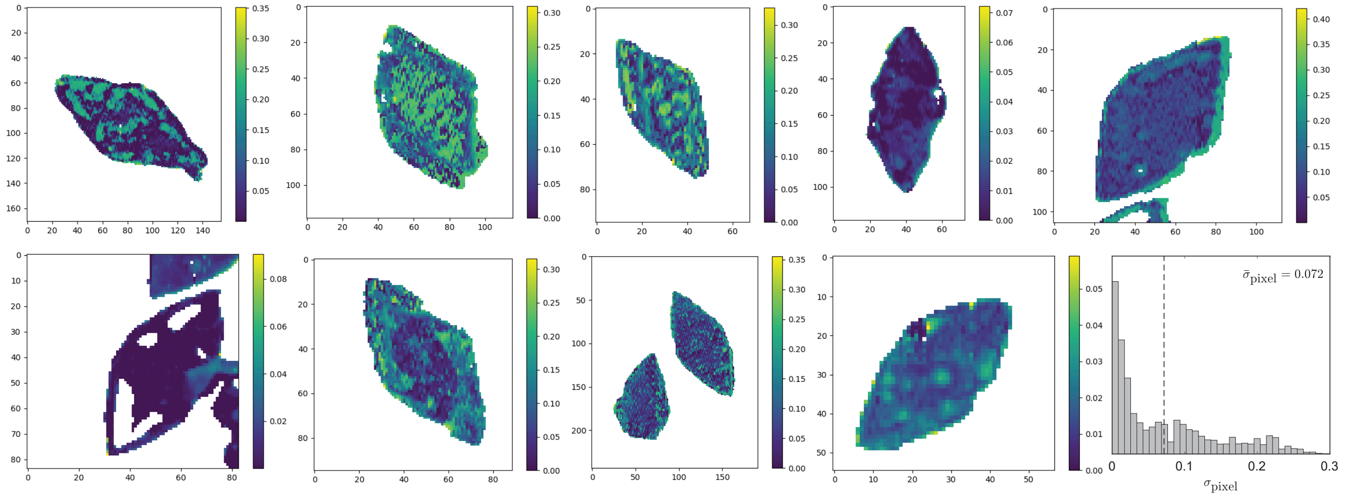

Figure S1: The map of standard deviation of Li fraction σ_{pixel} obtained from bootstrapping and the histogram.

1.3 Data statistics

Table S1 describes the statistics of the in-situ STXM images. Some particles are imaged over multiple charge and/or discharge cycles at different rates. In the Supplementary Information, each sequence of images during charge or discharge is called an *episode*. Fig. S2 shows the histograms of the number of frames per episode, pixels of each particle, particle size, and average rate of each episode, which is defined as the change in average Li fraction (from the first to last frames) divided by the duration of the episode. The median number of pixels and major axis length are 458.5 and 1.60 μm while the corresponding mean values are 458.75 and 1.65 μm. The width of a pixel is 50 nm[1].

2 Model

2.1 Governing equations

The free energy of lithium iron phosphate in the bulk can be described variationally as consisting of chemical free energy and mechanical energy (assumed to be linear elasticity),

$$G = \int_D \left(c_s \left(g_h(c) + \frac{1}{2}\kappa |\nabla c|^2 \right) + \frac{1}{2}\lambda_{ijkl}\varepsilon_{ij}^{\text{el}}\varepsilon_{kl}^{\text{el}} \right) dV, \tag{S1}$$

where D is the particle domain, c_s is the maximum concentration of lithium, or the lattice site density, c is the Li fraction (the fraction of lattice sites occupied by Li) $0 \leq c \leq 1$, $g_h(c)$ is the homogeneous free energy, λ_{ijkl} is the stiffness tensor, and the elastic strain is

$$\varepsilon_{ij}^{\text{el}} = \frac{1}{2}(u_{i,j} + u_{j,i}) - \varepsilon_{ij}^{0} c(\mathbf{x}), \tag{S2}$$

Table S1: Statistics of the dataset

Total number of particles	39	Total number of frames	343
Number of particles with 1 episode	21	Average number of pixels	486
Number of particles with 2 episodes	12	Total number of pixels (all frames)	186245
Number of particles with 3 episodes	6	Total number of pixels (all frames except the initial)	155082
Number of charge episodes	29	Number of discharge episodes	34

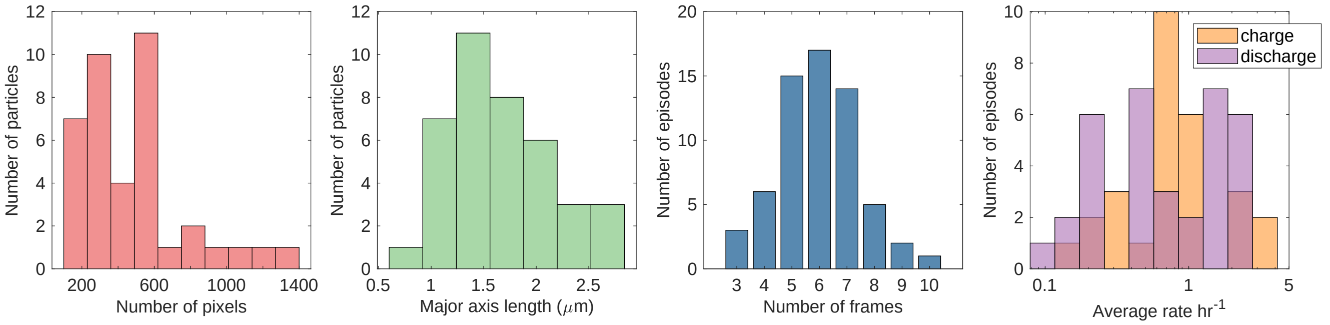

Figure S2: Statistics of the datasets. From left to right: the histogram of the number of frames per episode, the number of pixels of each particle, the major axis length of each particle, and the average rate of each episode.

where u is the displacement field, $u_{i,j}$ denotes the derivative of the ith component of the displacement in the jth direction, and the second term is the chemical strain due to the change in lattice parameters (measured in Ref. [26]), which is approximately proportional to the local lithium concentration. ε^0_{ij} is known as the stress-free strain, or misfit strain. Since the relaxation time for mechanical deformation is fast for solids, we assume mechanical equilibrium at all times, that is the variational derivative $\delta G/\delta u_i = 0$, or equivalently

$$\nabla \cdot \boldsymbol{\sigma} = 0, \tag{S3}$$

where the stress is (using Einstein notation)

$$\sigma_{ij} = \lambda_{ijkl}\varepsilon^{\text{el}}_{ij} = \lambda_{ijkl}u_{k,l} - \sigma^0_{ij}c(\mathbf{x}), \tag{S4}$$

where we define $\sigma^0_{ij} = \lambda_{ijkl}\varepsilon^0_{kl}$. The chemical potential is defined by the variational derivative

$$\mu = \frac{1}{c_s}\frac{\delta G}{\delta c} = \mu_h(c) - \kappa\nabla^2 c - c_s^{-1}\varepsilon^0_{ij}\sigma_{ij} \tag{S5}$$

$$= \mu_h(c) - \kappa\nabla^2 c + c_s^{-1}\sigma^0_{ij}(\varepsilon^0_{ij}c - u_{i,j}). \tag{S6}$$

Li inserts and de-inserts from the a-c plane. When LFP nanoparticles are thin in the b direction (or depth direction), we may assume that phase separation does not occur in the depth direction and, due to fast diffusion in the b direction, we assume that the Li concentration is uniform in the depth direction [7, 19, 24]. This effectively reduces the model to 2D, where we use a depth-averaged concentration c in the a-c plane, and locally the rate of change of c is governed by the (de)lithiation reaction rate. Diffusion in the a-c plane is typically slow and can occur via defects or the surface layer [64, 60]. Neglecting the lateral diffusion, the local rate of change of the Li fraction results from the surface reaction,

$$\frac{\partial c}{\partial t} = R, \tag{S7}$$

where we model the reaction rate R by Butler-Volmer kinetics for electrochemical reactions for the purpose of inverting the unknown $j_0(c)$ and later compare it with coupled electron transfer theory (CIET). The Butler-Volmer rate formula for R is [65]

$$R = j_0(c)\left(e^{-\alpha\tilde{\eta}} - e^{(1-\alpha)\tilde{\eta}}\right), \tag{S8}$$

where $\tilde{\eta} = \eta e/(k_B T)$ is the nondimensionalized overpotential, η is the overpotential and $k_B T\tilde{\eta} = \mu - \mu_{\text{res}}$, where $\mu_{\text{res}} = \mu_{\text{Li}^+} - e\Delta\phi$ is known as the reservoir chemical potential, and μ_{Li^+} and $\Delta\phi$ are the chemical potential of Li^+ in the electrolyte and the interfacial voltage, respectively.

The natural boundary condition for c satisfies the minimization of the sum of bulk energy Eq. S1 and interfacial energy at the particle boundaries $\int_{\partial D}\gamma dS$,

$$\mathbf{n} \cdot \nabla c = -\frac{1}{\kappa}c_s \cdot \frac{d\gamma}{dc}. \tag{S9}$$

Without the knowledge of the interfacial energy, we impose Dirichlet boundary condition for c based on the image data. We use the no traction boundary condition for Eq. S3,

$$\mathbf{n} \cdot \boldsymbol{\sigma} = 0. \tag{S10}$$

2.2 Chemomechanics

In this article, the mechanical parameters for LFP are given by literature values. The misfit strain in the a-c plane is approximated by a linear dependence on Li concentration, based on the result inverted from ptychography and the strain map [26]. In the b direction, we use literature value of the lattice parameter 4.784 Å for $FePO_4$ and 4.659 Å for $LiFePO_4$.The stiffness tensor is that of $FePO_4$ from first-principles calculation using the GGA+U method [40]. Using Voigt notation, $C_{11} = 175.9$, $C_{22} = 153.6$, $C_{33} = 135.0$, $C_{44} = 38.8$, $C_{55} = 47.5$, $C_{66} = 55.6$, $C_{12} = 29.6$, $C_{13} = 54.0$, and $C_{23} = 19.6$.

For particles that are thin in the [010] direction, a 2D depth-averaged model in (010) plane is shown to capture the particle behavior well. Following Ref. [20], we use the plane strain condition. Under mechanical equilibrium, Eq. S3 can be written as

$$\lambda_{ijkl} u_{k,lj} = \sigma^0_{ij} \partial_j c, \tag{S11}$$

where $u_{k,lj} = \partial_{lj} u_k$. This equation can be solved analytically in Fourier space, in an infinite domain. Following [25],

$$\hat{u}_k = E_{ik} \sigma^0_{ij} k_j \Delta\hat{c}, \tag{S12}$$

where $\hat{u_i}$ is the Fourier transform of the heterogeneous displacement field (the total displacement subtracted by the average), $\bar{c}$ is the average concentration, $\Delta\hat{c}$ is the Fourier transform of $c - \bar{c}$, and

$$\left(E^{-1}\right)_{ik} = \lambda_{ijkl} k_j k_l. \tag{S13}$$

where $\mathbf{k}$ is the wavevector in Fourier space. Substituting the solution to the displacement into the total free energy results in

$$G = \int_V c_s \left(g_h(c) + \frac{1}{2} \kappa |\nabla c|^2 \right) d\mathbf{r} + \frac{1}{2(2\pi)^d} \int_{\hat{V}} B(\mathbf{n}) |\Delta\hat{c}|^2 d\mathbf{k}, \tag{S14}$$

where d is the dimensionality, $\mathbf{n} = \mathbf{k}/|\mathbf{k}|$ is the unit vector, and

$$B(\mathbf{n}) = \lambda_{ijkl} \varepsilon^0_{ij} \varepsilon^0_{kl} - n_i \sigma^0_{ij} U_{jl}(\mathbf{n}) \sigma^0_{lm} n_m, \tag{S15}$$

where

$$\left(U^{-1}\right)_{ik} = \lambda_{ijkl} n_j n_l. \tag{S16}$$

Therefore, the minimum mechanical energy is obtained when the concentration field has zero components other than the optimal direction $\mathbf{n}^* = \operatorname{argmin} B(\mathbf{n})$ in Fourier space. It has been shown that the minimum energy for LFP corresponds to the LFP-FP interface being approximately in the direction of [101] [20]. Therefore, in an infinitely large, coherent, relaxed, and phase-separated (FP and LFP are in coexistence) particle, the total free energy can also be written as

$$G \approx \int_V c_s \left(g_h(c) + \frac{1}{2} B_{\min} (c - \bar{c})^2 + \frac{1}{2} \kappa |\nabla c|^2 \right) d\mathbf{r}, \tag{S17}$$

where $B_{\min} = \min_{\mathbf{n}} B(\mathbf{n})$. For LFP, $B_{\min} = 0.19$ GPa [36]. In the literature, when the lithium poor and rich phases occur in different particles, the elastic energy is zero, since the Li concentration field in the particles is uniform. In this case, the equilibrium composition of $Li_\delta FePO_4$ is called the *mosaic miscibility gap* $[c_1, c_2]$[20]. This equilibrium state minimizes the chemical free energy and satisfies the following criteria, also known as Maxwell or common tangent construction,

$$g_h(c_2) - g_h(c_1) = \mu_h (c_2 - c_1) \tag{S18}$$

$$\mu_h(c_1) = \mu_h(c_2). \tag{S19}$$

When the two coexisting phases are in the same particle and remain mechanically coherent, the compositions are called the *coherent miscibility gap*, which minimizes the free energy Eq. S17. If we define $g_{h,c}(c) = g_h(c) + \frac{1}{2}B_{\min}(c - \bar{c})^2$ and chemical potential $\mu_{h,c}(c) = \mu_h(c) + \frac{1}{2}B_{\min}(c - \bar{c})$, the equilibrium compositions $c_{1,c}$ and $c_{2,c}$ satisfy

$$g_{h,c}(c_{2,c}) - g_{h,c}(c_{1,c}) = \mu_{h,c}(c_{2,c} - c_{1,c}) \tag{S20}$$

$$\mu_{h,c}(c_{1,c}) = \mu_{h,c}(c_{2,c}). \tag{S21}$$

2D simulations are performed in the image coordinates. Since the particles are randomly oriented, we need to perform a coordinate transformation of the aforementioned mechanical parameters from the crystallographic coordinate system to the image coordinate system. The rotation matrix is

$$Q = \begin{bmatrix} \cos\theta & \sin\theta \\ -\sin\theta & \cos\theta \end{bmatrix}, \tag{S22}$$

where θ is the counterclockwise angle from crystallographic a-c coordinates to image x-y coordinates. For ptychography images [26] with the electron diffraction measurement, the a-c directions are known from the diffraction measurement. For STXM images where only the concentration profile is available, the a-c directions need to be estimated. Wulff reconstruction shows that a perfect and energetically stable LFP crystal is platelike and with the major and minor axes of the plane being [001] and [100], as confirmed in experiments [66, 67, 41]. Therefore, for particles imaged by STXM, we assume that a-c axis coincides with the minor and major axes of the particle, defined by the principal axes of the particle based on the central second moment of the particle shape (this assumption is revisited in Section 3).

The misfit strain in the image coordinate is

$$\epsilon^{0\prime}_{ij} = Q_{ik}Q_{jl}\epsilon^0_{kl}, \tag{S23}$$

and the stiffness tensor is

$$\lambda'_{ijkl} = Q_{im}Q_{jn}Q_{ko}Q_{lp}\lambda_{mnop}. \tag{S24}$$

2.3 Parameterization of free energy and exchange current

In the literature, the regular solution model is typically used to model the homogeneous chemical free energy,

$$\frac{g_h(c)}{k_BT} = c\ln c + (1-c)\ln(1-c) + \Omega c(1-c). \tag{S25}$$

In Ref. [20], $\Omega = 4.47$, which corresponds to a mosaic miscibility gap of $c_1 = 0.0126$ ($c_2 = 1 - c_1$ due to symmetry), and a coherent miscibility gap of $c_{1,c} = 0.0927$ ($c_{2,c} = 1 - c_{1,c}$). Other parameters that we use from the literature are [20]: $\kappa = 5.02\times10^{-10}$ J/m, maximum Li concentration $c_s = 2.29\times10^4$ mol/m^3. At $T = 298$ K, $\kappa/(RTc_s) = 8.847\times10^{-18}$ m^2. When normalized by STXM pixel size ($L = 50$ nm [1]), $\tilde{\kappa} = \frac{\kappa}{RTc_sL^2} = 3.54\times10^{-3}$.

For the inversion, we consider two choices for representing the chemical potential. One option has an ideal entropic term and a polynomial function that corresponds to the excess part, which follows the physical constraint that the concentration is within $[0,1]$. In the following text, the chemical potential $\mu_h(c)$ is nondimensionalized by k_BT, that is, $\tilde{\mu}_h(c) = g'_h(c)/k_BT$. For simplicity, we drop the tilde and

$$\mu_h(c) = \mu_{\rm id}(c) + \mu_{\rm ex}(c) = \ln\frac{c}{1-c} + \sum_{n=1}^{N_\mu} a_nP_n(c), \tag{S26}$$

where $P_n(c)$ are Legendre polynomials defined on $[0,1]$. The second option consists only of the polynomials,

$$\mu_h(c) = \sum_{n=1}^{N_\mu} a_nP_n(c), \tag{S27}$$

We constrain the chemical potential such that the mosaic miscibility gap is $[c_1, c_2]$ (using values in [20] as mentioned above),

$$\int_{c_1}^{c_2} \mu_h(c)dc = \mu_h(c_1)(c_2 - c_1) \tag{S28}$$

$$\mu_h(c_1) = \mu_h(c_2). \tag{S29}$$

The constraint corresponds to a linear constraint on the parameters a_n.

Because we do not have the local voltage data $\Delta\phi$ in our study since the local voltage losses are unknown, a constant shift in $\mu_h(c)$ is equivalent. Hence we may fix the chemical potential at the mosaic miscibility gap to be 0, $\mu_h(c_1) = \mu_h(c_2) = 0$. To illustrate the constrained representation of $\mu_h(c)$, Fig. S3 shows the family of $\mu_h(c)$ curves when we allow one degree of freedom (1-parameter representation). Using the lowest order polynomial possible, because there are three linear constraints, the highest order is a third-order polynomial. Since the derivative of Ginzburg-Landau free energy contains up to a third-order polynomial, the former choice of Eq. S26 corresponds to a regular solution model plus a rescaled Ginzburg Landau chemical potential (multiplied by a coefficient),

$$\mu_h(c) = \ln \frac{c}{1-c} + \Omega(1-2c) + p_{\mathrm{GL}}\mu_{\mathrm{GL}}\left(\frac{c-c_1}{c_2-c_1}\right), \tag{S30}$$

The latter choice of Eq. S27 corresponds to a rescaled Ginzburg Landau chemical potential above (multiplied by a coefficient),

$$\mu_h(c) = p_{\mathrm{GL}}\mu_{\mathrm{GL}}\left(\frac{c-c_1}{c_2-c_1}\right). \tag{S31}$$

In Fig. S3, we also show $\mu_{h,c}(c) = \mu_h(c) + B_{\min}(c - 0.5)$. At the same coherent spinodal barrier height, the Ginzburg-Landau free energy (Eq. S31 gives a smaller miscibility gap than the regular solution representation (Eq. S30, it also has a smaller unstable (spinodal) region where $d\mu_h/dc < 0$. The coherent spinodal point is defined by $d\mu_{h,c}/dc = 0$.

Next, we constrain the coherent miscibility gap, that is,

$$\int_{c_{1,c}}^{c_{2,c}} \mu_{h,c}(c)dc = \mu_{h,c}(c_{1,c})(c_{2,c} - c_{1,c}) \tag{S32}$$

$$\mu_{h,c}(c_{1,c}) = \mu_{h,c}(c_{2,c}), \tag{S33}$$

which adds two additional linear constraints. Fig. S4 shows the family of $\mu_h(c)$ and $\mu_{h,c}$ curves when we allow one degree of freedom with the entropic term. In this case, the highest order is a fifth-order polynomial.

Using the first approach that includes the ideal entropy (Eq. S26), we assume the prior for the nonideal part of the (excess) chemical potential follows a Gaussian distribution $\mu_{\mathrm{ex}}(c) \sim \mathcal{N}(0, \sigma^2_{\mathrm{global}}\delta(c-c'))$; alternatively when using the second approach (Eq. S27), assume $\mu_h(c) \sim \mathcal{N}(0, \sigma^2_{\mathrm{global}}\delta(c-c'))$. Due to the orthogonality of Legendre polynomials, the prior for a_n is

$$P(\mathbf{a}) \propto \exp -\frac{\|\mathbf{a}\|_2^2}{2\sigma^2_{\mathrm{global}}} = \exp -\frac{1}{2\sigma^2_{\mathrm{global}}} \sum_{n=1}^{N_\mu} a_n^2. \tag{S34}$$

For the exchange current $j_0(c)$, we consider two parameterizations. To ensure its positivity,

$$j_0(c) = \exp \sum_{n=0}^{N_J} b_n P_n(c). \tag{S35}$$

Because electron transfer requires the availability of vacancy in the lattice and, based on transition state theory and coupled-electron transfer theory [29], j_0 is expected to approach 0 as c approaches 0 and 1; therefore, we consider the representation

$$j_0(c) = c(1-c) \exp \sum_{n=0}^{N_J} b_n P_n(c). \tag{S36}$$

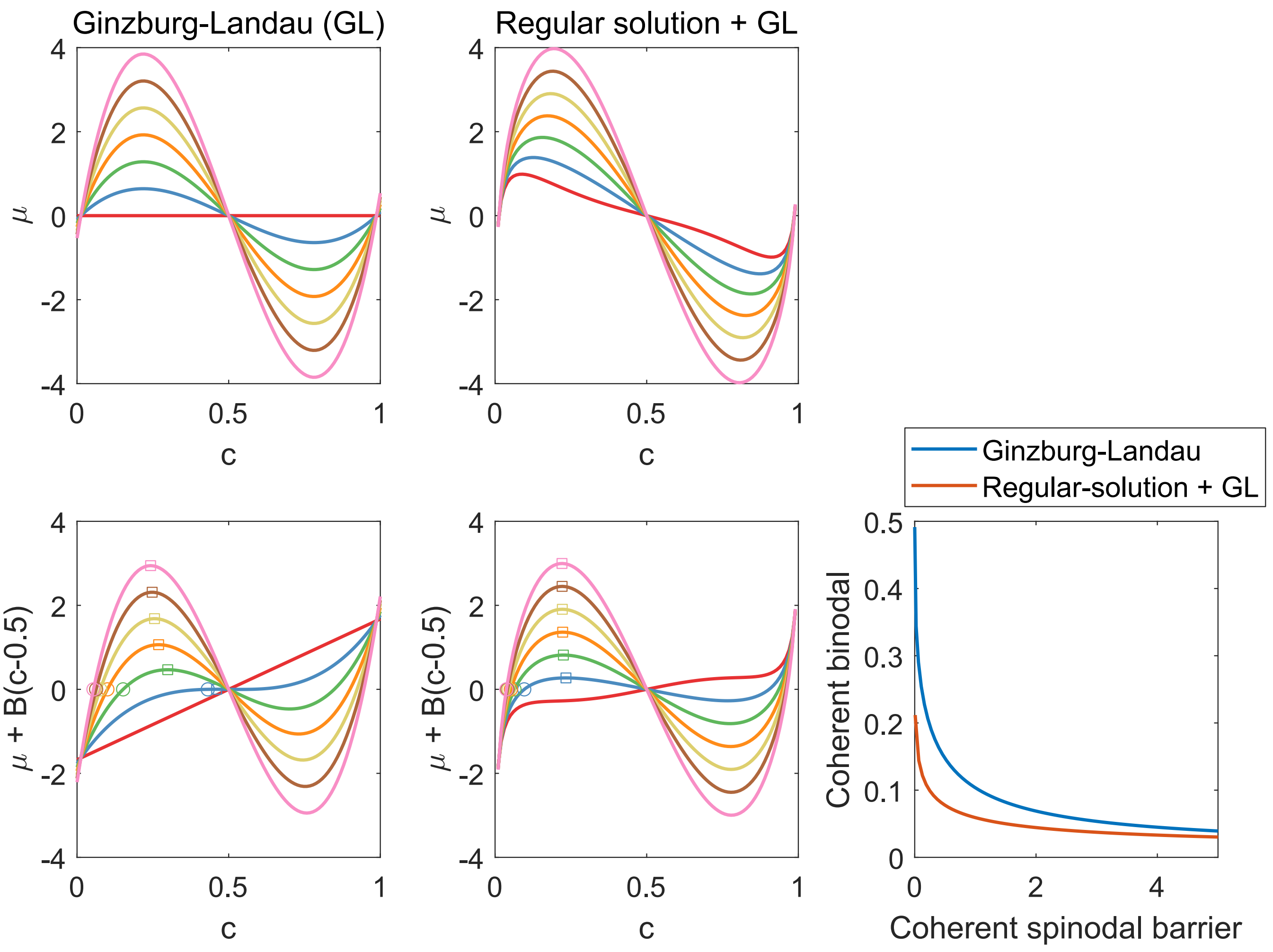

Figure S3: $\mu_h(c)$ (first row) and $\mu_{h,c}(c)$ (second row) with one degree of freedom with a fixed mosaic miscibility gap, as seen in the first row. The lower right figure plots the coherent spinodal barrier ($\mu_{h,c}(c)$ at the spinodal point, squares on $\mu_{h,c}(c)$) versus coherent binodal point (circles on $\mu_{h,c}(c)$).

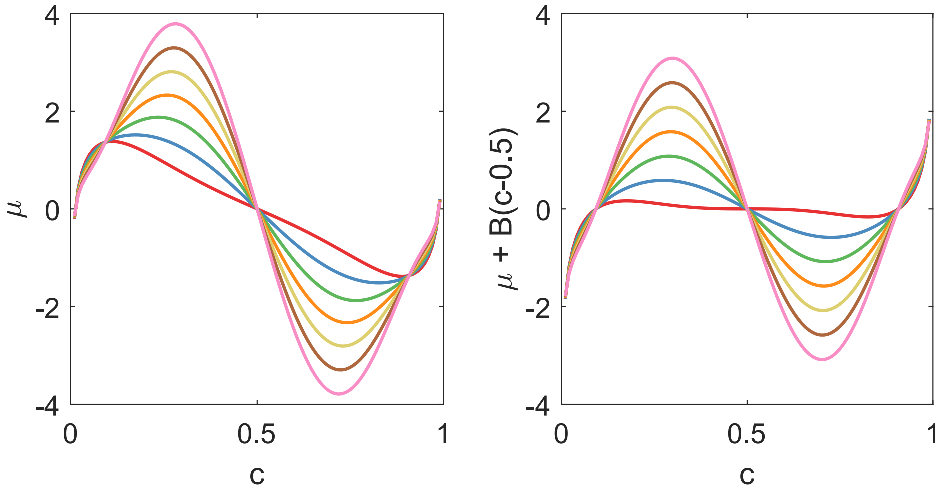

Figure S4: $\mu_h(c)$ (left) and $\mu_{h,c}(c)$ (right) with one degree of freedom with fixed mosaic and coherent miscibility gaps.

Similar to the chemical potential, we suppose the prior for $j_0(c)$ is

$$P(\mathbf{b}) \propto \exp -\frac{\|\mathbf{b}\|_2^2}{2\sigma_{\text{global}}^2} = \exp -\frac{1}{2\sigma_{\text{global}}^2}\sum_{n=1}^{N_\mu} b_n^2, \tag{S37}$$

See Section 5.2 for the choice of the number of parameters used in the inversion.

3 Relaxation toward equilibrium

To validate the chemomechanical model using images of relaxed particles, we simulate the relaxation toward equilibrium under the condition that the average composition of the particles remains constant in time. The variation of the total free energy (including the surface energy) is

$$\delta G = \int_D \left(c_s\left(\mu_h(c)\delta c - \kappa\nabla^2\delta c + \sigma_{ij}^0\left(\varepsilon_{ij}^0 - \varepsilon_{ij}\right)\right)\delta c\right)dV + \int_{\partial D}(\kappa\mathbf{n}\cdot\nabla c + \gamma'(c))\delta c dS; \tag{S38}$$

if the natural boundary condition $\kappa\mathbf{n}\cdot\nabla c + \gamma'(c) = 0$ is used, then

$$\delta G = c_s\int_D \mu\delta c dV. \tag{S39}$$

For a large particle, the surface energy has a very small contribution, hence we show the free energy without the surface energy term. When the system is governed by reaction with the external reservoir,

$$\frac{\partial c}{\partial t} = R(c, \mu_{\text{res}} - \mu), \tag{S40}$$

with the constraint that $d\int_V c dx/dt = 0$, and μ_{res} changes in time to satisfy the constraint, therefore,

$$\frac{1}{c_s}\frac{\partial G}{\partial t} = \int_V \mu\frac{\partial c}{\partial t}dx = \int_V (\mu - \mu_{\text{res}})\frac{\partial c}{\partial t}dx = \int_V (\mu - \mu_{\text{res}})R dx. \tag{S41}$$

Butler-Volmer kinetics satisfies the condition that when $\mu_{\text{res}} - \mu > 0$ (known as chemical affinity), $R > 0$, hence $dG/dt < 0$. Since the free energy decreases monotonically in time, we compute the free energy to monitor the relaxation process and verify whether the system has reached equilibrium, that is, the local minimum of the free energy. Because relaxation mostly occurs outside the electrolyte and happens at a much longer time scale than reaction, we also consider modeling relaxation via lateral diffusion,

$$\frac{\partial c}{\partial t} = \nabla\cdot L\nabla\mu, \tag{S42}$$

In this case, the free energy of the system follows

$$\frac{1}{c_s}\frac{\partial G}{\partial t} = \int_V \mu\frac{\partial c}{\partial t}dx = \int_V -\mu\nabla\cdot\mathbf{F}dx = -\int_{\partial V}\mu\mathbf{F}\cdot\mathbf{n}dS - \int \nabla\mu\cdot L\nabla\mu dV = -\int \nabla\mu\cdot L\nabla\mu dV, \tag{S43}$$

where the flux $\mathbf{F} = -L\nabla\mu$, and the flux is zero on the boundary. Since L is positive definite, the free energy also decreases monotonically $dG/dt < 0$.

We consider three scenarios of achieving equilibrium: starting from the experimentally observed concentration field as the initial condition, 1) relax via diffusion, 2) relax via reaction, and 3) starting from a uniform concentration field with the same composition as the average of the experimentally observed concentration fields and relax via reaction. We do not consider relaxation from a uniform concentration field via diffusion because of the formation of spinodal patterns, whose wavelength is much smaller than the particle size and hence will take a long physical relaxation time and computational time (note that with diffusion, the domain size coarsens as $t^{1/3}$ while with reaction the coarsening follows $t^{1/2}$). Essential boundary condition for the concentration field is imposed in all

scenarios, that is, the boundary values of $c(x)$ are set to be equal to the experimentally observed values throughout the entire simulation.

The relaxation time in the following figures is nondimensionalized. When driven by reaction, the dimensionless time is tk_0, where k_0 is the scale for the exchange current density. When driven by diffusion, the dimensionless time is tD_0/L^2, where $L = 300$ nm is chosen such that reaction and diffusion relaxation be compared on the same dimensionless time axis. When reporting the free energy, we subtract the following free energy which corresponds to a phase-separated, infinitely large, coherent, and relaxed particle, without the interfacial energy,

$$G_{\mathrm{ref}} = V_1 g_{h,c}(c_{1,c}) + (1 - V_1) g_{h,c}(c_{2,c}), \tag{S44}$$

where we have previously defined $g_{h,c}(c) = g_h(c) + \frac{1}{2}B_{\min}(c - \bar{c})^2$ as the sum of chemical free energy and bulk elastic energy. $c_{1,c}$ and $c_{2,c}$ are the coherent miscibility gap. V_1 is the fraction of phase 1, therefore, we have $c_{1,c}V_1 + c_{2,c}(1 - V_1) = \bar{c}$. At later stages of the coarsening, the reported free energy $G - G_{\mathrm{ref}}$ is the sum of interfacial and edge elastic energy [25]. The model parameters are described in Section 2.

Fig. S5 shows the relaxation process of one particle via the three scenarios up to dimensionless time (tk_0 or tD_0/L^2) 5000. 4 selected frames from the simulations are shown next to the experimental image and the selected frames are highlighted as black dots in the plots of free energy versus dimensionless time. The interface in the final frame is juxtaposed with the experimental image, as well as the free energy in time. The experimental images are from Ref. [26]. Based on electron diffraction measurements, the a-c crystallographic axes of these particles are known. The maximum mesh size is 5 nm, the same as the pixel size, which is sufficiently fine as explained in Section 6.5. Fig. S6 compares the experiment and the interface at equilibrium from simulation (white lines) and summarizes the evolution of free energy in time for each method and particle.

We find that relaxation starting from the experimental images either via diffusion or reaction results in similar final patterns (except for lateral displacement and mirror symmetry) and agrees well with the experimental observation. The final free energy reflects the interfacial area; for example, for particle 2, there are two LFP domains for scenarios 1 and 2 whereas, for particle 3, LFP has merged into one domain and has a lower free energy than the former.

In the simulations above, essential boundary conditions are used for all scenarios. To illustrate the effect of boundary condition, we perform simulations using the non-wetting boundary condition ($\mathbf{n} \cdot \nabla c = 0$) and let the system relax via reaction from the experimental concentration field and compare it with scenario 2. Fig. S7 shows that, for most particles, the boundary condition does not have any significant influence on the final geometry of the bulk LFP or FP domains except for particle 2, in which two LFP domains are still undergoing coarsening to merge into one. The difference between the two boundary conditions is usually confined to within a very short distance (interfacial thickness) from the interface, for example, the zoom-in views of particles 1 to 4 show a thin FP layer between the LFP domain in the bulk and the particle boundary, the zoom-in view of particle 5 shows a particle boundary wetted by LFP. Therefore, the influence of the boundary condition is typically within a distance of the interfacial thickness away from the boundary and a much smaller length scale than the particle size.

In the image data above, the crystallographic orientation is known, as a result, the relaxed LFP-FP interface predicted by the model (based on the misfit strain measured in Ref. [26] matches the experiment nearly exactly. With in-situ STXM images, the crystallographic orientation is unknown. In the relaxation simulations below, we assume that the c axis aligns with the major axis of the particle. We have shown above that the difference in the equilibrium shape between essential and natural conditions is minor. Hence in the simulations below, we show only the results using essential boundary condition. Here the maximum mesh size is also set to 5 nm, or 1/7 of the pixel size, fine enough to resolve the interface. We find in Figs. S8 and S9 that, regardless of the mode of relaxation (reaction from experimental or uniform initial condition, or diffusion from experimental initial condition), the final pattern of phase separation is similar to the experimental image. 7.9° and 10.0° deviation is observed between the simulated and experimental LFP-FP interface for particles 3 and 4, which shows the error due to the unknown crystallographic orientation.

Lastly, we comment on the interfacial thickness. The value of interfacial thickness reported in the literature does vary[68, 69, 70, 71], Ref. [70] reports a thickness of 12 nm based on STEM/EELS, while Ref. [71] reports a thickness of about 75 nm for the (210) interface and about 25 nm for the (100) interface. In our datasets, the

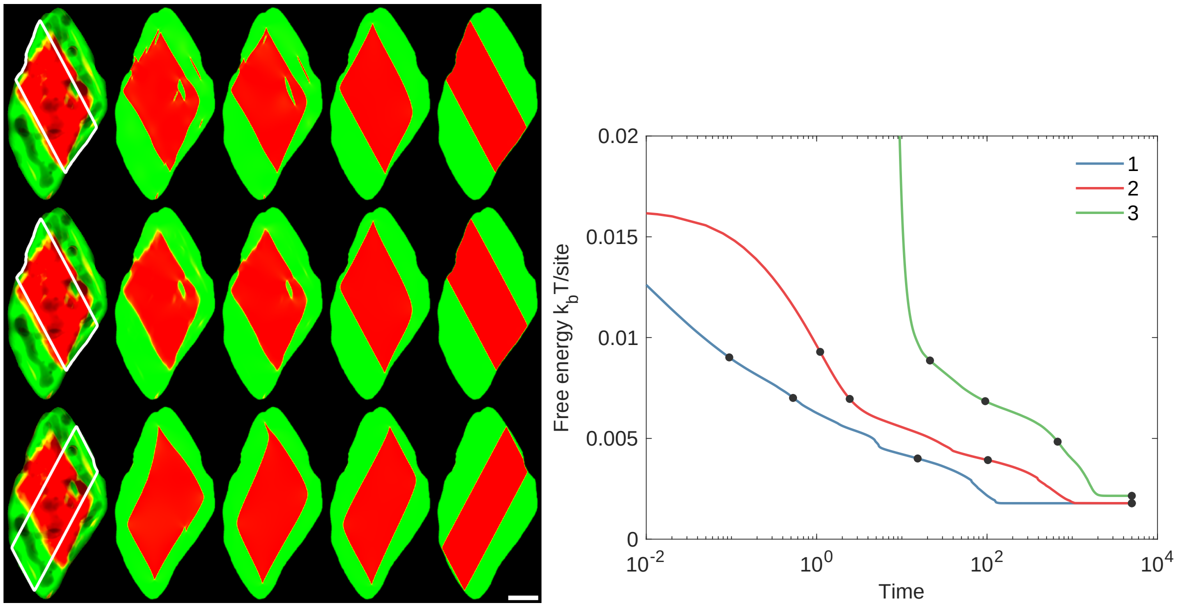

Figure S5: The experimental image of Li concentration field in relaxed LFP particles, and the simulated concentration field over time. The LFP-FP interface from the simulation at the last time step is shown on the experimental image as the white curve for comparison. The free energy evolution is plotted for all relaxation scenarios. Legend 1, 2, and 3 refer to the relaxation via diffusion, via reaction (starting from experimental image), and relaxation via reaction (starting from a uniform concentration field), and correspond to the 1st, 2nd, and 3rd rows. Black dots in the free energy curves correspond to the simulation snapshots shown. The scale bar is 500 nm. The image horizontal and vertical axes correspond to crystallographic a and c axes.

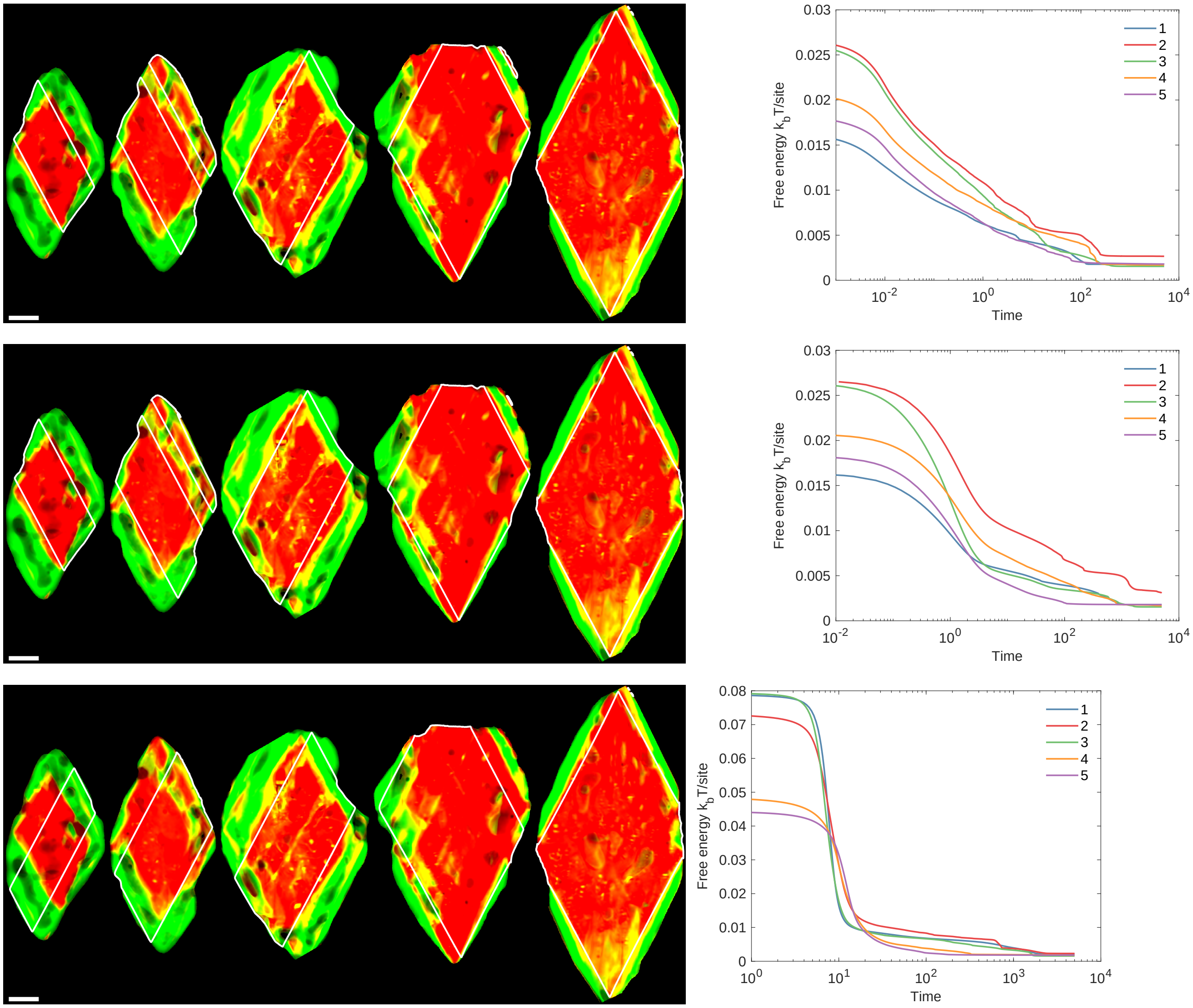

Figure S6: Similar to Fig. S5. The LFP-FP interface from the simulation at the last time step is shown on the experimental image as the white curve for comparison. From top to bottom, simulations correspond to relaxation via diffusion, via reaction (starting from experimental image), and relaxation via reaction (starting from a uniform concentration field). Legend names 1 to 5 in the free energy curves correspond to particles from left to right. The scale bar is 500 nm.

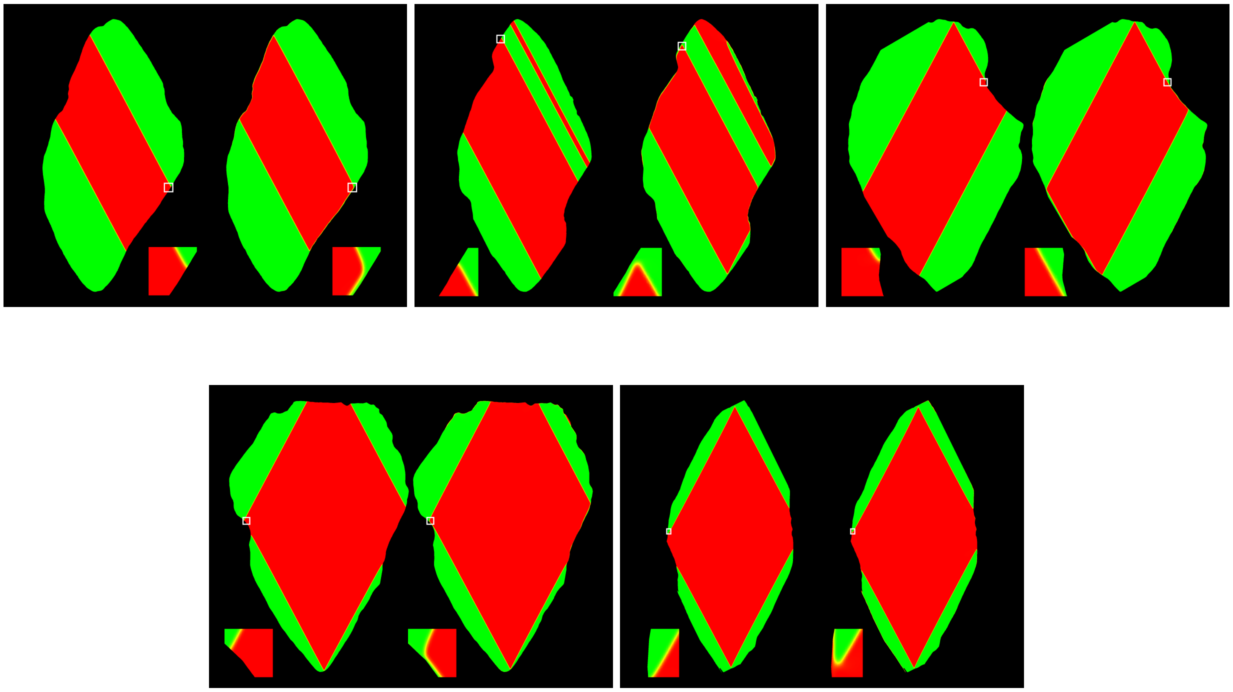

Figure S7: Comparison of non-wetting boundary condition (left) and essential boundary condition (right, based on experimental images) for concentration. Relaxation occurs via reaction and the results at a dimensionless time of 5000 are shown. The inset is a zoom-in view of the highlighted region in the main image. All highlighted rectangles are 100 nm × 100 nm in size and are identical regions in space for the same particle except for particle 2, which are shifted to highlight the LFP-FP-boundary junction.

interfacial thickness estimated from the STXM images is about 70 nm, and that estimated from the ptychography images is about 123 nm [26]. The variation can come from an interface that is not fully relaxed, or its orientation not being perpendicular to the imaged a-c plane. The parameters we chose following Ref. [20] give a simulated interfacial thickness of 12 nm. We see above that although the simulated interfacial thickness differs from the experiments, the phase separation morphology agrees well with the experiments. In the following sections, we are most interested in the regime where the Li concentration gradient is not as large because particles actively undergo Li insertion and de-insertion and do not fully relax, hence the value of κ is expected to have an even smaller effect and we will use the same κ value as described in sec. 2.3.

4 Spatial heterogeneity

4.1 Model

As mentioned in the main text, we use a multiplicative prefactor $k(\mathbf{x})$ ($\mathbf{x}$ corresponds to (x, y) in the main text) to model the spatial heterogeneity in the reaction kinetics, that is,

$$\frac{\partial c}{\partial t} = k(\mathbf{x}) j_0(c) \left(e^{-\alpha\tilde{\eta}} - e^{(1-\alpha)\tilde{\eta}} \right). \tag{S45}$$

The prior that we choose for $\psi(\mathbf{x}) = \ln k(\mathbf{x})$ is $(\psi(\mathbf{x}) - \psi_0)|\psi_0 = \psi(\mathbf{x}) - \psi_0 \sim \mathcal{N}(0, C)$, and $\psi_0 \sim \mathcal{N}(0, \sigma_{\psi_0}^2)$, where

$$C(\mathbf{x}_1, \mathbf{x}_2) = \sigma_\psi^2 \exp -\left[\frac{\|\mathbf{x}_1 - \mathbf{x}_2\|^2}{2l^2} \right]. \tag{S46}$$

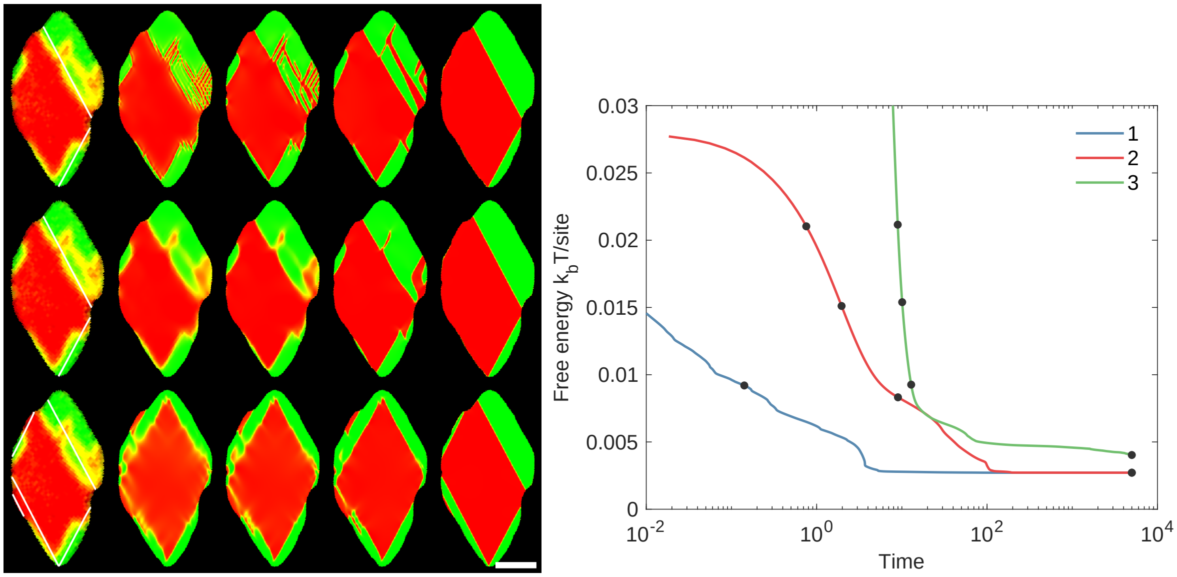

Figure S8: The experimental image of Li concentration field in relaxed LFP particles, and the simulated concentration field over time. The LFP-FP interface from the simulation at the last time step is shown on the experimental image as the white curve for comparison. The free energy evolution is plotted for all relaxation scenarios. Legend 1, 2, and 3 refer to the relaxation via diffusion, via reaction (starting from experimental image), and relaxation via reaction (starting from a uniform concentration field), and correspond to the 1st, 2nd, and 3rd rows. Black dots in the free energy curves correspond to the simulation snapshots shown. The scale bar is 500 nm. The image's horizontal and vertical axes correspond to crystallographic a and c axes.

Therefore, the prior of $\psi(\mathbf{x})$ is a Gaussian random field plus a constant that follows a normal distribution independently. The random field $\psi(\mathbf{x})$ has the properties

$$\mathbb{E}\left[\int \psi(\mathbf{x})dx\right] = 0 \tag{S47}$$

$$\mathbb{E}\left[\left(\int \psi(\mathbf{x})dx\right)^2\right] = \int C(x_1, x_2)dx_1dx_2 + \sigma_{\psi_0}^2\left(\int dx\right)^2 \tag{S48}$$

$$\mathbb{E}\left[\int \psi^2(x)dx\right] = \int C(x,x)dx + \sigma_{\psi_0}^2 \int dx. \tag{S49}$$

We define the mean $\bar{\psi} = \int \psi(\mathbf{x})dx / \int dx$. From the properties above, $\mathbb{E}[\bar{\psi}] = 0$. We also define the overall variance, which is the expectation of the normalized and squared deviation of $\psi(\mathbf{x})$ from the ensemble mean,

$$V_1'' = \mathbb{E}\left[\frac{\int (\psi(\mathbf{x}) - \mathbb{E}[\bar{\psi}])^2 dx}{\int dx}\right] = \frac{\int C(x,x)dx}{\int dx} + \sigma_{\psi_0}^2 = \sigma_{\psi}^2 + \sigma_{\psi_0}^2, \tag{S50}$$

and the inter-particle variance, which is the variance of the mean,

$$V_3'' = \mathbb{V}\mathrm{ar}[\bar{\psi}] = \mathbb{E}\left[\left(\frac{\int \psi(\mathbf{x})dx}{\int dx}\right)^2\right] = \frac{\int C(x_1,x_2)dx_1dx_2}{\int dx_1dx_2} + \sigma_{\psi_0}^2. \tag{S51}$$

Given the correlation function, when l is much smaller than the size of the domain, in 2D, we have

$$V_3'' \approx \frac{2\pi l^2}{A}\sigma_{\psi}^2 + \sigma_{\psi_0}^2, \tag{S52}$$

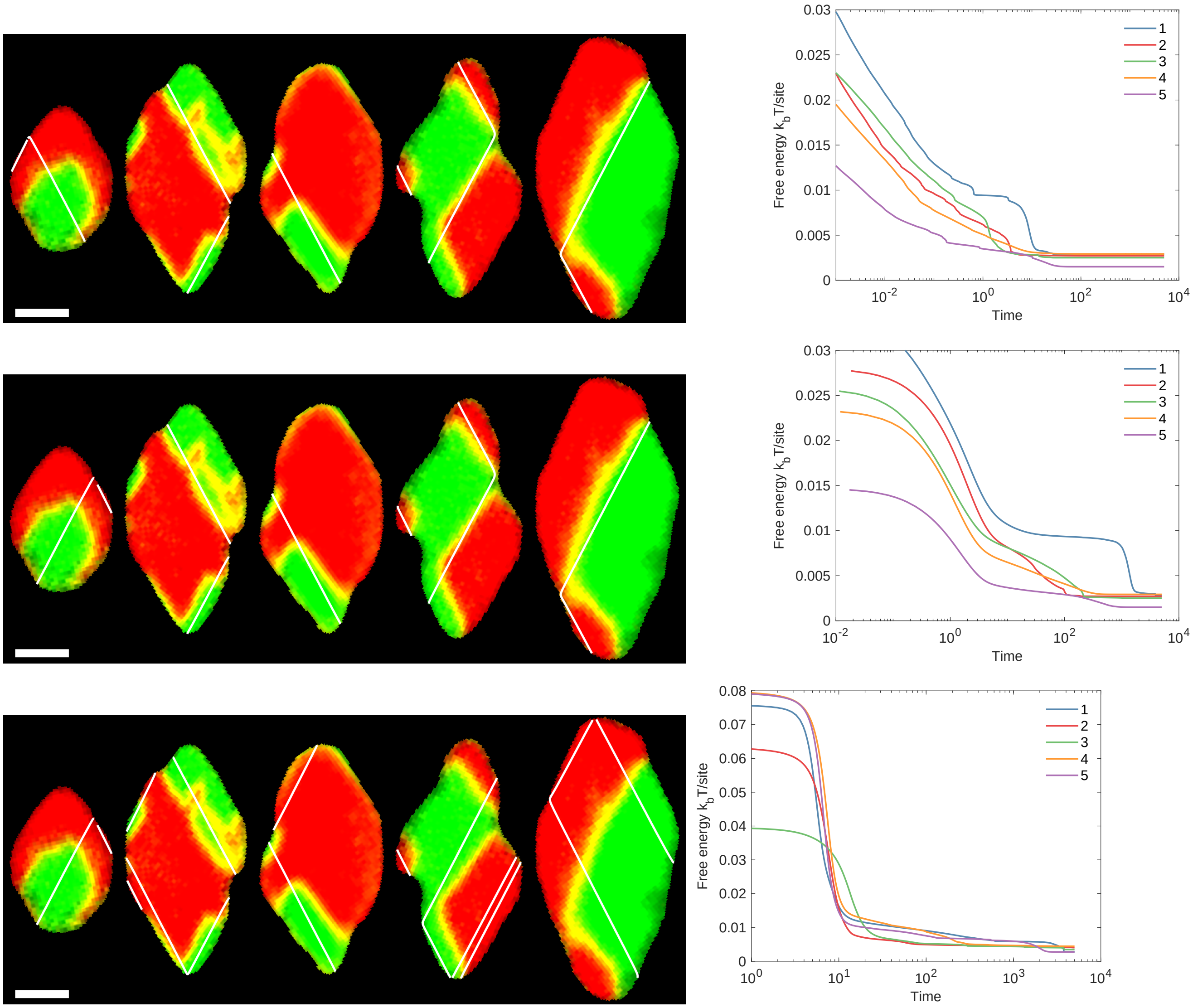

Figure S9: Similar to Fig. S5. The LFP-FP interface from the simulation at the last time step is shown on the experimental image as the white curve for comparison. From top to bottom, simulations correspond to relaxation via diffusion, via reaction (starting from experimental images), and relaxation via reaction (starting from a uniform concentration field). Legend names 1 to 5 in the free energy curves correspond to particles from left to right. The scale bar is 500 nm.

where A is the area of the domain. In our study, the correlation length is chosen to be the size of the pixel, which is much smaller than the particle size ($l^2 \ll A$), hence the inter-particle variance is approximately $V_3'' \approx \sigma^2_{\psi_0}$.

Finally, we define the intra-particle variance, which is the expectation of the normalized and squared deviation of $\psi(\mathbf{x})$ from the particle mean,

$$V_2'' = \mathbb{E}\left[\frac{\int \left(\psi(\mathbf{x}) - \bar{\psi}\right)^2 dx}{\int dx}\right] = V_1'' - V_3''. \tag{S53}$$

Given the approximation for the inter-particle variance, the intra-particle is approximately $V_2'' \approx \sigma^2_{\psi}$.

We often need to quantify the variance of a sample of particles. Here we define the sample overall, intra-particle, and inter-particle variance by area (all pixels have the same weight),

$$\begin{aligned} V_1 &= \frac{\sum_i \int \left(\psi_i(x) - \bar{\bar{\psi}}\right)^2 dx}{\sum_i A_i} \\ V_2 &= \frac{\sum_i \int \left(\psi_i(x) - \bar{\psi}_i\right)^2 dx}{\sum_i A_i} \\ V_3 &= \frac{\sum_i A_i \left(\bar{\psi}_i - \bar{\bar{\psi}}\right)^2}{\sum_i A_i}, \end{aligned} \tag{S54}$$

where $\psi_i(x)$ is $\ln k(\mathbf{x})$ of the ith particle, $\bar{\psi}_i$ is the mean of the ith particle, A_i is the area of the ith particle, $i = 1, \dots, N$, and $\bar{\bar{\psi}}$ is the overall mean of all particles by area, i.e.,

$$\bar{\bar{\psi}} = \frac{\sum_i \int \psi_i(x) dx}{\sum_i A_i}. \tag{S55}$$

We can also define the variance by particle (all particles are given the same weight),

$$V_1' = \frac{1}{N} \sum_i \frac{\int \left(\psi_i(x) - \bar{\bar{\psi}}'\right)^2 dx}{A_i} \tag{S56}$$

$$V_2' = \frac{1}{N} \sum_i \frac{\int \left(\psi_i(x) - \bar{\psi}_i\right)^2 dx}{A_i} \tag{S57}$$

$$V_3' = \frac{1}{N} \sum_i \left(\bar{\psi}_i - \bar{\bar{\psi}}'\right)^2, \tag{S58}$$

where $\bar{\bar{\psi}}'$ is the overall mean by particle,

$$\bar{\bar{\psi}}' = \frac{1}{N} \sum_i \bar{\psi}_i. \tag{S59}$$

V_1 and V_1' can be expanded and written as

$$V_1 = \frac{\sum_i \int (\psi_i(x))^2 dx}{\sum_i A_i} - 2\bar{\bar{\psi}} \frac{\sum_i \int \psi_i(x) dx}{\sum_i A_i} + \bar{\bar{\psi}}^2 = \frac{\sum_i \int (\psi_i(x))^2 dx}{\sum_i A_i} - \bar{\bar{\psi}}^2, \tag{S60}$$

and

$$V_1' = \frac{1}{N} \sum_i \frac{\int (\psi_i(x))^2 dx}{A_i} - 2\bar{\bar{\psi}}' \cdot \frac{1}{N} \sum_i \frac{\int \psi_i(x) dx}{A_i} + \bar{\bar{\psi}}'^2 = \frac{1}{N} \sum_i \frac{\int (\psi_i(x))^2 dx}{A_i} - \bar{\bar{\psi}}'^2. \tag{S61}$$

Similarly,

$$V_3 = \frac{\sum_i A_i \bar{\psi}_i^2}{\sum_i A_i} - \bar{\bar{\psi}}^2, \tag{S62}$$

$$V_3' = \frac{1}{N} \sum_i \bar{\psi}_i^2 - \bar{\bar{\psi}}'^2, \tag{S63}$$

$V_2 = V_1 - V_3$, and $V_2' = V_1' - V_3'$.

Because k is a multiplicative factor and both k and $j_0(c)$ are inferred from the images, it is necessary to impose a normalization constraint, which can either be $\bar{\bar{\psi}} = 0$ or $\bar{\bar{\psi}}' = 0$. We use the former in this study. Suppose the sample $\psi_i(x)$ follows the distribution $\psi_i(x) \sim \psi(\mathbf{x})$ i.i.d. for $i = 1, 2, \ldots$. When the constraint $\bar{\bar{\psi}} = 0$ is imposed, we have $E[V_1] = V_1''$, and in the limit of $l^2 \ll A$, we have

$$\mathbb{E}[V_3] \approx \sigma_{\psi_0}^0 \approx V_3'', \tag{S64}$$

and $\mathbb{E}[V_2] \approx V_2''$. Similarly, when the constraint $\bar{\bar{\psi}} = 0'$ is imposed, $E[V_1'] = V_1''$, and when $l^2 \ll A$, $E[V_2'] = V_2''$ and $E[V_3'] = V_3''$.

In summary, when the correlation length is much smaller than the particle size, the sample intra-particle variance V_2 and V_2' are unbiased estimators of the intra-particle variance $\sigma_{\psi_0}^2$. When the constraint $\bar{\bar{\psi}} = 0$ ($\bar{\bar{\psi}}' = 0$) is imposed, the sample inter-particle variance V_3 (V_3') is a unbiased estimator of the inter-particle variance $\sigma_{\psi_0}^2$. Regardless of the particle size, when the constraint $\bar{\bar{\psi}} = 0$ ($\bar{\bar{\psi}}' = 0$) is imposed, the overall sample variance V_1 (V_1') is a unbiased estimator of the overall variance $\sigma_{\psi}^2 + \sigma_{\psi_0}^2$. We use this property later to estimate σ_{ψ} and σ_{ψ_0}.

Given the prior structure, we expand $\psi(\mathbf{x}) - \psi_0$ in orthogonal basis functions, that is, we use the Karhunen-Loeve expansion to represent $\psi(\mathbf{x})$ using eigenfunctions of the covariance operator,

$$\int C(x_1 - x_2)\phi_i(x_2)dx_2 = \lambda_i \phi_i(x_1). \tag{S65}$$

We use finite elements to numerically solve the eigenvalue problem by taking its inner product with finite element basis $\{\phi_{bi}(x)\}$. This procedure converts the integral equation to a matrix form. Given that each eigenfunction is $\phi(x) = \sum_j s_j \phi_{bj}(x)$,

$$\sum_j s_j \iint C(x_1 - x_2)\phi_{bi}(x_1)\phi_{bj}(x_2)dx_1 dx_2 = \lambda_i \sum_j s_j \int \phi_{bi}(x_1)\phi_{bj}(x_1)dx_1. \tag{S66}$$

which is a generalized eigenvalue problem of the form $Ax = \lambda_k Bx$. The integrals are computed using quadrature. After obtaining the eigenfunctions, $\psi(\mathbf{x})$ can be written as

$$\psi(\mathbf{x}) = \sigma_{\psi_0} Z_0 + \sum_{i=1}^{N_{\mathrm{KL}}} \sqrt{\lambda_i}\phi_i(x) Z_i, \tag{S67}$$

where $Z_i \sim \mathcal{N}(0, 1)$ ($i = 0, \ldots, N_{\mathrm{KL}}$) i.i.d.. Oftentimes, we need to numerically compute the variance of $\psi(\mathbf{x})$ given $\mathbf{Z}$. Recall that $\psi_i(x)$ are orthonormal,

$$\int \psi^2(x)dx = \sum_{i=1}^{N_{\mathrm{KL}}} \lambda_i Z_i^2 + 2\sum_{i=1}^{N_{\mathrm{KL}}} \sigma_{\psi_0} Z_0 Z_i \int \sqrt{\lambda_i}\phi_i(x)dx + \sigma_{\psi_0}^2 Z_0^2 \cdot \int dx. \tag{S68}$$

4.2 Estimates of intra- and inter-particle variance

When the concentration field $c(x)$ is uniform and the insertion or de-insertion reaction occurs, from Eq. S45 and with $\alpha = 0.5$,

$$\frac{\partial c}{\partial t} = k(\mathbf{x}) j_0(c) \cdot 2\sinh\frac{\mu_{\mathrm{res}} - \mu}{2} = k(\mathbf{x}) K_I, \tag{S69}$$

where K_I is a constant in space. Therefore we can estimate $\psi(\mathbf{x}) = \ln k(\mathbf{x})$ using

$$\psi(\mathbf{x}) - \bar{\psi} = \ln\left(K_I^{-1}\frac{\partial c}{\partial t}\right) - \overline{\ln\left(K_I^{-1}\frac{\partial c}{\partial t}\right)}, \tag{S70}$$

where the overhead bar is the spatial average over the particle. This quantity is important in finding the intra-particle variance. When $k(\mathbf{x})$ is close to 1, the expression above is approximately

$$\psi(\mathbf{x}) - \bar{\psi} \approx \frac{\frac{\partial c}{\partial t}}{\overline{\frac{\partial c}{\partial t}}} - 1. \tag{S71}$$

Proof: For brevity, we use k to represent $k(\mathbf{x})$ First, we have

$$\ln k = \ln \bar{k} + \ln\frac{k}{\bar{k}} \approx \ln \bar{k} + \frac{k}{\bar{k}} - 1 \tag{S72}$$

therefore

$$\overline{\ln k} \approx \ln \bar{k} \tag{S73}$$

Hence the left-hand side of the equation is

$$\psi(\mathbf{x}) - \bar{\psi} = \ln k - \overline{\ln k} \approx \ln\frac{k}{\bar{k}} \approx \frac{k}{\bar{k}} - 1 \tag{S74}$$

Since K_I is a constant in space, we have

$$\frac{k}{\bar{k}} = \frac{\frac{\partial c}{\partial t}}{\overline{\frac{\partial c}{\partial t}}} \tag{S75}$$

From Section 4.1, we use the sample variation V_2 to estimate intra-particle variance $\sigma^2_{\psi_0}$. The time derivative $\frac{\partial c}{\partial t}$ used to estimate $\psi(\mathbf{x})$ is estimated using finite difference of two consecutive frames. The frames chosen must satisfy the following conditions: the concentration field is close to being uniform with no phase separation and, during Li insertion, the frames chosen must not contain pixels whose Li fraction is too close to 1, because those regions are prevented from reacting further by the thermodynamic limit (hence the overpotential is nonuniform and K_I is not constant in space) and do not reflect the kinetic properties and will be misidentified as the kinetically slow region; similarly, during Li de-insertion, the Li fraction must not be too close to 0. The difference in the average compositions of the two frames $\overline{\Delta c}$ used for finite differencing is a subtle choice. It should not be too large due to the error of finite differencing compared to the time derivative. It should also not be too small because the pixel noise leads to an increase in the estimated intra-particle variance by $2\sigma^2_\epsilon/\overline{\Delta c}$.

In practice, we use the following set of criteria to determine pairs eligible for finite differencing:

- The smaller concentration variance ($\int(c(x) - \bar{c})^2dx/\int dx$) of the two frames is smaller than 0.01.
- The larger concentration variance of the two frames is smaller than 0.02.
- The difference in the average Li fraction between the two frames is greater than 0.05 and smaller than 0.3
- For Li insertion episodes, the percentage of pixels whose Li fraction is above 0.85 in the selected frame is smaller than 1%. For Li extraction episodes, the percentage of pixels whose Li fraction is below 0.15 in the selected frame is smaller than 1%.

For some particles with multiple pairs of consecutive frames that satisfy the criteria above, we take the average of the estimated $\psi(\mathbf{x}) - \bar{\psi}$ based on these multiple pairs.

The finite differencing estimates based on pairs from the same episode are averaged to obtain an estimate for $\psi(\mathbf{x})$. $\psi(\mathbf{x}) - \bar{\psi}$ from all available episodes of the same particle are averaged to give an estimate for σ_ψ. The overall σ_ψ is computed by taking the area-weighted average for all available particles (V_2). Both the logarithm method (Eq.

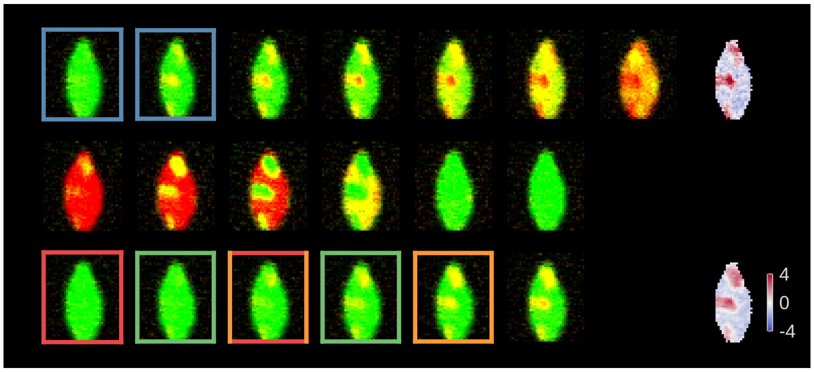

Figure S10: An example of particle for which estimates of the spatial heterogeneity $\psi(\mathbf{x}) - \bar{\psi}$ are performed. On the left are images of lithium fraction from STXM. Each row corresponds to an episode. Frames with the same color are pairs that are used in finite differencing. Some frames have two colors, meaning that they are used in two different pairs. On the right are the estimated $\psi(\mathbf{x}) - \bar{\psi}$ based on the episode of the same row. The average of $\frac{\frac{\partial c}{\partial t}}{\frac{\partial \bar{c}}{\partial t}} - 1$ of all pairs is used as an estimate for $\psi(\mathbf{x}) - \bar{\psi}$ of each episode, respectively. Pixels whose values are smaller than -1 are rare and have a local reaction rate that has an opposite sign compared to that of the average reaction rate. $\psi(\mathbf{x}) - \bar{\psi}$ maps share the same color map.

S70) and ratio method (Eq. S71) are used. When the logarithm method is used, pixels at which $K_I^{-1}\frac{\partial c}{\partial t}$ is negative are omitted. The estimate for σ_ψ using the logarithm and ratio methods are 0.68 and 0.76, respectively.

Fig. S10 shows an example of the pairs used for finite differencing and the estimated $\ln k(\mathbf{x})$ $(\frac{\frac{\partial c}{\partial t}}{\frac{\partial \bar{c}}{\partial t}} - 1)$. The estimated spatial heterogeneity from the first and third episodes shows a correlation coefficient of 0.81.

For the estimation of inter-particle variance, we select particles in the same batch of experiments having similar average composition at the same time and calculate the average reaction rate of each particle R_i. Assuming that all particles have the same composition and overpotential, the variance of $\ln R_i$ is an estimate of V_3. However, because the local environment of particles is different, the overpotential can be different, hence this approach may overestimate V_3. Fig. S11 shows the trajectory of the average compositions of all particles in the same batch of experiments (with the same global charge/discharge rate and belonging to the same electrochemical cell). The region of data selected to calculate V_3 is highlighted by rectangles. V_3 for the different batches shown in Fig. S11 are 1.51, 0.92, 0.25, 1.11, 0.33, and 0.76, respectively.

5 Inference based on different objective functions

In this section, we consider different objective functions that can be used to infer the unknown quantities in the model, $g_h(c)$, $j_0(c)$, and $k(\mathbf{x})$, including metrics that describe the uniformity of the concentration field, compared with using full-image pixel-to-pixel error.

5.1 Inference based on uniformity coefficient

Ref. [1] observed that the uniformity of the Li concentration field $c(x)$ depends on the C-rate (reaction rate) and direction of reaction (charge or discharge). As mentioned in the main text, it is possible to infer the reaction kinetics and thermodynamics from the uniformity coefficient. Here we discuss in detail the model and procedure.

The heterogeneity of the concentration field is defined by its variance $v(t) = \int (c(x,t) - \bar{c}(t))^2 dx / \int dx$. The maximum variance $(\bar{c}(1-\bar{c}))$ given the mean $\bar{c}$ is attained when the concentration values are either 0 or 1. The closer $v(t)$ is to this maximum value, the more nonuniform the concentration field. Following Lim et al. [1], we

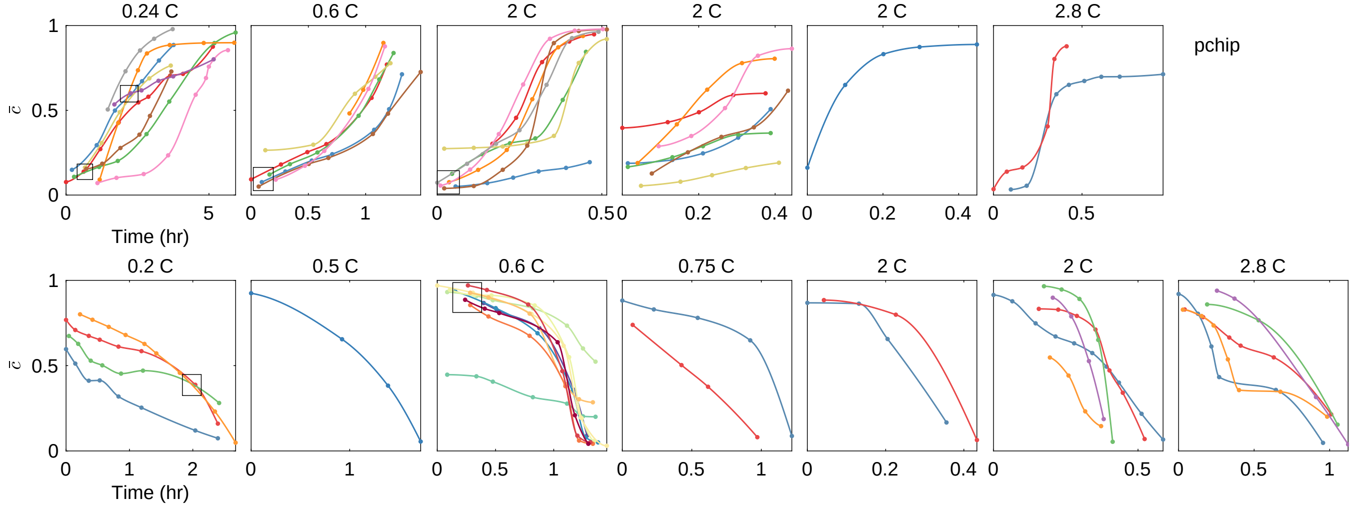

Figure S11: The average composition of particles based on images taken versus time since the beginning of discharge (the first row) or charge (the second row). The title of each plot is the global C-rate. The discrete data points are interpolated using pchip. The reaction rates in the highlighted boxes are used to compute interparticle variation.

define the uniformity coefficient UC from the least squares fitting of the standard deviation according to $\sqrt{v(t)} = (1 - \mathrm{UC})\sqrt{\bar{c}(t)(1 - \bar{c}(t))}$, that is, given $v(t_i)$ at each time point of the episode,

$$\mathrm{UC} = 1 - \frac{\sum_i \sqrt{v(t_i)\bar{c}(t_i)(1 - \bar{c}(t_i))}}{\sum \bar{c}(t_i)(1 - \bar{c}(t_i))}. \tag{S76}$$

The model predicted UC is computed using $c(x, t)$ from simulations at a fine time resolution (100 time points from beginning to end). Using Bayesian inference, the likelihood is

$$p(\mathrm{UC}_{\mathrm{data}}|\mathbf{p}) \propto \exp - \sum_i \frac{(\mathrm{UC}(I_i; \mathbf{p}) - \mathrm{UC}_{\mathrm{data}}(I_i))^2}{2\sigma^2_{\mathrm{UC},i}}, \tag{S77}$$

where UC and $\mathrm{UC}_{\mathrm{data}}$ refer to the model predicted and experimental uniformity coefficients, respectively, I_i is the global C-rate of the ith data point, $\sigma^2_{\mathrm{UC},i}$ is the variance of the ith data point measured experimentally, $\mathbf{p}$ are the model parameters. The model predicted UC is solved at constant C-rate I_i.The data can be found in Fig. 3E of Ref. [1]. The data point that corresponds to $I_i = 0$ is not used in the fitting, since the model prediction for UC at equilibrium does not dependent on the parameters related to reaction kinetics.

Since the model predicted UC is for any generic particle geometry, the model is solved on a square domain with periodic boundary condition. The surface heterogeneity is assumed to be a realization of a Gaussian random field, where the covariance is given in Eq. S46. With periodic boundary condition, the eigenfunctions of the convolution operator are Fourier basis functions, in other words,

$$P[\psi(\mathbf{x})] \propto \exp\left[-\frac{1}{2}\int \psi(x_1)\Lambda(x_1 - x_2)\psi(x_2)dx_1dx_2\right] = \exp\left[-\frac{1}{2}\sum_k \hat{\Lambda}_k|\hat{\psi}_k|^2\right], \tag{S78}$$

where $\Lambda(x_1 - x_2)$ is the inverse covariance, and hat is used to denote Fourier transform, $\hat{\Lambda}_k = \hat{C}_k^{-1}$. Therefore, the random field $\psi(\mathbf{x})$ can be generated by first generating a white noise random field (no spatial correlation) $\xi(x)$, and via $\hat{\psi}_k = \hat{\Lambda}_k^{-1/2}\hat{\xi}_k$. The variance of $\psi(\mathbf{x})$ (σ^2_ψ) is an unknown parameter and we assume its prior is $\ln \sigma_\psi \sim \mathcal{N}(\ln 0.1, 2)$.

Due to the limited number of data points for UC (6), we use a one-parameter representation of $\mu_h(c)$ constrained by the mosaic miscibility gap (Eq. S30). We know that, in order for $c = 0.5$ to be within the spinodal region, we

have $\mu_h'(c = 0.5) < 0$. Since, with this representation, $\mu_h'(c = 0.5) = 4 - 2\Omega - p_{\mathrm{GL}}\mu_{\mathrm{GL}}'(c = 0.5)$, we choose a reasonable prior for $p_{\mathrm{GL}} \sim \mathcal{N}(0, |p_{\mathrm{GL,crit}}|)$ where $p_{\mathrm{GL,crit}} = (4 - 2\Omega)/\mu_{\mathrm{GL}}'(c = 0.5)$. Alternatively, we also consider representing $\mu_h(c)$ using Eq. S31 and require $p_{\mathrm{GL}} > 0$ for stability. Based on the literature value for the spinodal barrier, we choose a reasonable prior $\ln p_{\mathrm{GL}} \sim \mathcal{N}(0, 0.5)$.

It is known that, at higher overpotential, the dependence of the reaction rate on overpotential can deviate from exponential dependence as predicted by Butler-Volmer. This deviation can be due to Ohmic resistance or electron transfer rate at high potential (Markus-Hush-Chidsey model [19, 29]) both of which cause $d\ln R/d\eta$ to decrease at high overpotential. Therefore, we examine the importance of the overpotential dependence given the experimentally measured uniformity coefficient in this section. For the reaction kinetics R, we consider the effect of film resistance that is in series with the surface reaction that follows Butler-Volmer kinetics,

$$R = j_0(c)\left[e^{(\tilde{\eta}-RR_\Omega)/2} - e^{-(\tilde{\eta}-RR_\Omega)/2}\right], \tag{S79}$$

where R_Ω is the film resistance scaled by the thermal voltage. The film resistance is an unknown parameter and we assume its prior follows $\ln R_\Omega \sim \mathcal{N}(\ln 0.1, 2)$. The surface heterogeneity is multiplicative, $\frac{\partial c}{\partial t} = k(\mathbf{x})R$.

In the literature, a symmetric exchange current $\sqrt{c(1-c)}$ is frequently used [72]. From transition state theory [7], we expect that the exchange current approaches zero as c approaches 0 and 1. Hence we use the representation in this section,

$$j_0(c) = K\left[\left(\frac{c}{c_m}\right)^{c_m}\left(\frac{1-c}{1-c_m}\right)^{1-c_m}\right]^{\gamma}, \tag{S80}$$

where γ sets the shape, $K = \max_c j_0(c)$, and $c_m = \operatorname{argmax}_c j_0(c)$ since

$$\frac{\partial \ln j_0(c)}{\partial c} = \gamma\left[\frac{c_m}{c} - \frac{1-c_m}{1-c}\right]. \tag{S81}$$

and $\partial \ln j_0(c_m)/\partial c = 0$. We assume the prior for c_m follows uniform distribution on $(0, 1)$. We use the transformation $z_m = \ln\frac{c_m}{1-c_m} \in \mathbb{R}$. Therefore the probability distribution of the prior for z_m is

$$P(z_m) = \frac{dc_m}{dz_m} = \frac{1}{4}\operatorname{sech}^2\frac{z_m}{2}. \tag{S82}$$

K and γ are positive, and we assume their priors are given by $\ln K \sim \mathcal{N}(\ln 0.5, 2)$ (the unit of K is hr^{-1}) and $\ln\gamma \sim \mathcal{N}(\ln 1.5, 2)$. In total, we have 6 parameters $\mathbf{p} = [K, c_m, \gamma, p_{\mathrm{GL}}, \sigma_\psi, R_\Omega]$ whose priors are independent.

The initial condition $c(x)$ is generated using $\ln\frac{c(x)}{1-c(x)} = \mu' + \sigma_0'\psi(\mathbf{x})$, where μ' and σ_0' is determined such that $\bar{c}(t = 0) = \int c(x, t = 0)dx/\int dx$ is 0.05 and 0.95 for lithiation and delithiation, respectively, and that the variance $\sigma_{c_0}^2 = \int (c(x, t = 0) - \bar{c})^2 dx/\int dx$ is 0.05^2, as suggested by the experimental error.

To illustrate the importance of reaction kinetics, we first omit the mechanical effects in the model. In a large domain, the effective gradient penalty scaled by the domain size $\kappa/L^2 \to 0$, and the correlation length of the surface heterogeneity field $\psi(\mathbf{x})$ satisfies $\sqrt{\kappa} \ll l \ll L$, the continuum model reduces to the discrete 0D model, in which all grid points (pixels) has no spatial interaction. We find the maximum a posteriori estimate (MAP) for $\mathbf{p}$ using the discrete model (Eq. S31 is used to represent the chemical potential), and then solve both discrete and continuum model ($\sqrt{\kappa} = 0.01L$, $l = 0.2L$) using MAP $\mathbf{p}$. The simulations are repeated 10 times with different random noise (for the initial condition and surface heterogeneity) to obtain the mean and variance of the model-predicted UC.

Fig. S12a shows that both models are sufficiently close to the experimental values. The insets show examples of the concentration fields at $\bar{c} = 0.5$ at the same current and opposite directions, illustrating the increasing uniformity with increasing C-rate and the asymmetry between lithiation and delithiation.

When mechanics is included in the model, we must use the full continuum model to find the MAP. In Fig. S12b, the uncertainty of the MAP model prediction due to stochasticity is well within the uncertainty in the UC data. The influence of C-rate and direction on the uniformity coefficient and the concentration field is qualitatively similar to the model that omits mechanics.

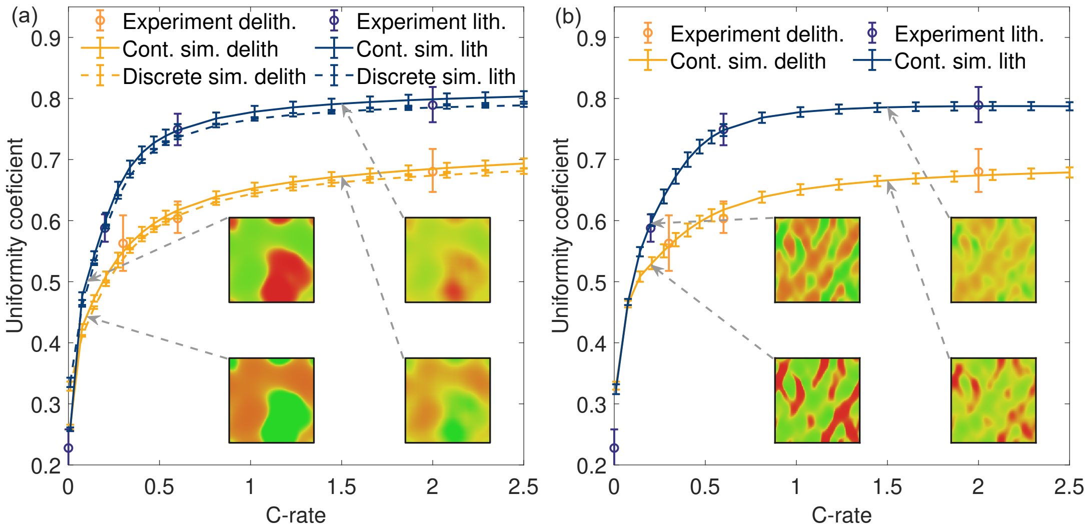

Figure S12: The comparison between uniformity coefficients predicted by the discrete model (Cont. sim.), continuum model (Cont. sim.), and the experimental values (Experiment). The error bars for the model prediction are the standard deviation from 10 realizations of simulations. (a) the continuum model does not have coherency strain.(b) the continuum model with coherency strain is fitted to the uniformity coefficient data. The inset figures are the simulated concentration fields at an average fraction of 50% at C-rates indicated by the arrows. For the inset figures in (b), the horizontal and vertical axes are a and c directions of the LFP crystal lattice, respectively.

Next, based on the full continuum model with mechanics, we perform Hamiltonian Monte Carlo (HMC) to sample the posterior distribution of the parameters. The number of steps is 5; the step size is 0.1. 5000 samples are used, with an acceptance ratio of 0.81. FSA is used to compute the gradient of the log-posterior. The scatter plots and histograms for each pair and each parameter are shown in the left panel of Fig. S13. Instead of p_{GL}, we plot $\mu_h'(c = 0.5)$. We see a strong correlation between K and $\mu_h'(c = 0.5)$. The two parameters for the shape of $j_0(c)$ is also highly correlated. We attempt to understand this correlation analytically.

Without the voltage and spatial information, the evolution of the concentration variance is approximated by the auto-catalytic rate s defined below to first order [53],

$$s = \frac{dR}{dc} = \frac{\partial R}{\partial c} + \frac{\partial R}{\partial \mu}\frac{d\mu}{dc}. \tag{S83}$$

Normalizing s by the reaction rate,

$$\frac{s}{R} = \frac{d \ln R}{dc} + \frac{\partial \ln R}{\partial \mu}\frac{d\mu}{dc}. \tag{S84}$$

In the case of Butler-Volmer kinetics,

$$\frac{s}{R} = \frac{d \ln j_0}{dc} + \frac{1}{R}\frac{d\mu}{dc}\sqrt{\left(\frac{R}{2}\right)^2 + j_0^2(c)}; \tag{S85}$$

at low rate ($R \longrightarrow 0$),

$$\frac{s}{R} \approx \frac{d \ln j_0}{dc} + \frac{j_0}{R}\frac{d\mu}{dc}, \tag{S86}$$

and at high rate ($R \longrightarrow \pm\infty$),

$$\frac{s}{R} \approx \frac{d \ln j_0}{dc} + \frac{\mathrm{sgn}(R)}{2}\frac{d\mu}{dc}. \tag{S87}$$

At lower C-rates, the compositional heterogeneity is determined more by the thermodynamics (the magnitude of the second term in Eq. S86 is large compared to the first), and less by the reaction kinetics, that is, $s/R \sim j_0 R^{-1} \cdot d\mu/dc$.

Therefore, given UC in the low rate limit, the posterior falls on the manifold of constant s/R, or $1/j_0 \propto d\mu/dc$ (note that the proportionality constant is negative because $j_0 > 0$ and $d\mu/dc < 0$ to ensure thermodynamic phase separation). Since $d\mu/dc$ differs from $\mu_h'(c)$ due to the mechanical effect we perform a linear regression between $1/K$ and $\mu_h'(c = 0.5)$ (it is found a regression between $1/j_0(c = 0.5)$ and $\mu_h'(c = 0.5)$ gives similar results) and found

$$\frac{d\mu_h}{dc}(c = 0.5) = -\frac{1.2 \text{ hr}^{-1}}{K} - 5.1. \tag{S88}$$

The fitted correlation between K and $\mu_h'(c = 0.5)$ is shown by the red curves in the scattered plots of Fig. S13

Since the experimental UC is observed at multiple C-rates, we expect that the first and second terms of the autocatalytic rate Eq. S85 can be identified separately since the C-rate affects only the second term and not the first term. Therefore, we hypothesize that in the posterior distribution, c_m and γ approximately lie on the manifold of constant $(\ln j_0)'$. Since

$$\frac{d \ln j_0}{dc} = \gamma\left(\frac{c_m}{c} - \frac{1 - c_m}{1 - c}\right) = \gamma\frac{c_m - c}{c(1 - c)}, \tag{S89}$$

we perform a linear regression between c_m and $1/\gamma$ (shown by the green curves in the scattered plots of Fig. S13) to find that

$$-\frac{0.39}{\gamma} = c_m - 0.57, \tag{S90}$$

which translates into $\ln j_0'(c = 0.57) \approx -1.57$. Having collapsed c_m and γ into a single parameter $\ln j_0'(c = 0.57)$ related by the first term in Eq. S85, and K and $\mu_h'(c = 0.5)$ into a single parameter related by the second term in Eq. S85, these two pairs can be identified separately. This is confirmed by the lack of any significant correlation between the pairs (the shape parameters of $j_0(c)$ are not strongly correlated with the magnitude of $j_0(c)$ and the thermodynamic parameter p_{GL}). In fact, we have shown previously [31] that datasets from both lithiation and lithiation allow us to decorrelate $\ln j_0'(c)$ and $\mu_h'(c)$.

The analysis above shows that the magnitude of j_0 cannot be determined from this set of uniformity coefficient data due to its strong correlation with p_{GL}. However, its shape can be determined separately using effectively one parameter. We show the uncertainty of $j_0(c)/K$ in red in the right panel of Fig. S13, which is defined by the 90% percentile at each c.

Additionally, note that there is some negative correlation between the strength of surface heterogeneity σ_ψ and the chemical potential barrier, and some positive correlation between σ_ψ and K, both of which suggest that when the system has a greater tendency to phase separate, the surface heterogeneity should be smaller given the same observed values of uniformity coefficient. We also find that the film resistance R_Ω is not sensitive to the observed UC and does not have any significant correlation with other parameters. Therefore, the following discussion omits the film resistance R_Ω.

In comparison, we perform HMC using the discrete model without mechanical effects and with Eq. S31) (10000 samples and the acceptance ratio is 0.85). We find a strong correlation between $\ln K$ and $\ln p_{\text{GL}}$. A linear regression finds

$$p_{\text{GL}} = \frac{2.7 \text{ hr}^{-1}}{K}, \tag{S91}$$

A linear regression also finds a similar correlation between c_m and γ,

$$-\frac{0.31}{\gamma} = c_m - 0.54, \tag{S92}$$

, or $\ln j_0'(c = 0.54) \approx -1.26$. Note that this result is very close to that using the continuum model. In fact, in the right panel of Fig. S13 we plot the uncertainty of $j_0(c)/K$ using the discrete model in gray beneath that for the continuum model and find they are very close. Therefore, the posterior normalized $j_0(c)/j_{0,\text{max}}$ is insensitive to the addition of mechanical effects given the uniformity coefficient data.

Finally, we show the posterior distribution of $j_0(c)$ and $\mu_h(c)$ in Fig. S14 when using full representation Eq. S26 with fixed mosaic and coherent miscibility gap, the same as what will be used in full image inversion, with the number of parameters being $N_\mu = 1$, and $N_J = 3$, less than full image inversion where $N_\mu = 3$, $N_J = 5$.

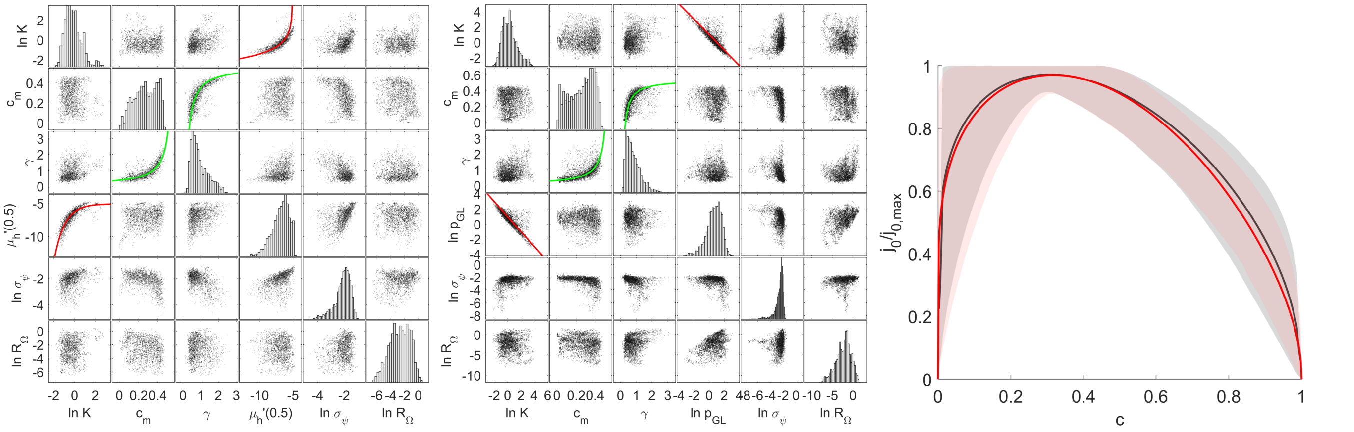

Figure S13: Left and middle: the scatter plots and histograms for each pair and each parameter in $\mathbf{p}$ using the continuum model (5000 samples) and the discrete model (10000 samples), respectively. Red and green lines are fitted curves showing the correlation between K and p_{GL}, as well as c_m and γ, respectively. Right: the mean and 90% confidence interval of $j_0(c)/j_{0,\max}(c) = j_0(c)/K$ using the continuum model (red) and the discrete model (gray).

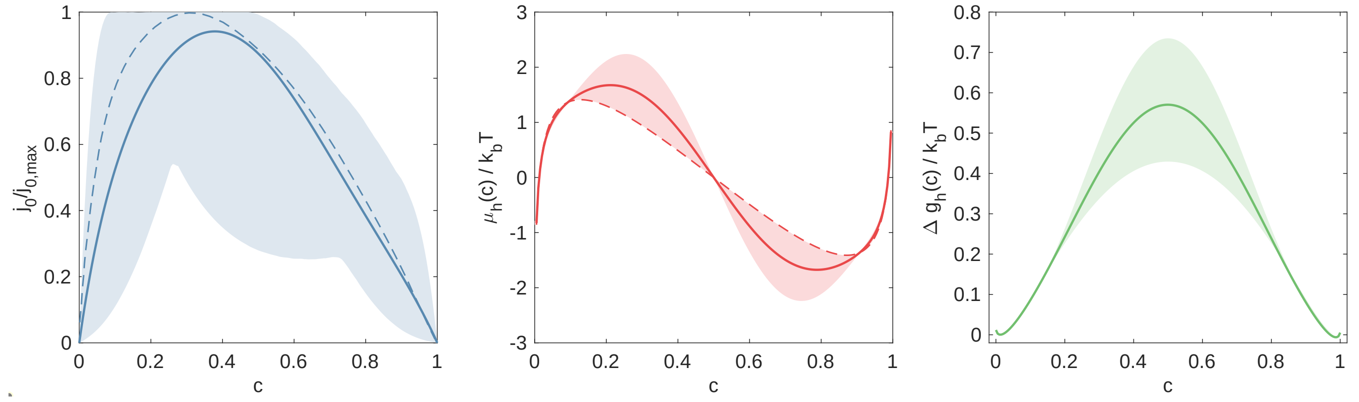

Figure S14: Posterior distributions of $j_0(c)$ and $\mu_h(c)$ given the experimental UC values. The shaded region is the 99% confidence interval.

5.2 Comparison of different objective functions and the number of parameters

In this section, we compare the inference based on 1) all the pixels in the image, 2) the uniformity coefficient, and 3) the variance of the concentration field by evaluating their linear sensitivities with respect to the parameters and through Hamiltonian Monte Carlo sampling of the posterior distributions. We also discuss the number of parameters needed to parameterize $g_h(c)$ and $j_0(c)$.

Suppose the likelihood of the pixel values $c_{\text{data}}(x_i)$ follows $c_{\text{data}}(x_i)-c(x_i;\mathbf{p}) \sim \mathcal{N}(0,\sigma_\epsilon^2)$. Based on the definition of the variance of the concentration field,

$$v_{\text{data}} = \frac{1}{N_p}\sum_{i=1}^{N_p}(c_{\text{data}}(x_i) - \bar{c}_{\text{data}})^2, \tag{S93}$$

where $\bar{c}$ refers to the average composition. Similarly, the model-predicted variance is

$$v(\mathbf{p}) = \frac{1}{N_p}\sum_{i=1}^{N_p}(c(x_i;\mathbf{p}) - \bar{c}_{\text{data}})^2. \tag{S94}$$

Based on the likelihood of the pixel values, the conditional mean and variance of the variance are

$$\mathbb{E}[v_{\text{data}}|\mathbf{p}] = v(\mathbf{p}) + \frac{N_p-1}{N_p}\sigma_\epsilon^2 \tag{S95}$$

$$\mathbb{V}\text{ar}[v_{\text{data}}|\mathbf{p}] = 2\frac{\sigma_\epsilon^2}{N_p}\left(v(\mathbf{p}) + \frac{N_p-1}{N_p}\sigma_\epsilon^2\right) = 2\frac{\sigma_\epsilon^2}{N_p}\mathbb{E}[v_{\text{data}}|\mathbf{p}]. \tag{S96}$$

As an order of magnitude, the value $\sigma_\epsilon^2 = 0.07^2$ is typically smaller than the variance v, we estimate $\mathbb{E}[v_{\text{data}}|\mathbf{p}] \approx v(\mathbf{p})$. Next, given the definition of uniformity coefficient S76, we evaluate the mean and variance of $\sqrt{v_{\text{data}}}$. In the limit of large number of pixels N_p, $\mathbb{V}\text{ar}[v_{\text{data}}|\mathbf{p}]$ is small and $v_{\text{data}}|\mathbf{p}$ approaches a normal distribution. To first-order approximation,

$$\mathbb{E}\left[\sqrt{v_{\text{data}}}|\mathbf{p}\right] \approx \sqrt{\mathbb{E}[v_{\text{data}}|\mathbf{p}]} \approx \sqrt{v(\mathbf{p})} \tag{S97}$$

$$\mathbb{V}\text{ar}\left[\sqrt{v_{\text{data}}}|\mathbf{p}\right] \approx \frac{1}{2}(\mathbb{E}[v_{\text{data}}|\mathbf{p}])^{-1}\mathbb{V}\text{ar}[v_{\text{data}}|\mathbf{p}] \approx \frac{\sigma_\epsilon^2}{N_p}. \tag{S98}$$

Proof: (all symbols used in this proof are local) Given that $x \sim \mathcal{N}(\mu,\sigma^2)$:

$$\begin{aligned}
\mathbb{E}\left[\sqrt{x}\right] &= \frac{1}{\sigma\sqrt{2\pi}}\int \sqrt{x}e^{-\frac{1}{2\sigma^2}(x-\mu)^2} \\
&= \frac{1}{\sigma\sqrt{2\pi}}\int \left(\sqrt{\mu} + \frac{1}{2}\mu^{-1/2}(x-\mu) - \frac{1}{4}\mu^{-3/2}(x-\mu)^2 + o((x-\mu)^2)\right)e^{-\frac{1}{2\sigma^2}(x-\mu)^2} \\
&= \mu^{1/2} - \frac{1}{4}\mu^{-3/2}\sigma^2 + O(\sigma^4)
\end{aligned}$$

and

$$\mathbb{V}\text{ar}\left[\sqrt{x}\right] = \mathbb{E}[x] - \mathbb{E}\left[\sqrt{x}\right]^2 = \mu - \mathbb{E}\left[\sqrt{x}\right]^2 = \frac{1}{2}\mu^{-1}\sigma^2 + O(\sigma^4).$$

Similar to the variance, we define the experimental and model predicted uniformity coefficient UC_{data} and UC, respectively. Given that different snapshots in time are independent and the properties above,

$$\mathbb{E}[\text{UC}_{\text{data}}|\mathbf{p}] \approx \text{UC}(\mathbf{p}) \tag{S99}$$

$$\mathbb{V}\text{ar}[\text{UC}_{\text{data}}|\mathbf{p}] = \frac{\mathbb{V}\text{ar}\left[\sqrt{\hat{v}}\right]}{\sum_i \bar{c}(t_i)(1-\bar{c}(t_i))} = \frac{\mathbb{V}\text{ar}\left[\sqrt{\hat{v}}\right]}{V_{\max}} \approx \frac{\sigma_\epsilon^2}{N_p V_{\max}}, \tag{S100}$$

where we defined $V_{\max}$ as the sum of maximum variance for all snapshots. When the number of pixels is large, the observed UC approaches a normal distribution. Therefore, in summary for each episode,

$$P(\mathbf{c}_{\text{data}}|\mathbf{p}) \propto \exp -\frac{1}{2\sigma_\epsilon^2} \sum_j \sum_{i=1}^{N_p} (c_{\text{data}}(x_i, t_j) - c(x_i, t_j; \mathbf{p}))^2 \tag{S101}$$

$$P(\mathbf{v}_{\text{data}}|\mathbf{p}) \propto \exp -\frac{N_p}{4\sigma_\epsilon^2} \sum_j \frac{(v_{\text{data}}(t_j) - v(t_j; \mathbf{p}))^2}{v(t_j; \mathbf{p})} \tag{S102}$$

$$P(\text{UC}_{\text{data}}|\mathbf{p}) \propto \exp -\frac{N_p V_{\max}}{2\sigma_\epsilon^2} (\text{UC}_{\text{data}} - \text{UC}(\mathbf{p}))^2. \tag{S103}$$

As a result, we compare the sensitivities of the following objective functions to determine the identifiability of the parameters (including multiple episodes at different C-rate I_k):

$$S_{L_2}(\mathbf{p}) = \frac{1}{2N_p} \sum_{j,k} \sum_{i=1}^{N_p} \left((c_{\text{data}}(x_i, t_j, I_k) - c(x_i, t_j, I_k; \mathbf{p})\right)^2 \tag{S104}$$

$$S_v(\mathbf{p}) = \frac{1}{4} \sum_{j,k} \frac{(v_{\text{data}}(t_j, I_k) - v(t_j, I_k; \mathbf{p}))^2}{v(t_j, I_k; \mathbf{p})} \tag{S105}$$

$$S_{\text{UC}}(\mathbf{p}) = \frac{V_{\max}}{2} \sum_k (\text{UC}_{\text{data}}(I_k) - \text{UC}(I_k; \mathbf{p}))^2. \tag{S106}$$

We study the sensitivities by computing the Hessian when the model prediction is equal to the data. For example, the Hessian of the first objective function is

$$H_{L_2} = \frac{1}{N_p} \sum_{j,k} \sum_{i=1}^{N_p} \left(\frac{\partial c_i(t_j, I_k; \mathbf{p})}{\partial \mathbf{p}}\right)^* \frac{\partial c_i(t_j, I_k; \mathbf{p})}{\partial \mathbf{p}}. \tag{S107}$$

We numerically evaluate the Hessian using the model in Section 5.1, with parameters from the optimal result (MAP) from fitting the uniformity coefficients. In this section, we focus on the identifiability of $\mu_h(c)$ and $j_0(c)$, and for simplicity omit the spatial heterogeneity $\sigma_\psi = 0$ and set the series resistance $R_\Omega = 0$. This is equivalent to the best-case scenario with the highest identifiability where the spatial heterogeneity is known. When pixel-based objective function S_{L_2} is used and the spatial heterogeneity is unknown, the uncertainty in $\mu_h(c)$ and $j_0(c)$ increases. Hence, the analysis below provides an upper bound on the sensitivities. The geometry, simulation grid, and parameters are kept the same as that in Section 5.1. We use 5 snapshots for each dataset, which are simulated at C-rates of 0.3, 0.6, and 2 for charge, and 0.2, 0.6, and 2 for discharge. The average concentration ranges from 0.05 to 0.95. Thus, with $V_{\max} = 0.74$, omitting this constant in the objective function (Eq. S106) is acceptable for an order of magnitude estimate. In order to be consistent for all objective functions, the model predicted UC is also defined based on the least square fitting (Eq. S76 given the variance $v(t)$ at the same 5 snapshots, instead of 100 for $v(t)$ used in Section 5.1. The initial condition is generated using the same approach as Section 5.1. Since the initial condition is random, we compute the Hessian given the same initial condition for all C-rates and take the average using 20 realizations.

Using the full representation of $\mu_h(c)$ and $j_0(c)$ ($N_J = N_\mu + 2$), Fig. S15 shows the sorted eigenvalues of the Hessian for the three objective functions using in total 12, 8, and 4 parameters. We find that the eigenvalue declines more rapidly in the order of pixel-, variance-, and UC-based objective functions. Therefore, more information is lost and parameters become increasingly unidentifiable by discarding spatial information and further compressing the experimental data down to statistical descriptors. We provide an estimate of the number of identifiable principal components by the relative magnitude of the eigenvalue compared to the largest. By choosing 10^{-3} as the threshold value, we find that by using S_{UC}, only two principal components can be identified, with the other parameters highly correlated. This is consistent with the conclusion in Section 5.1. With the same threshold, we use a total of

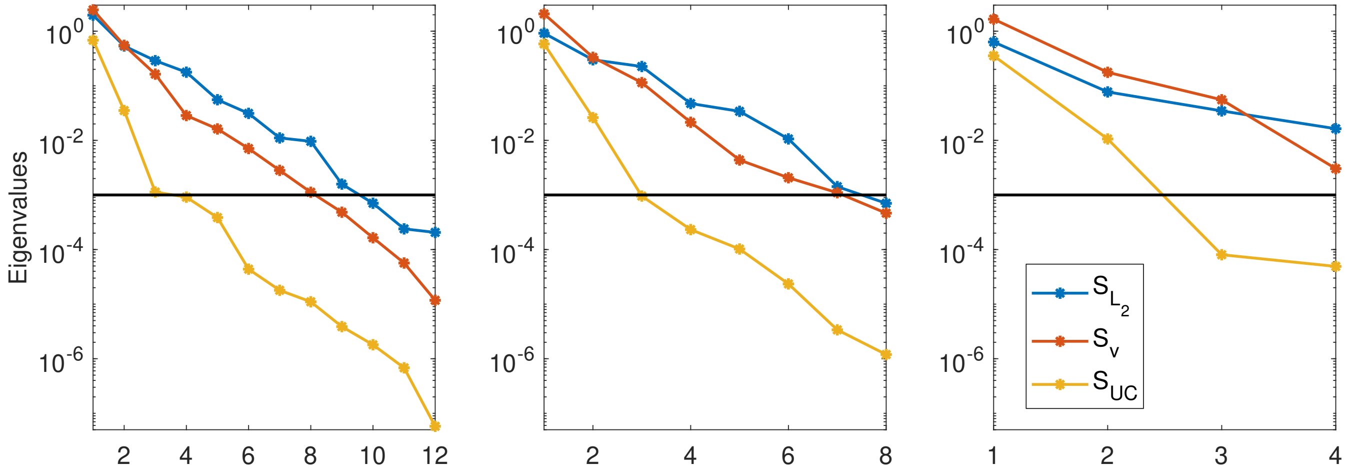

Figure S15: Eigenvalues of the Hessian of different objective functions S_{L_2}, S_v, and S_{UC} using different number of parameters. From left to right, 12, 8, and 4 parameters are used in total. The black dashed lines are the threshold value 10^{-3} used in the main text.

8 parameters ($N_\mu = 3$, $N_J = 3$, that is, $\mu_h(c)$ and $j_0(c)$ are fifth-order polynomials) to parameterize $\mu_h(c)$ and $j_0(c)$ when full concentration field is used as training data.

Fig. S16 compares the posterior distribution of parameters using the two likelihood based on UC (Eq. S103) and pixels (Eq. S101) obtained using Hamiltonian Monte Carlo ($\sigma_\epsilon^2/N_p = 10^{-4}$). We find that using full pixel information significantly reduces the inference uncertainty and the posterior distribution is clustered around the optimum, indicating that using full image data allows us to obtain more accurate inference of the unknown constitutive laws.

6 Optimization and numerical methods

This section discusses the optimization procedure and the numerical methods when full images are used and spatial heterogeneity is included in the inference. The numerical solver for the PDE is the core component of the inversion framework. The requirement on the solver accuracy, its efficiency, and robustness will also be considered in this section.

6.1 Objective function

We define the squared error,

$$\mathrm{SE} = \sum_i \sum_j \| c_{i,j}(\mathbf{p}_{\mathrm{global}}, \mathbf{Z}_i) - c_{\mathrm{data},i,j} \|^2, \tag{S108}$$

where $c_{i,j}$ and $c_{\mathrm{data},i,j}$ are the model prediction and experimental concentration field of the jth episode of particle i and the squared L_2 norm of their difference is defined to be the sum of squared error of all pixels of the episode; the mean squared error

$$\mathrm{MSE} = \frac{\mathrm{SE}}{\sum_i \sum_j N_{p,i}(N_{T,i,j} - 1)}, \tag{S109}$$

where $N_{p,i}$ is the number of pixels of particle i, $N_{T,i,j}$ is the number of frames for the jth episode of particle i, the initial frame is excluded in the normalization because it is used as the initial condition, and the root mean squared error

$$\mathrm{RMSE} = \sqrt{\mathrm{MSE}}. \tag{S110}$$

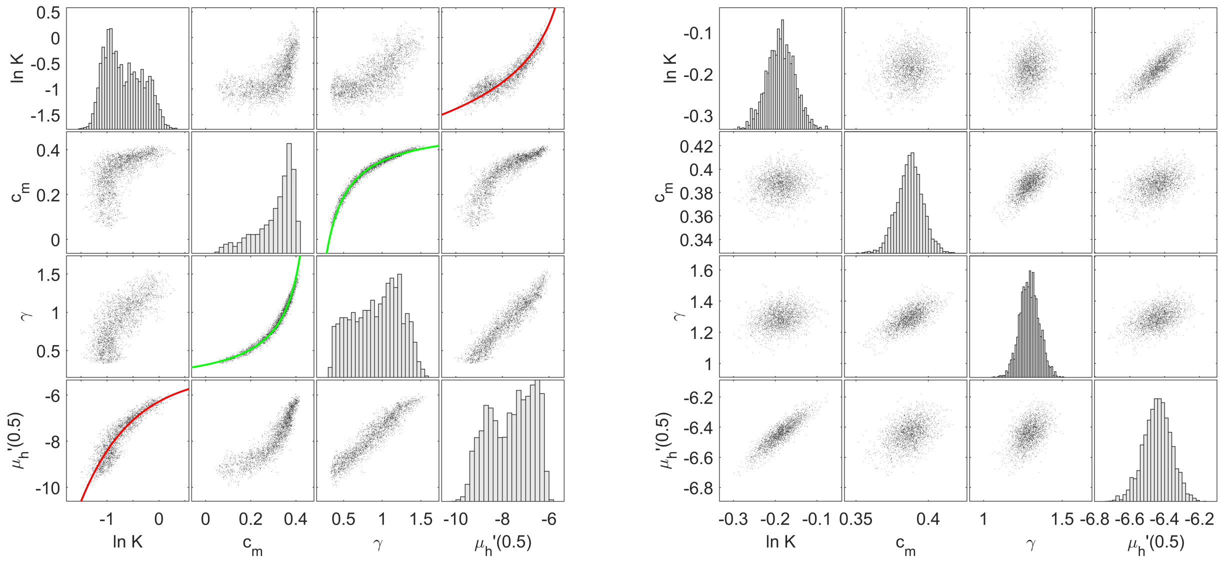

Figure S16: The scatter plots and histograms for each pair and each parameter in $\mathbf{p}$ generated by Hamiltonian Monte Carlo using UC-based (left. Eq. S103) and pixel-based (right. Eq. S101) likelihood (5000 samples), respectively. Red and green lines are fitted curves showing the correlation between K and p_{GL}, as well as c_m and γ, respectively.

We also define the squared error of a single particle i,

$$\mathrm{SE}_i = \sum_j \|c_{i,j}(\mathbf{p}_{\mathrm{global}}, \mathbf{Z}_i)) - c_{\mathrm{data},i,j}\|^2. \tag{S111}$$

6.2 Data preprocessing

Next, we describe in detail the preprocessing of experimental data before the inversion step to obtain the experimental concentration field $c_{\mathrm{data},i,j}$ in Eq. S108 and then how the squared error in Eq. S108 is computed.

Given the Li fraction and optical density at all the pixels, the image is binarized to produce the boundary of the particle. Triangulation is then performed on the particle geometry to be used as the mesh in the finite element simulation. The first frame of the images is interpolated onto the mesh and used as the initial condition. After solving the model, the solution is then interpolated onto a fine rectangular mesh (10 times finer than the image pixel), convolved by averaging over neighboring grid points that are within $[-0.5, 0.5]$ pixel size relative to itself in both x and y directions, and then downsampled onto the image pixel positions. We explain why convolution is performed below. Invalid pixels and pixels outside of the particle boundary are not included in the squared error.

The convolution is performed here because, in the experiment, X-ray absorption is measured at 706 eV and 713 eV at all pixels [1]. As described in 1.2, locally the optical densities (OD) relative to the OD at 703 eV (S_{706} and S_{713}) are linearly related to the local compositions by

$$\begin{pmatrix} S_{706} \\ S_{713} \end{pmatrix} = \begin{pmatrix} \mathrm{LFP}_{706} & \mathrm{FP}_{706} \\ \mathrm{LFP}_{713} & \mathrm{FP}_{713} \end{pmatrix} \begin{pmatrix} a \\ b \end{pmatrix}, \tag{S112}$$

where the matrix is the optical densities of LFP and FP references at 706 eV and 713 eV. Pixels where $a < 0$ or $b > 0$ are considered invalid. Recall that the mosaic miscibility gap $[c_1, c_2]$ is close to $[0, 1]$ ($c_1 = 0.013$), and c_1 is much smaller than the experimental error $\sigma_\epsilon = 0.07$ [26, 1]. Hence, concentration within $[0, c_1]$ and $[c_2, 1]$ cannot be reliably inferred from the experiment. As a result, we make the reasonable assumption that the LFP and FP references correspond to $c = c_1$ and $c = c_2$, respectively. By constraining the observed values to within $[c_1, c_2]$, we

allow data to inform physical properties only within this range, in the narrow regions of $[0, c_1]$ and $[c_2, 1]$, $\mu_h(c)$ and $j_0(c)$ will be informed by the prior. The representation for these two functions (Eqs. S26 and S36) and their prior determined their asymptotic behavior: $\mu_h \to \ln c$, $\ln j_0 \to \ln c$ as $c \to 0$, and $\mu_h \to \ln 1-c$, $\ln j_0 \to \ln 1-c$ as $c \to 1$. Because the first frame is used as the initial condition, the choice of references precludes values of 0 and 1 in the initial condition and hence avoids numerical issues due to the logarithmic terms in the chemical potential. Therefore in summary, $c_{\mathrm{data},i,j} = (c_2 - c_1)\frac{a}{a+b} + c_1$.

Following Ref. [1], we define the relative thickness of the particle to be $h(\mathbf{x}) = (a+b)/\overline{a+b}$, or the normalized optical density.

Suppose the measured optical density at the two energy levels and at each pixel is the convolution of true optical density and the same point spread function (PSF) (in the limit of small spatial variation), then the observed a and b are also the convolution of the true values of a and b (because the spatial convolution and the matrix above commute). We ignore the variation in $a+b$ within the extent of PSF, which is about the size of one pixel in our case. This assumption is reasonable given that the overall relative standard deviation of $a+b$ is 17% to 27% and that the variation within the extent of one pixel is unknown. Therefore, the observed Li fraction c'' is related to the true Li fraction c' by

$$c'' = \frac{c' \otimes \mathrm{PSF}}{\mathbb{I} \otimes \mathrm{PSF}}, \tag{S113}$$

where $\otimes$ denotes convolution, and $\mathbb{I}$ is the shape function for the particle (1 inside the particle and 0 outside).

Because the X-ray beam is focused using 45 nm to 60 nm zone plates, and the images are raster-scanned in 50 nm steps [1], hence we assume that PSF is a nonzero constant within a box of one pixel in width and height. Correspondingly, we average the values on the finer simulation grid within the box to obtain the model prediction value at the location of the pixel $c_{i,j}(\mathbf{p}_{\mathrm{global}}, \mathbf{Z}_i))$. The difference with the pixel value is the error.

6.3 Numerical solver

We use finite element to solve the model. The use of the regular solution model for thermodynamics forbids $c = 0$ and $c = 1$, therefore $c \in (0, 1)$. Numerically, if the Li fraction c is too close to 0 or 1 (when large overpotential is imposed), the solver might cause c to go beyond this range, leading to complex and hence nonphysical values in the equation due to the presence of $\ln \frac{c}{1-c}$ in $\mu_h(c)$. Therefore, to improve the robustness of the solver during optimization, we perform the nonlinear transformation

$$z(c) = \log \frac{c}{1-c}, \tag{S114}$$

or

$$c(z) = \frac{1}{e^{-z}+1}. \tag{S115}$$

We use the linear basis functions $\{\phi_i, i = 1, \ldots\}$ on the triangular mesh to represent the variables,

$$z(x) = \sum_i z_i \phi_i(x), \tag{S116}$$

and similarly for μ and u. Using the basis functions also as the test function, the weak form of the model described in sec. 2.1 is

$$\left\langle \frac{\partial c}{\partial t}, \phi_i \right\rangle = \langle R, \phi_i \rangle - \left\langle \frac{Dc}{k_B T} \nabla \mu, \nabla \phi_i \right\rangle \tag{S117}$$

$$\langle \mu, \phi_i \rangle = \langle \mu_h(c), \phi_i \rangle + \langle \kappa \nabla c, \nabla \phi_i \rangle + c_s^{-1} \sigma^0_{mn} \varepsilon^0_{mn} \langle c, \phi_i \rangle - c_s^{-1} \sigma^0_{mn} \langle u_{m,n}, \phi_i \rangle \tag{S118}$$

$$0 = \langle \sigma_{mn}, \phi_{i,m} \rangle$$

$$\text{or equivalently} \quad 0 = \langle \lambda_{mnop} u_{o,p}, \phi_{i,n} \rangle - \sigma^0_{mn} \langle c, \phi_{i,n} \rangle \tag{S119}$$

$$\left\langle \frac{\partial c}{\partial t}, 1 \right\rangle = I(t), \tag{S120}$$

where the bracket $\langle \cdot, \cdot \rangle$ denotes the inner product of two functions, that is, the integral over the domain. $u_{m,n}$ denotes the derivative of the mth component of the displacement field in the nth direction, $\phi_{i,n}$ denotes the partial derivative of test function ϕ_i in the nth direction, $I(t)$ is the interpolated average reaction rate. When essential boundary condition is imposed, the chemical potential equations at the boundary is replaced by the essential boundary condition.

The time derivative of Eq. S117 can be written in a form that is explicit in terms of dz_i/dt,

$$\left\langle \frac{\partial c}{\partial t}, \phi_i \right\rangle = \left\langle \frac{dc}{dz}\frac{\partial z}{\partial t}, \phi_i \right\rangle = \sum_j \left\langle \frac{dc}{dz}\phi_j, \phi_i \right\rangle \frac{dz_j}{dt}, \tag{S121}$$

and similarly Eq. S120 can be written as

$$\sum_j \left\langle \frac{dc}{dz}\phi_j, 1 \right\rangle \frac{dz_j}{dt} = I. \tag{S122}$$

The discretized equations are a set of differential algebraic equations (DAEs), where z is the dynamic variable (except for nodes at the boundary where essential boundary condition is imposed) and the other variables μ, $\mathbf{u}$, and $\Delta\phi$ are algebraic. The DAE is of the form

$$M\frac{dy}{dt} = F(t, y). \tag{S123}$$

We use an implicit solver with adaptive time stepping, adaptive order, and error control to solve the DAEs [73].

6.4 Temporal accuracy

Because an implicit adaptive DAE solver is used, the objective function may be discontinuous or nondifferentiable in certain regions. The objective function becomes smoother as the solver tolerance decreases (higher temporal accuracy). Here we study the effect of temporal accuracy by computing the objective function around a nominal set of parameters. We perform a preliminary optimization on all of the datasets, choose the result at the 4th iteration as the nominal parameters, and compute the objective function along a certain direction in the parameter space by varying one global parameter while keeping all other parameters fixed.

Fig. S17 shows the half squared error of all particles SE/2, and half squared error of the second particle $\mathrm{SE}_2/2$ as a function of the second parameter for $\mu_h(c)$ (a_2) as well as the gradient computed from adjoint sensitivity analysis (ASA). The tangent line with the slope of the computed gradient is shown as red lines. From left to right, the first case is when the solver solves c directly and the relative (RelTol) and absolute tolerance (AbsTol) of the DAE solver is 10^{-3} and 10^{-4}, respectively. We find that the objective function is highly noisy. Compared to the first case, the second case uses the z transformation in Eq. S114. Compared to the second case, the third case reduces the relative tolerance of the DAE solver to 5×10^{-4}, which reduces the roughness to some extent. The fourth case is the most stringent and compared to the second case, it reinitializes with a small time step at each frame, this significantly reduces the roughness of the objective function, and the slope agrees well with the adjoint sensitivity analysis.

Note that the adjoint and forward sensitivity analysis can return a sufficiently accurate gradient even with low accuracy such as in the first or second case. With increasing accuracy, the computational time increases. Therefore, the setting of the DAE solver tolerance should be balanced between the need for accuracy and time. With optimization, RelTol = 10^{-3} and AbsTol = 10^{-4} is often sufficient since the gradient calculated using forward sensitivity analysis and the approximated Hessian can lead to the minimum despite the rough landscape. Fig. S18 shows the objective function as a function of the parameters of $\mu_h(c)$ around the optimum found by the optimizer when DAE tolerance is RelTol = 10^{-3} and AbsTol = 10^{-4}. The objective function is computed using RelTol = 5×10^{-4} and AbsTol = 10^{-4} with reinitialization. We find that the lower accuracy used during optimization is sufficient to find the optimum.

However, when doing a gradient-based Monte Carlo sampling such as Hamiltonian Monte Carlo, we need to make sure that the roughness is not much larger than σ_ϵ^2; otherwise, the acceptance ratio becomes low and the

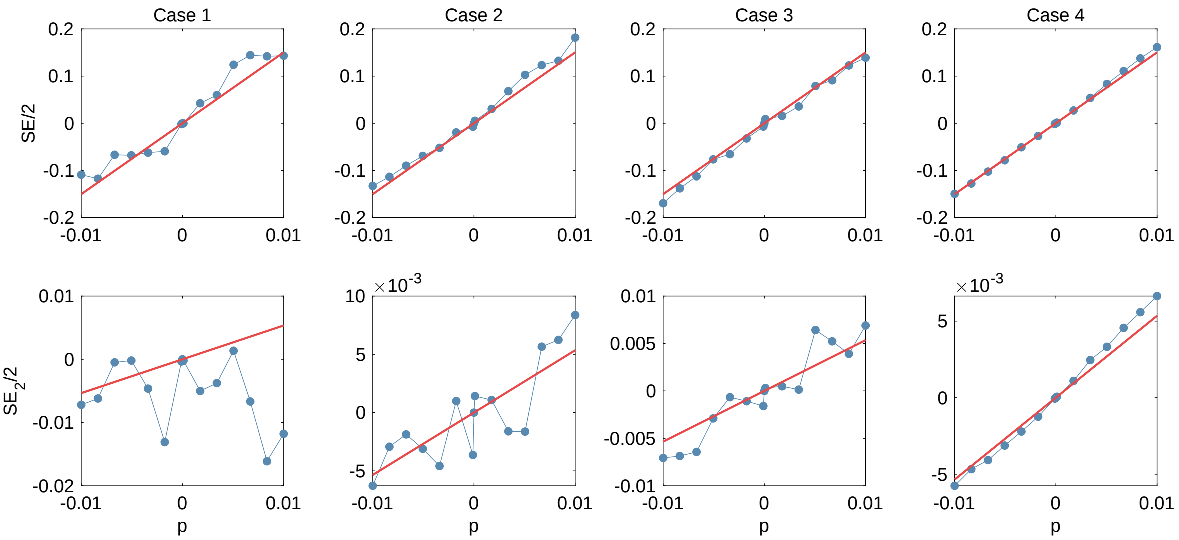

Figure S17: The objective function landscape in the direction of the second parameter for $\mu_h(c)$. The objective function and parameter are centered so that they are zero at the nominal parameters. The blue lines with markers correspond to the function value at discrete points. The red lines correspond to the tangent line at $p = 0$ computed by the adjoint sensitivity analysis. See the main text for the description of the different cases.

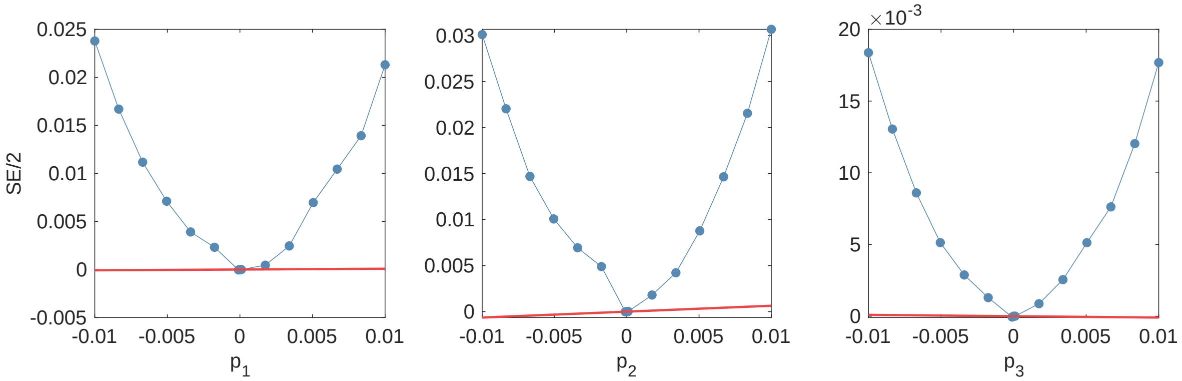

Figure S18: The objective function as a function of the three parameters of $\mu_h(c)$ at the optimum found by the optimizer. The blue lines with markers correspond to the function value at discrete points. The red lines correspond to the tangent line at $p = 0$ computed by the adjoint sensitivity analysis. Both are evaluated using RelTol = 5×10^{-4} and AbsTol = 10^{-4} and reinitialization at each frame.

Table S2: The roughness of the objective function. SE is the squared error of all particles, and SE_i is the squared error of individual particle. Three parameters of $\mu_h(c)$ a_1, a_2, and a_3 are varied. For the objective function of individual particles, the 25%, 50%, and 75% percentile roughness of all particles for all three parameters are tabulated. "reinit." stands for reinitialize the solver at each frame; "in c (no z)" stands for solving in the variable c, with all other cases solved using the z transformation.

	SE/2			$SE_i/2$		
Natural boundary condition	a_1	a_2	a_3	25%	50%	75%
RelTol = 10^{-3}, AbsTol = 10^{-4}, in c (no z)	0.024	0.026	0.030	1.7×10^{-4}	9.7×10^{-4}	23×10^{-4}
RelTol = 10^{-3}, AbsTol = 10^{-4}	0.019	0.015	0.015	1.6×10^{-4}	6.3×10^{-4}	17×10^{-4}
RelTol = 5×10^{-4}, AbsTol = 10^{-4}	0.013	0.010	0.010	1.3×10^{-4}	5.4×10^{-4}	11×10^{-4}
RelTol = 10^{-3}, AbsTol = 10^{-4} and reinit.	0.0045	0.0056	0.0037	0.5×10^{-4}	1.7×10^{-4}	3.2×10^{-4}
RelTol = 5×10^{-4}, AbsTol = 10^{-4} and reinit.	0.0039	0.0021	0.0050	0.4×10^{-4}	1.3×10^{-4}	2.9×10^{-4}
Essential boundary condition	p_1	p_2	p_3	25%	50%	75%
RelTol = 10^{-3}, AbsTol = 10^{-4}	0.031	0.011	0.030	1.1×10^{-4}	5.4×10^{-4}	12×10^{-4}
RelTol = 5×10^{-4}, AbsTol = 10^{-4}	0.0066	0.0060	0.0051	0.9×10^{-4}	3.1×10^{-4}	7.4×10^{-4}
RelTol = 10^{-3}, AbsTol = 10^{-4} and reinit.	0.0050	0.0035	0.0064	0.4×10^{-4}	1.4×10^{-4}	3.2×10^{-4}
RelTol = 5×10^{-4}, AbsTol = 10^{-4} and reinit.	0.0046	0.0036	0.0029	0.3×10^{-4}	0.9×10^{-4}	2.7×10^{-4}

sampling is inefficient. Here, we define the roughness of the objective function in the direction of parameter p by

$$\text{roughness} = \left[\frac{1}{\Delta p}\int_{p_0-\Delta p/2}^{p_0+\Delta p/2}\left[S(p) - \left(S(p_0) + \frac{\partial S}{\partial p}(p_0)(p-p_0)\right)\right]^2 dp\right]^{1/2}. \tag{S124}$$

The integral is approximated using the trapezoidal rule using the points shown in Fig. S17 and $\Delta p = 0.01$. $\partial S/\partial p$ is computed from ASA. Table S2 lists the roughness of the objective function SE/2 including all particles in the direction of 3 parameters for $\mu_h(c)$ under different solver accuracy, as well as the 25%, 50%, and 75% percentile of the roughness of $SE_i/2$ for the same three parameters. We find that using the z transformation in Eq. S114 decreases the roughness. Decreasing the solver tolerance and reinitialization decrease the roughness.

The roughness for individual particles is on the order 10^{-4}, and much smaller than $\sigma_\epsilon^2 = 4.9 \times 10^{-3}$. Hence HMC performed on a few particles will only need a low-accuracy time integrator. However, the objective function including all particles has a much higher roughness. In fact, when reinitialization is not applied, the roughness is much larger than σ_ϵ^2, resulting in inefficient sampling. Hence, when HMC is performed on all particles, a much tighter DAE tolerance is suggested. If the solver error is independent for all particles, the roughness scales with the square root of the number of particles $\sqrt{N}$.

6.5 Spatial accuracy

The finite element solver uses a fixed mesh. The mesh size needs to be fine enough to resolve the gradient in the concentration field $c(x)$ when phase separation occurs or in the presence of strong spatial heterogeneity. At the same time, since the computation time is dominated by model evaluation, the scale of the problem (particle size and mesh size) determines the computational cost of the inversion and uncertainty quantification. In this section, we discuss the tradeoff between fidelity and computational cost.

The important length scales are the characteristic length of the particle and the thickness of the interface (between the two phases in a phase-separated particle). The interfacial thickness is found to be 12nm $\approx 4\sqrt{\kappa}$ [20] in a coherently phase-separated particle. In comparison, the pixel size of the STXM images is 50 nm, which is unable to resolve the interface.

We perform simulations on two particles whose major axis lengths are 0.9 and 1.7 μm, respectively (300 and 1000 pixels in STXM image unit). We study the error and computational time as a function of the maximum mesh

size.

The particles are subject to two different constant total reaction rates chosen to correspond to those observed in the experiments that lead to the largest and smallest uniformity coefficients, which are $-0.3\ \mathrm{hr}^{-1}$ (de-insertion) and $2\ \mathrm{hr}^{-1}$ (insertion). Since the uniformity of $c(x)$ is very different in these two cases, this study is designed to test the numerical accuracy at different levels of concentration gradient. It is expected that finer mesh is needed to resolve features of large spatial gradient while the gradient decreases with increasing uniformity. The simulations are performed using the MAP result in 5.1. The same random fields are generated and scaled to the sizes of different particles.

The L_2 norm of the error is defined by

$$\text{Error} = \max_i \left[\int \|c(x,t_i) - \tilde{c}(x,t_i)\|^2 dV\right]^{1/2}, \tag{S125}$$

where t_i is the ith time point, c is the concentration field at a given mesh resolution, and $\tilde{c}$ is the solution on a sufficiently fine mesh (whose maximum mesh size is $0.3\sqrt{\kappa} = 0.89\text{nm}$) and is considered to be the truth. The integral is evaluated through quadrature and by projecting the coarse solution c onto the fine mesh.

Fig. S19 shows the L_2 norm of the error and computational time as a function of maximum mesh size $h_{\max}$. We also show the convergence of the largest magnitude of concentration gradient. The error and the gradient $\|\nabla c\|$) are reported in the unit of STXM pixel size (50 nm) and its inverse, respectively on the left axes. $h_{\max}$ is normalized by $\sqrt{\kappa}$ at the bottom axes. The corresponding values in nm are shown at the upper and right axes. The error scales as $h_{\max}^{1.4}$. The computational time scales as $h_{\max}^{2.4}$ (or about $N_{\text{node}}^{1.2}$ where N_{node} is the number of nodes in the triangular mesh), which suggests that the costs of model evaluation for the experimentally observed particles differ by at least 16 (10-fold difference in the area). Since the time integrator has an adaptive time step, the computational time also depends on the reaction rate. The slower and negative reaction enhances the nonuniformity and increases the computational time. Note that the error for two particles of different sizes are close and the error in the largest magnitude of concentration gradient converges very slowly, suggesting that the L_2 error mostly comes from the error at the interface. In fact, when the mesh is very coarse, $\max\|\nabla c\|$ approaches its upper bound $h_{\max}^{-1}$ (gray curve in Fig. S19c), suggesting a sharp transition across the distance of one triangular element. The computational time for the two particle sizes collapses onto the same curve based on the number of nodes.

To put the magnitude of the numerical error into perspective, for an error of 10, the average error per pixel for a particle of area 300 and 1000 is 0.03 and 0.01. In comparison, the standard deviation of the pixel error is 0.07 [1].

Fig. S20 visualizes the numerical error using two sets of mesh whose maximum mesh size is 20 nm and 5 nm, respectively, (or 0.4 and 0.1 in STXM pixel unit), which are equal to $6.7\sqrt{\kappa}$ and $1.7\sqrt{\kappa}$ (highlighted as vertical dashed lines in Fig. S19). The error is about 16 and 2.2, respectively (the error per pixel for a particle of area 300 is 0.05 and 0.016). We visualize the solution on the coarse mesh and the error compared to the fine mesh result. We find that for the Li de-insertion case with a large spatial gradient, most of the error comes from the region near the interface and regions of large concentration variation, while the position of the interface is well captured despite the low spatial resolution. For the Li insertion case with high uniformity coefficient, the error is small and more evenly distributed, the error using mesh size 5 nm is dominated by the DAE solver error (10^{-4}).

In summary, we find that the error of 5 nm mesh size is significantly smaller than the experimental error. However, in our study, the computational cost of optimization, cross-validation, and uncertainty quantification (Hamiltonian Monte Carlo or bootstrapping) can be high, since simulations must be performed for a large number of particles and for many repeated model evaluations.

Next, we compare the inversion result using 20 nm and 5 nm mesh size in Fig S21. The maximum $j_0(c)$ differs between the two meshes, due to insensitivity to the magnitude of $j_0(c)$, but the scaled $j_0(c)$ and $\mu_h(c)$ from the two are sufficiently close. We also show examples of inverted $\ln k(\mathbf{x})$ in Fig. S22. The inverted $\ln k(\mathbf{x})$ is interpolated onto image pixels and compared. We find that $\ln k(\mathbf{x})$, when subtracted by the mean of each particle, differ only slightly, much less than its uncertainty (see uncertainty quantification of $\ln k(\mathbf{x})$ in Section 9.1). Therefore, for computational efficiency, we use a maximum mesh size of 20 nm for cross-validation and uncertainty quantification.

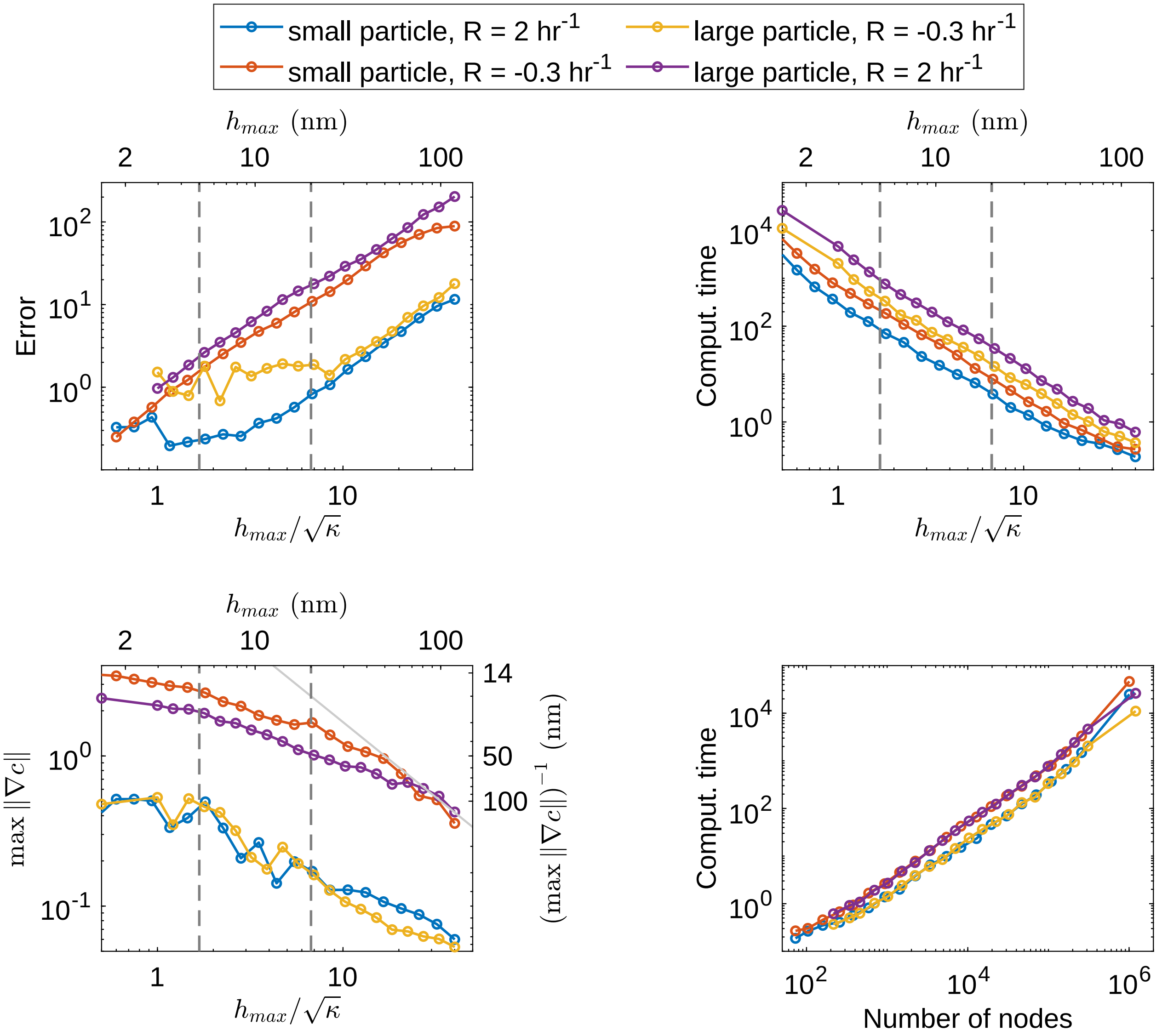

Figure S19: Error, computational time, and convergence analysis based on simulated data. The horizontal axis is the maximum mesh size $h_{\max}$ normalized by $\sqrt{\kappa}$, or in physical unit nm, or the total number of nodes in the mesh. The dimensionless error and $\|\nabla c\|$ are based on STXM pixel size. The areas for the small and large particles are 300 and 1000 pixels, respectively. The vertical dashed lines correspond to two sets of mesh used in performing forward simulation and inversion tests.

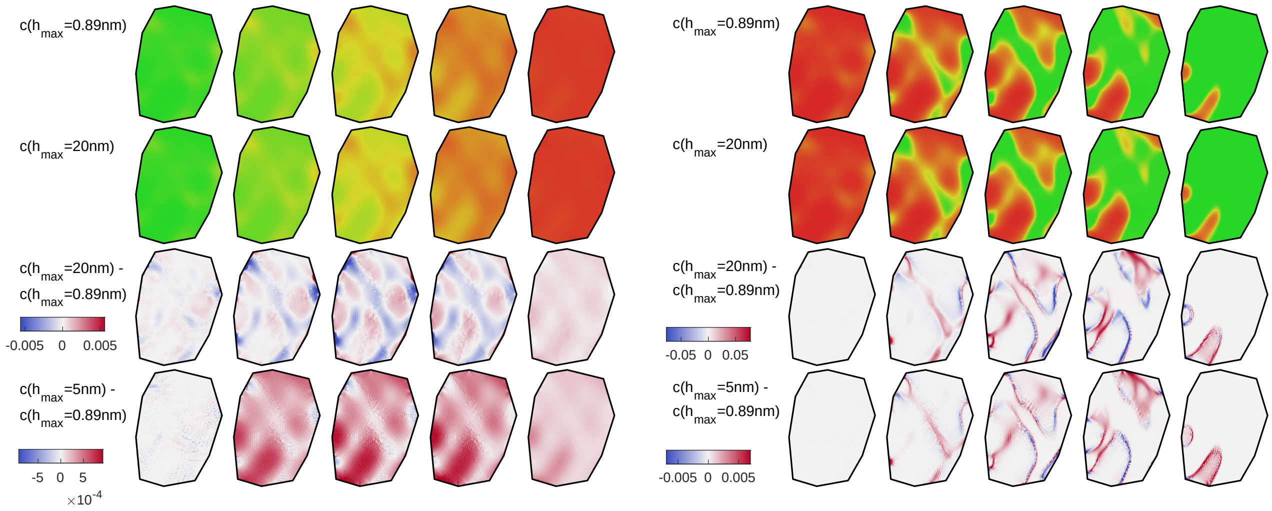

Figure S20: A comparison of solution using different mesh sizes at reaction rate $R = 2\ \text{hr}^{-1}$ (left) and $R = -0.3\ \text{hr}^{-1}$ (right). The first row is solved using a very fine mesh and is considered to be an approximation of the truth. The second row is the solution on a coarse mesh. The third and fourth rows are errors using different mesh sizes indicated on the left. All concentration and error fields are interpolated onto the same grid size (100×136).

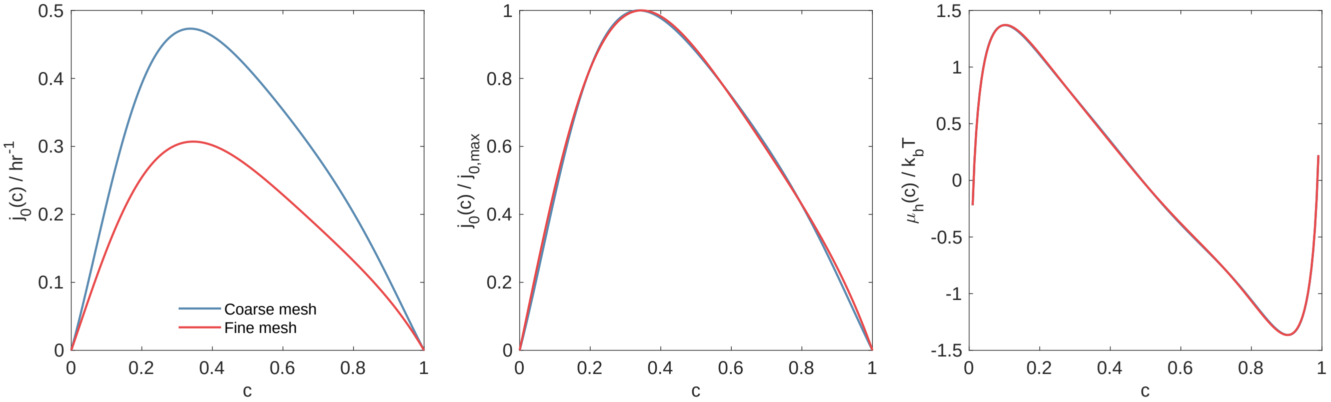

Figure S21: The MAP estimate of $\mu_h(c)$ and $j_0(c)$ using a coarse (maximum mesh size equals 40% pixel size, or 20 nm) and fine mesh (maximum mesh size equals 10% pixel size, or 5 nm).

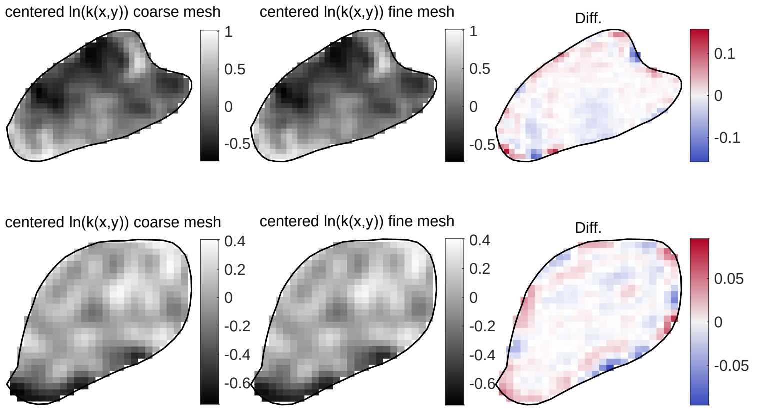

Figure S22: Centered $\ln k(\mathbf{x})$ of two select particles inverted using a coarse and fine mesh (see Fig. S21) and their difference (the latter subtracted by the former)

6.6 Optimization algorithm

To minimize the objective function, each iteration of the optimization performs the evaluation of the model and the sensitivities for all episodes in parallel. In this section, we compare quasi-Newton with the BFGS and trust-region algorithms. We set the initial guess of $\mu_h(c)$ to be the same as the model in Ref. [20], $j_0(c) = 4c(1-c)$, and $\ln k(\mathbf{x}) = 0$.

For quasi-Newton, we use adjoint sensitivity analysis to compute the gradient of the objective function. In Fig. S23, we show $\mu_h(c)$, $j_0(c)$, and the variance of $\ln k(\mathbf{x})$ at each iteration. Because $\mu_h(c)$ and $j_0(c)$ are shared by all simulations, the gradient with respect to these parameters are much greater than the parameters for $\ln k(\mathbf{x})$. At the initial guess, the L_2 norm of the gradient of all parameters for $\mu_h(c)$ and $j_0(c)$ (a_i ($i = 1, \ldots, N_\mu$) and b_i ($i = 1, \ldots, N_J$)) is 676 (root mean square is 104), that that for $\ln k(\mathbf{x})$ ($\mathbf{Z}_i$ ($i = 1, \ldots, N$)) is 104 (root mean square is 3.3). Therefore dyrubg the first few iterations of the optimization (gradient descent), it is mostly $\mu_h(c)$ and $j_0(c)$ that are updated while $\ln k(\mathbf{x})$ stays close to 0. Eventually, this leads to an exchange current that is very small and converges to a large objective function value and large residual gradient.

For the trust-region algorithm, we use forward sensitivity analysis to compute the gradient of the objective function and Gauss-Newton approximation of the Hessian [31, 32] (Eq. S107). In comparison, Fig. S24 shows that using FSA the optimizer converges quickly to a local minimum in about 20 iterations, with a final objective function smaller than ASA. Compared to ASA, $\mu_h(c)$, $j_0(c)$, and $k(\mathbf{x})$ are updated simultaneously. Therefore, throughout the manuscript, we use the trust-region algorithm for optimization.

6.7 Interpolation of average composition

As explained in the methods (PDE-constrained optimization), the average Li fraction of each frame is interpolated in time to give the average reaction rate. This section discusses the choice of interpolation method. We consider two interpolating methods: spline and shape-preserving piecewise cubic Hermite interpolation (pchip). Both methods are piecewise cubic polynomials and have continuous first-order derivatives. The former has a continuous second-order derivative, and the latter preserves the monotonicity of the interpolated data, but its second-order derivative may be discontinuous.

To quantify the error due to the interpolation, we simulate with nominal parameters used in Section 5.1 in a 1.5 μm × 1.5 μm periodic domain on a uniform 512 × 512 grid. The correlation length l is set to be 75 nm and $\sigma_\psi = 0.2$. The reaction rate is constant in time and the average Li fraction goes from 0.05 to 0.95 or backward. The domain is divided into 3 × 3 500 nm × 500 nm subdomains. The average Li fraction $\bar{c}$ of each domain is recorded. Five equally spaced points from the beginning to end are selected and the average fractions at these time points are used as known data points. Time is normalized for all cases to be $\bar{t} \in [0, 1]$. We compute the error between the

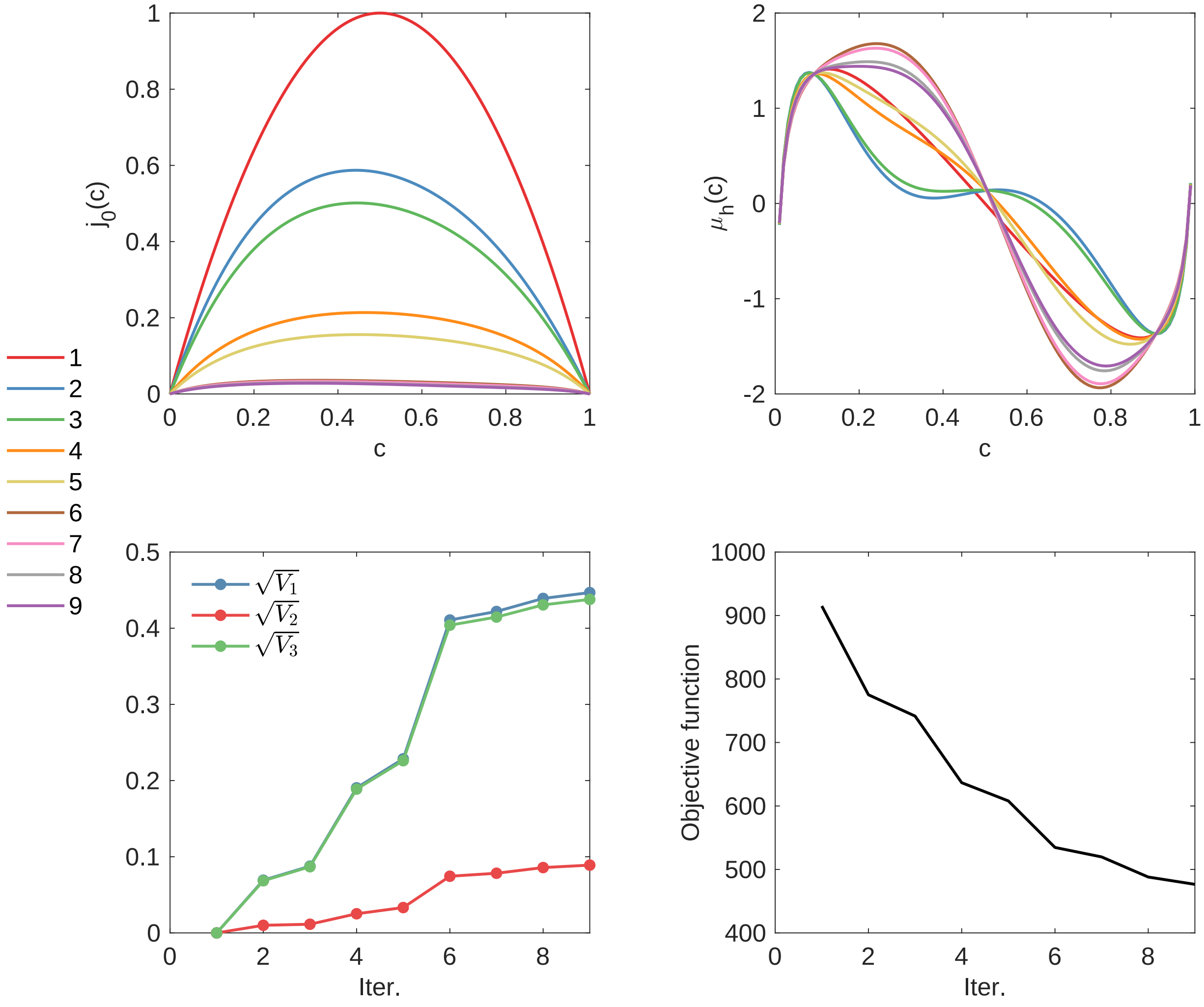

Figure S23: Optimization using quasi-Newton algorithm. $j_0(c)$, $\mu_h(c)$, variance of $\ln k(\mathbf{x})$, and the objective function at each iteration during optimization is shown. See the legend on the left for the color of each iteration.

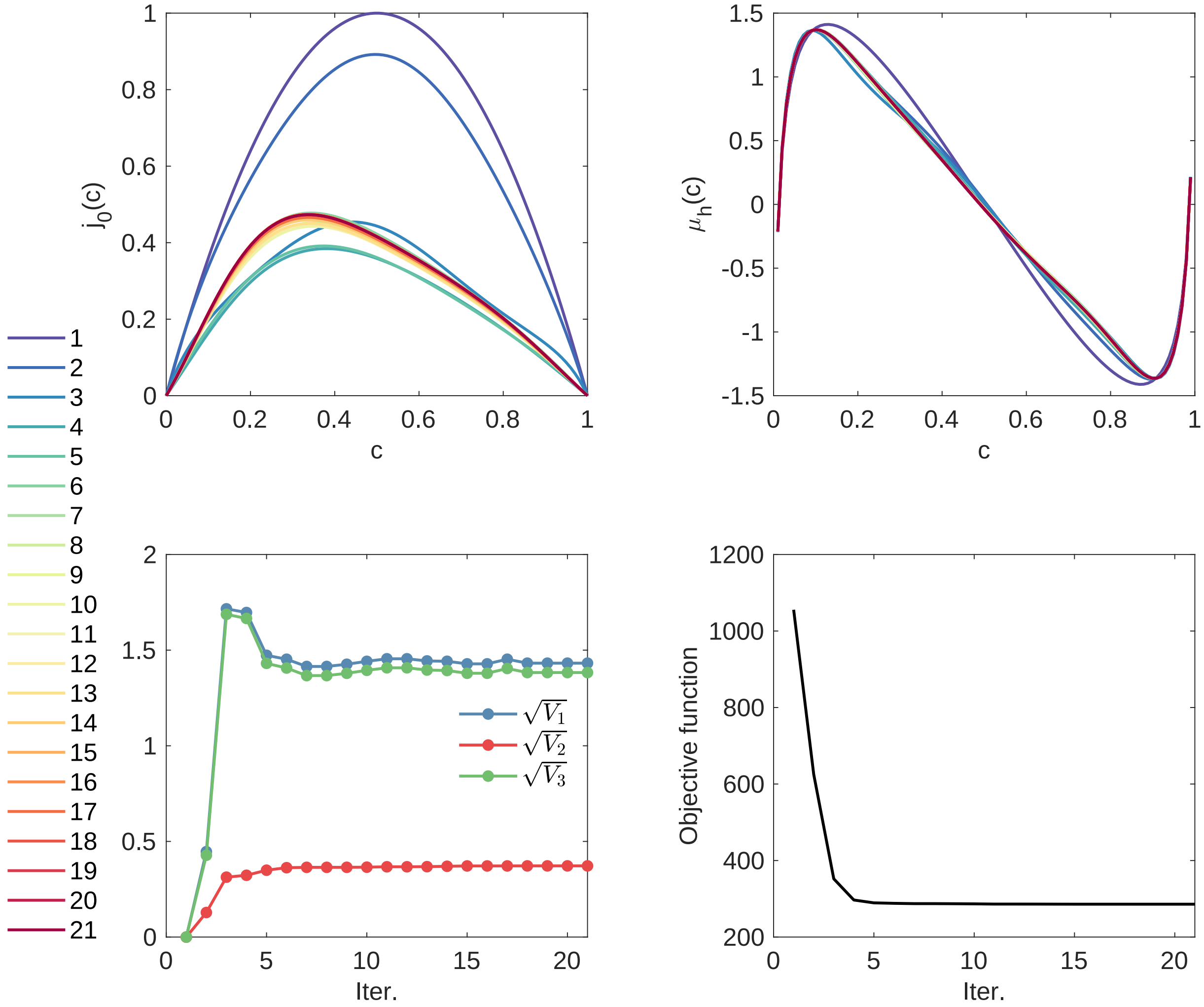

Figure S24: Optimization using trust-region algorithm.

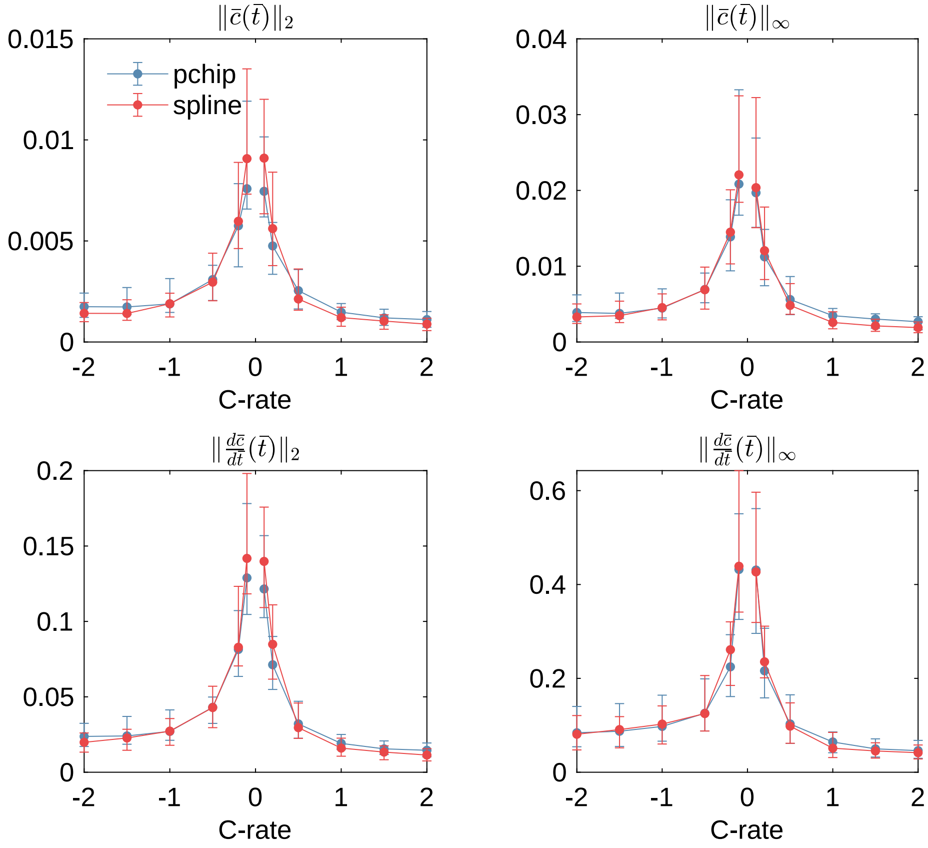

Figure S25: The median, 25%, 75% quantile of the error based on 9 subdomains and 2 realizations. The error is defined by 2-norm of $\infty-$norm of the difference between interpolated and true average fraction trajectories or the first derivative in time

actual average composition and that of interpolated composition: $\|\Delta\bar{c}(\bar{t})\|_2 = \left[\int_0^1 \left(\bar{c}_{\text{interp}}(\bar{t}) - \bar{c}_{\text{truth}}(\bar{t})\right)^2 d\bar{t}\right]^{1/2}$ and $\|\Delta\bar{c}(\bar{t})\|_\infty = \max_{\bar{t}} \bar{t}|\bar{c}_{\text{interp}}(\bar{t}) - \bar{c}_{\text{truth}}(\bar{t})|$, where $\bar{c}_{\text{interp}}$ is the interpolated average fraction based on the five data points and $\bar{c}_{\text{truth}}$ is the true average fraction from the simulations. We also compare the norm of the error of the first derivative $\frac{d\bar{c}}{d\bar{t}}$, which is the average reaction rate.

We run the simulations using 5 different realizations of the random field. Fig. S25 shows the median, 25%, 75% quantile of the error of all subdomains and all realizations. We see that the difference in error between spline and pchip is negligibly small. The error increases with decreasing reaction rate, due to the enhanced tendency to phase separate and hence increasingly nonlinear $\bar{c}(t)$ trajectory.

Fig. S11 shows the average composition of all particles and episode (dots) and the interpolated trajectory $\bar{c}(t)$ (solid lines) using pchip. The data in the same plot corresponds to the particles in the same batch of experiments and the same charge or discharge episode. Time is synchronized and shifted such that the time of the first frame is 0. The title of each plot is the global rate of charge of discharge. In contrast, Fig. S26 shows the interpolation using spline. Because spline interpolation does not preserve non-monotonicity, it is possible that the average composition overshoots to values beyond $[0, 1]$. Therefore, the spline is performed on $z = \ln\frac{\bar{c}}{1-\bar{c}}$, and then transformed back to $\bar{c}(z(t))$. Significant oscillations are observed. In view of this feature and the error analysis above, we choose pchip interpolation.

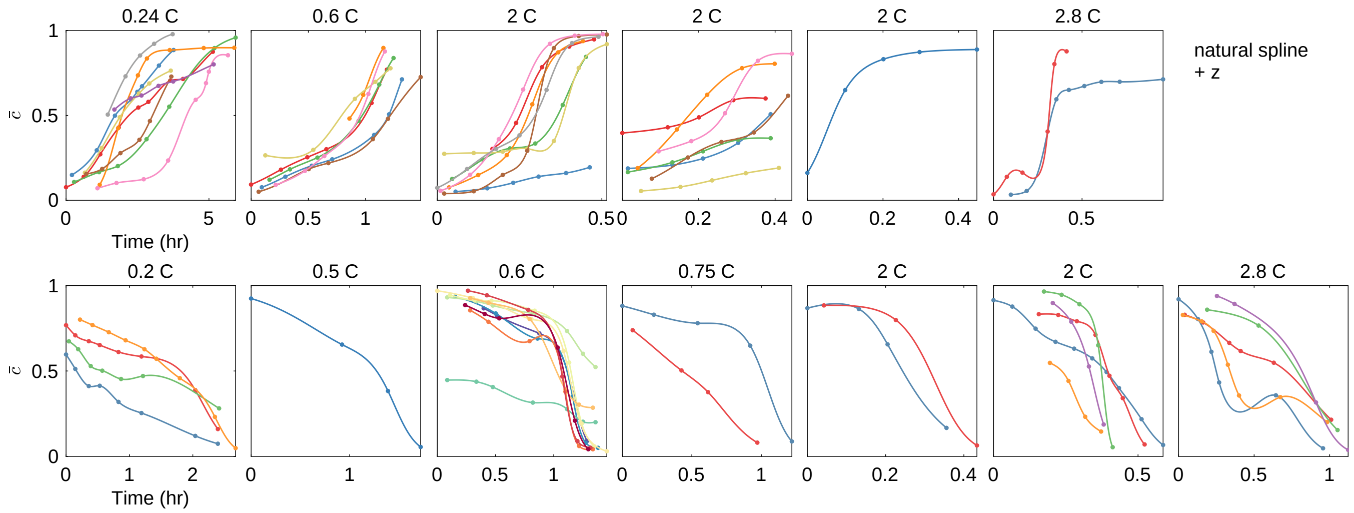

Figure S26: The average Li composition from experimental data (dots) and the interpolated trajectory using spline on the transformed variable z.

6.8 Function representation

Recall that we consider two representations for $j_0(c)$ (Eqs. S35 and S36).

We test the optimization using these representations starting from an initial guess of $b_n = 0$ ($n = 0, 1, \ldots$) for both representations and $N_J = 5$. Regularization on $\ln k(\mathbf{x})$ is imposed ($\rho_2 = 0.01$, see Section 8.5 and the main text). The natural boundary condition $\mathbf{n} \cdot \nabla c = 0$ is used. Fig. S27 shows that the MAP ($\mu_h(c)$ and $j_0(c)$) found by the optimizer using the two different representations are close, $j_0(c)$ are close to zero as c approaches 0 or 1 in both cases. Fig. S27 also shows the uncertainty estimated by the Hessian at the MAP using the Gauss-Newton approximation ($\sigma_\epsilon = 0.07$). The shaded region is the 99% confidence interval. We find that the uncertainty of $j_0(c)$ is mostly in its magnitude and not the functional form and that MAP of both representations lies within the confidence interval of each other.

Numerically, the computational time using the second representation is faster than the first, because the second forces the exchange current to approach 0 near $c = 0$ or 1, reducing the strong dependence of the reaction rate on c because of the large slope of $\mu_h(c)$ near $c = 0$ and 1.

The squared errors found using the first and second representations are 594.9 and 587.8. The objective functions $S(\mathbf{p})$ are 301.5 and 297.9. The former is 1.2% higher than the latter.

In summary, since the results of $j_0(c)$ using the two representations are similar, the second representation finds a smaller objective function and squared error, and the second is computationally more efficient. The second representation is used in the main text.

6.9 Boundary condition

We consider two different boundary conditions: the natural and essential boundary conditions. Following the relaxation simulation in Section 3, we assume no wetting when using the natural boundary condition $\mathbf{n} \cdot \nabla c = 0$. When the essential boundary condition is used, we interpolate the data at the particle boundaries in time using pchip, for the same reason as the interpolation of the average concentration, and impose the time-dependent concentration at the boundaries in the model. Fig. S28 shows the MAP ($\mu_h(c)$ and $j_0(c)$) using either boundary conditions (using the second representation for $j_0(c)$, Eq. S36). Regularization on $\ln k(\mathbf{x})$ is imposed ($\rho_2 = 0.01$, see Section 8.5). We find that the resulting normalized $j_0(c)$ and μ_h using the two boundary conditions are very close. The difference in the magnitude of $j_0(c)$ is due to the insensitivity of the objective function with respect to Z_0: a change in the boundary condition or other parameters causes a large shift in Z_0 (see Section 8.3).

Fig. S29 shows two examples of $\ln k(\mathbf{x})$ (subtracted by the mean), which shows that the difference is small (much

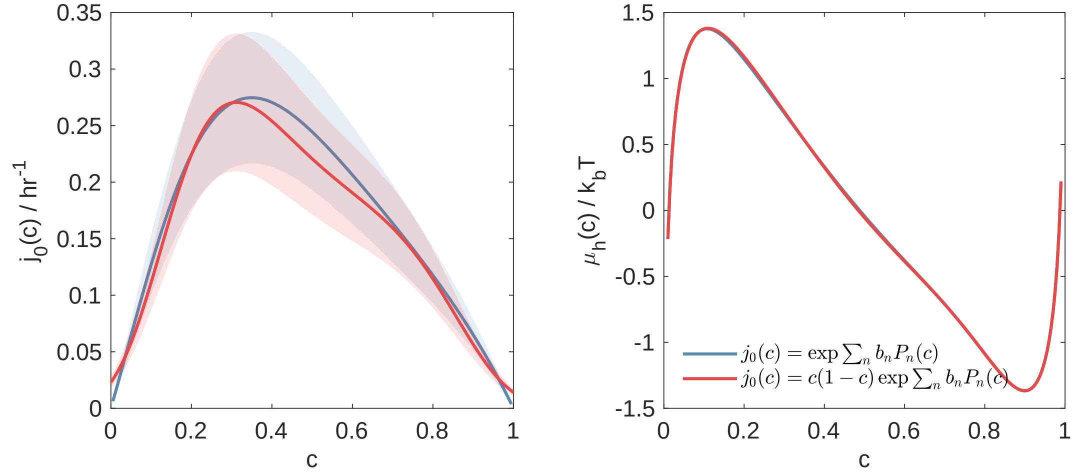

Figure S27: The MAP estimate of $\mu_h(c)$ and $j_0(c)$ and the 99% confidence interval estimated using the Hessian at MAP. Natural boundary condition is used for the model.

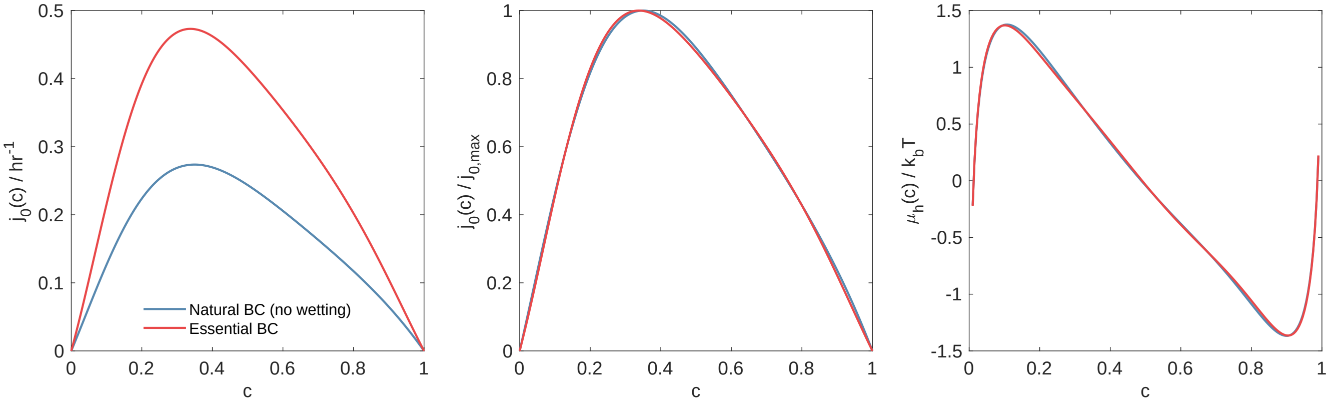

Figure S28: The MAP estimate of $\mu_h(c)$ and $j_0(c)$ using the natural and essential boundary conditions.

smaller than the uncertainty in $\ln k(\mathbf{x})$, see Section 9.1) and the difference mostly occurs along the boundaries.

Using the same procedure as in Section 6.8, we compare the MAP and uncertainty (estimated using linear approximation) results using two different representations of $j_0(c)$ using essential boundary condition in Fig. S30. The total SE of the first and second representations are 569.9 and 563.8. The objective functions $S(\mathbf{p})$ are 288.8 and 285.9. The former is 1.0% higher than the latter. The training error using essential boundary condition is smaller than natural boundary condition in Section 6.8. Therefore in the main text and the rest of the supplemental information, we represent $j_0(c)$ using Eq. S36 and use the essential boundary condition.

7 Identifiability

This section discusses the identifiability of the model based on simulated data. The simulations are performed on three randomly shaped convex polygons with the nominal parameters used in Section 5.1. The particles have on average 459 pixels and 5 frames. The first particle is cycled at 0.3C discharge and 2C charge, the second at 2C discharge and 0.2C charge, and the third at 0.6C discharge and charge.

Using Hamiltonian Monte Carlo (HMC), Fig. S31 shows that at the current level of pixel error of $\sigma_\epsilon = 0.07$ the posterior distribution of $\mu_h(c)$, $j_0(c)$ and $k(\mathbf{x})$ are distributed around the truth. Notice that in order to improve the chain mixing of HMC, we perform a parameter space transformation based on Cholesky decomposition of the Hessian at the truth, $H_{L_2} = \mathbf{R}^*\mathbf{R}$. The transformed parameters are $\mathbf{p}' = \mathbf{R}\mathbf{p}$. HMC is performed in the space of $\mathbf{p}'$,

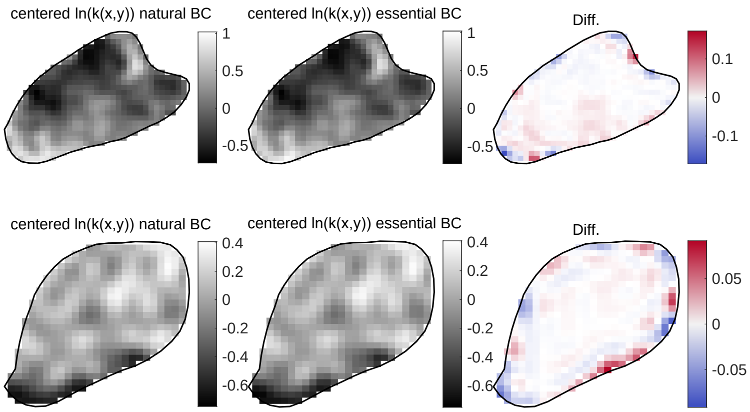

Figure S29: Centered $\ln k(\mathbf{x})$ of two select particles obtained using either boundary conditions and their difference (the latter subtracted by the former).

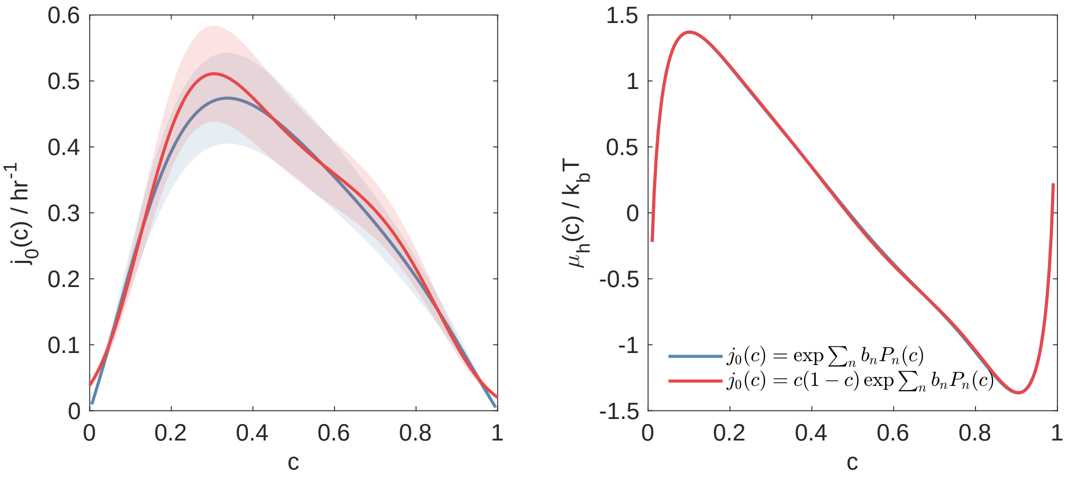

Figure S30: The MAP estimate of $\mu_h(c)$ and $j_0(c)$ and the 99% confidence interval estimated using the Hessian at MAP. The essential boundary condition is used for the model.

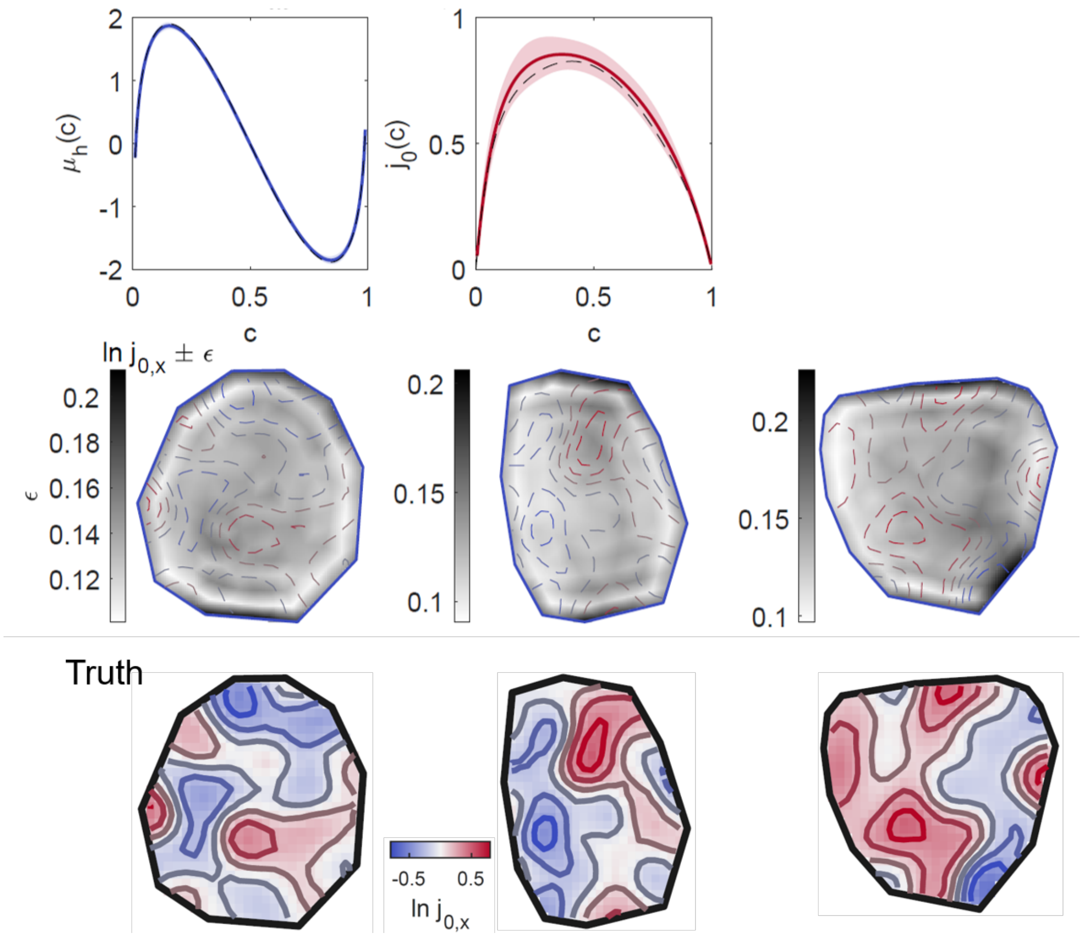

Figure S31: The posterior distributions obtained from HMC. For $\mu_h(c)$ and $j_0(c)$, dashed lines are the truth, solid lines are the posterior mean, the shaded region is the 99% confidence interval. For $\ln k(\mathbf{x})$, the contours lines are posterior mean with the colors same as the plots of the true $\ln k(\mathbf{x})$. The gray scale images are the uncertainty $\epsilon = z_p\sqrt{\mathbb{V}\mathrm{ar}[\ln k(x)]}$, here $z_p = 2.576$.

which is significantly more efficient than sampling $\mathbf{p}$. The resulting mean step size is 0.0051 with an acceptance ratio of 0.677. Based on the first and last third of the chain, the maximum Gelman–Rubin diagnostic is 1.047.

Next, we perform the optimizations starting at different initial guesses. Regularization is not included. The simulated data are created using the exact same model and solver that the optimizer uses and has no noise. So ideally the optimizer should be able to find the exact set of parameters that the simulated data are generated with. However, because the DAE solver is an adaptive and fully implicit solver, the numerical error and the roughness of the objective function landscape will cause the converged parameters to deviate from the truth. Such deviation must be controlled within a tight error that is comparable to the numerical error of the solver and tolerance of the optimizer. If this is not true, then that suggests multiple optimal solutions exist and the parameters are nonidentifiable.

Fig. S32a shows the sorted final objective function ($S(\mathbf{p}) = \mathrm{RMSE}^2 \cdot \sum_i A_i N_{T,i}/2$, where A_i is the particle area and $N_{T,i}$ is the number of frames for particle i excluding the initial frame. Note that in Fig. S32 particle sizes are scaled such that the average long axis length of the particles is nondimensionalized to be 1) as well as the corresponding RMSE starting from 500 randomly drawn initial guesses when the DAE solver has a relative tolerance of 10^{-3} and absolute tolerance of 10^{-4}, when the optimizer tolerance is 10^{-6} and 10^{-9}, and when κ is excluded or included in the optimization. The optimizer terminates when function tolerance (function difference between consecutive iterations less than Tol$\cdot$(1+FunVal), where FunVal is the function value of the previous step, absolute step tolerance, or the absolute optimality (infinity norm of the gradient at the current step) tolerance meets the aforementioned

threshold. When κ is set to be the truth and not optimized, the final RMSE drops below the dashed line, which is the absolute tolerance of the DAE solver. The corresponding error in the objective function due to numerical errors is estimated to be 6.8×10^{-8} (the square of absolute error multiplied by total area and number of frames, excluding the initial frame). Decreasing the optimizer tolerance decreases the final objective function further, while when the optimizer tolerance is 10^{-9} there is a still large proportion of the final objective function that stays close to the dashed line, which suggests that the optimizer may be limited by the error of the forward sensitivity solver that computes the gradient and Hessian. When κ is unknown and included as a free parameter in the optimizer even when the optimizer tolerance is 10^{-9}, more than 80% of the solutions are above the DAE absolute error and the plot is tilted without a plateau region, suggesting the nonidentifiability of the problem when κ is unknown. Therefore, in this study, we will use the literature value for κ.

Figs. S32bcd show the RMSE of the $\mu_h(c)$, $\ln j_0(c)$, and $\ln k(\mathbf{x})$ that correspond to the sorted objective functions in Fig. S32a. The RMSE of inferred function $a(x)$ is defined by $\left(\int (a(x) - a_{\text{truth}}(x))^2 dx\right)^{1/2}$, where $a_{\text{truth}}(x)$ is the known truth. We see that when RMSE for the concentration field $c(x)$ is 10^{-4}, the error for the inferred functions is around 10^{-3}. This is the level set by the DAE solver tolerance. When κ is unknown, the error for the inferred functions can greatly exceed this level, suggesting their nonidentifiability.

Fig. S32e compares the sorted final objective function using low and high DAE solver tolerance. The high tolerance is the same as above, while the lower one corresponds to a relative tolerance of 10^{-6} and absolute tolerance of 10^{-9}. The dashed line is the optimizer tolerance. Switching to a lower DAE tolerance (higher accuracy) reduces the objective function, eliminating the region that is limited by the solver accuracy. Fig. S32f shows the corresponding RMSE of the inferred functions with the lower DAE tolerance.

In comparison with the result on the spatial accuracy in Section 6.5, a particle of 300 STXM pixels with the discretization used in the study above has a maximum MSE of 3.5×10^{-3}. Therefore a large percentage of the results that the optimizer converges to can be considered to have found the truth.

8 Cross-validation

As mentioned in the main text, regularization (especially that of spatial heterogeneity) is essential to prevent overfitting. This section discusses in detail the effect of regularization, the sensitivities of parameters and cross-validation to determine the optimal regularization parameter.

8.1 Regularization

In this section, we use simulated data to study the effect of regularization on the global parameters $\mathbf{p}_{\text{global}}$ (ρ_1) and parameters for the heterogeneity $\mathbf{Z}_i$ (ρ_2). We use the same simulated datasets as used in section 7 with added noise that corresponds to a pixel-wise error $\sigma_\epsilon = 0.07$ that is consistent with experimental images. We perform regularization test on the three particles above, each having two episodes at 0.2C (charge and discharge), 0.3C, and 0.6C, and use the same particles at 2C (charge or discharge) as validation dataset.

Fig. S33 shows the validation error (RMSE) as a function of ρ_1 and ρ_2. The validation error shows a minimum near $\rho_1 = \rho_2 = 10^{-4}$, however, the minimum is not sensitive to the value of ρ_1. This is also illustrated in Fig. S33b which shows a negligible change in the validation error as a function of ρ_1 when $\rho_2 = 0$. On the other hand Fig. S33c shows significant reduction of the validation error at its minimum varying ρ_2 when $\rho_1 = 0$. Correspondingly, in Fig. S33d, the L_2 norm of the error of $\ln k(\mathbf{x})$ also shows a minimum around the ρ_2 that has the minimum validation error. In contrast, as shown in Fig. S33e, the L_2 norm of the error of $\mu_h(c)$ and $\ln j_0(c)$ shows negligible or no decrease as ρ_2 varies, instead, they increase sharply when $\rho_2 > 10^{-4}$.

8.2 Variance and bias of MAP estimate

Understanding the variance and bias of the MAP estimate with regularization is important for cross-validation. In this section, we use simulated data generated using the same method as in sec. 7 to study the scaling of the variance and bias of the MAP estimate for the global parameters $\mathbf{p}_{\text{global}}$ for $\mu_h(c)$ and $\ln j_0(c)$ with respect to the

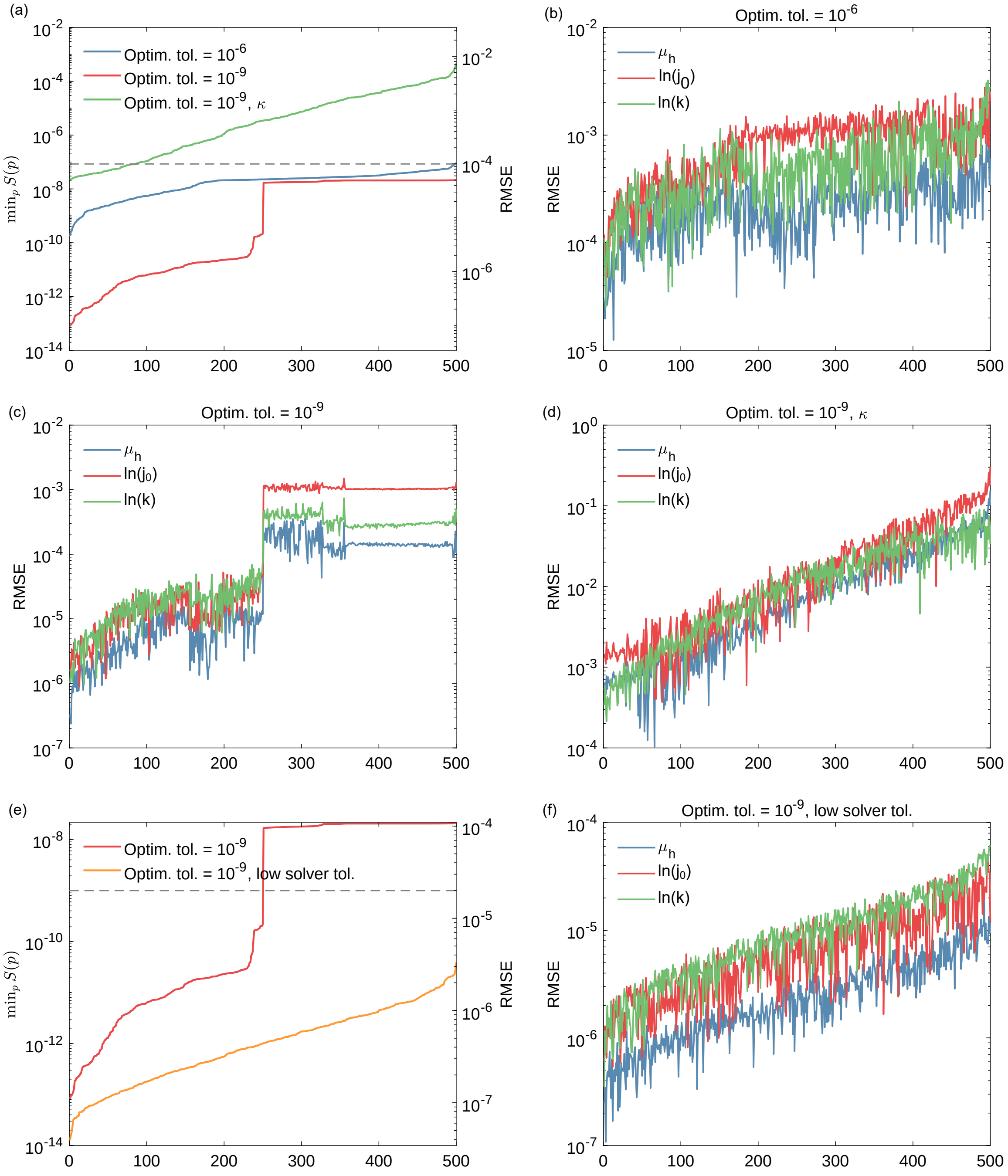

Figure S32: The optimization results starting from 500 randomly chosen initial guesses. (a) The sorted final objective function $S(\mathbf{p})$ and RMSE of $c(x)$ when the DAE solver relative and absolute tolerance is 10^{-3} and 10^{-4}. A comparison of high and low optimizer tolerance and when κ is or is not included as a free parameter. The dashed line corresponds to the absolute tolerance of the DAE solver. (b,c,d) the RMSE of $\mu_h(c)$, $j_0(c)$, and $k(\mathbf{x})$ that correspond to the sorted objective function in (a). (e) The sorted final objective function $S(\mathbf{p})$ and RMSE of $c(x)$ when the optimizer tolerance is 10^{-9}, a comparison of high and low solver tolerance. The dashed line corresponds to the optimizer tolerance. (f) the RMSE of $\mu_h(c)$, $j_0(c)$, and $k(\mathbf{x})$ that correspond to the sorted objective function in (e).

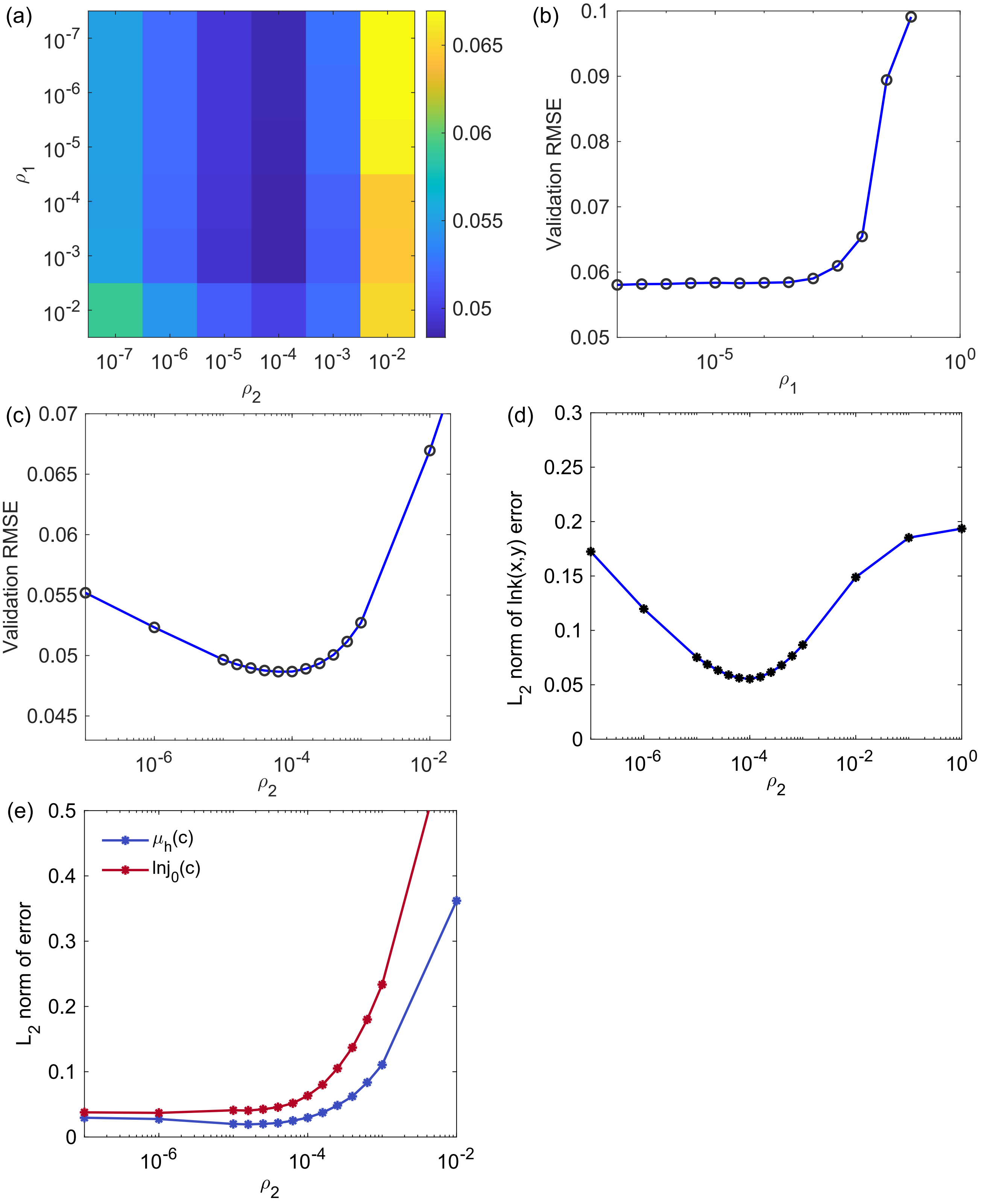

Figure S33: (a) The validation error as a function of ρ_1 and ρ_2. (b) The validation error as a function of ρ_1 when $\rho_2 = 0$. (c) The validation error as a function of ρ_2 when $\rho_1 = 0$. (d) L_2 norm of the error of $\ln k(\mathbf{x})$ as a function of ρ_2 when $\rho_1 = 0$. (e) L_2 norm of the error of $\mu_h(c)$ and $\ln j_0(c)$ as a function of ρ_2 when $\rho_1 = 0$.

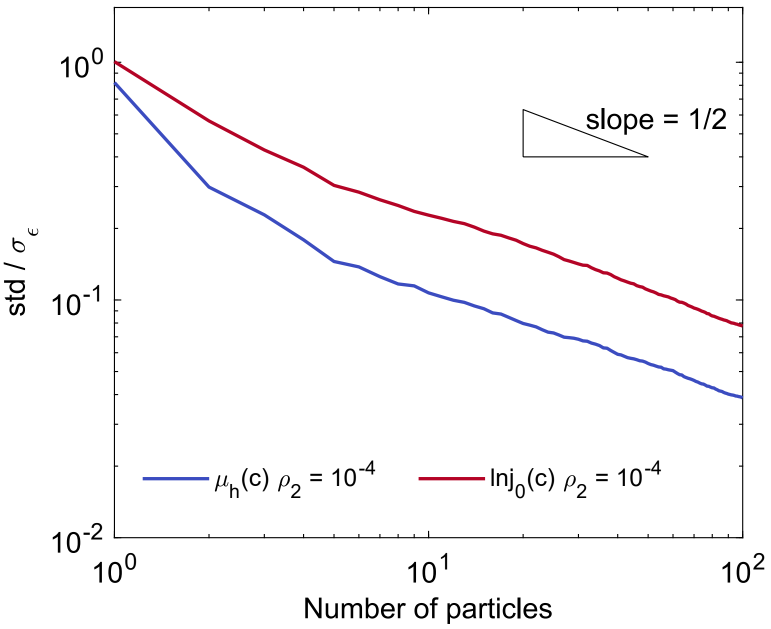

Figure S34: L_2 norm of the variance (std) of the MAP $\mu_h(c)$ and $\ln j_0(c)$ estimated using linear approximation as a function of the number of particles in the dataset for obtaining the MAP when $\rho_2 = 10^{-4}$

.

number of particles. The variance and bias are computed using linear approximation of the model as explained further in sec. 9.1. For the variance of the MAP $\mu_h(c)$ given the dataset (Eq. S134), we plot its L_2 norm (std) as a function of the number of particles in the dataset used to obtain the MAP when $\rho_2 = 10^{-4}$ in Fig. S34, and similarly for $\ln j_0(c)$. Note that the normalization condition on $\mathbf{Z}_i$ is imposed. We see that the square root of the variance of $\mu_h(c)$ and $\ln j_0(c)$ scales as $N^{-1/2}$, where N is the number of particles.

The bias of $\mu_h(c)$ is defined to be the L_2 norm of the deviation of the expectation of the MAP estimate $\mu_h(c)$ ($\mathbb{E}[\mu_h(c)]$) from the truth. Again, the expectation is estimated using the linear approximation of the model. The bias of $\ln j_0(c)$ is defined similarly. In Fig. S35a we plot the bias as a function of the number of particles in the dataset for obtaining the MAP with $\rho_2 = 10^{-4}$. We see that the bias decays toward a finite and nonzero value with an increasing number of particles. Fig. S35b shows that the bias increases with increasing regularization parameter ρ_2 when the number of particles is 50. Therefore, in summary, the bias of global parameters increases with increasing regularization of the spatial heterogeneity ρ_2, regardless of the number of particles. Note that this is the result of the heterogeneity being a multiplicative prefactor. In linear mixed model where the random effect is additive, unbiased estimator of the fixed effect (which corresponds to the global parameters in our nonlinear model) can be obtained regardless of the regularization on the random effect[74].

8.3 Sensitivity analysis of spatial heterogeneity

In choosing the prior for the spatial heterogeneity $\psi(\mathbf{x})$, we make the assumption that the same prior distribution for the random field $\psi(\mathbf{x})$ is shared by the entire ensemble of particles. This is because, for some particles, especially when the concentration field is nearly uniform, low-order components of $\psi(\mathbf{x})$ cannot be identified.

First, we illustrate the claim above by analyzing synthetic datasets generated using parameters in Section 5.1 with additive noise. we compute the squared error SE for different data sets as a function of Z_0, while all other parameters are fixed to be the truth.

Fig. S36 shows that when $Z_0 = 0$, at an average reaction rate of 0.3C, there is a distinct local minimum for SE at around $Z_0 = Z_{0,\mathrm{truth}}$. Note that noise is added to the synthetic dataset to this case so SE $\neq 0$ at $Z_0 = Z_{0,\mathrm{truth}}$.

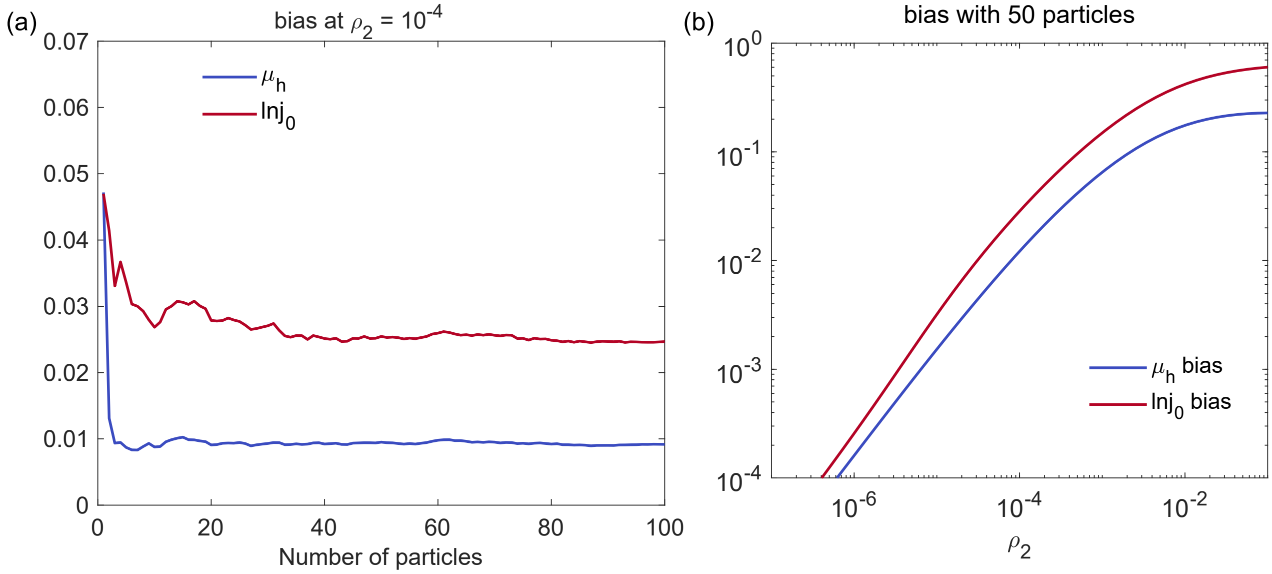

Figure S35: (a) The bias of $\mu_h(c)$ and $\ln j_0(c)$ as a function of the number of particles in the dataset for obtaining the MAP with $\rho_2 = 10^{-4}$. (b) The bias of $\mu_h(c)$ and $\ln j_0(c)$ as a function of ρ_2 when the number of particles is 50.

However, we notice that with a noiseless dataset with $Z_0 = -1$, at an average reaction rate of 2C, local minimum for SE at $Z_0 = Z_{0,\text{truth}}$ is very shallow, and when noise is added, local minimum no longer exists. Therefore, it is possible that the optimal value of Z_0 is unbounded.

Next, we show that this phenomenon is also observed with experimental datasets. Fig. S37 plots the squared error SE_i and $\text{SE}_i/2 + \rho_2 Z_0^2/2$ as a function of Z_0 of each particle around the MAP while all other parameters are fixed. We find that for some particles there exists a minimum squared error while for others the squared error is monotonic within the bound. Adding the regularization ensures that a local minimum exists and prevents Z_0 from becoming too large or small. The insensitivity may also be found in other low-order parameters. Therefore, it is important to impose a prior on the spatial heterogeneity shared by all particles.

8.4 Regularization of Z_0 and higher order parameters

As a result of the insensitivity with respect to Z_0, we ask the question: should we impose a different regularization coefficient on Z_0 and $Z_{i\neq 0}$? From a Bayesian perspective, the variance of the prior for Z_0 and $Z_{i\neq 0}$ is σ_{ψ_0} and σ_ψ, respectively, which may in general be different. Correspondingly, the regularization coefficient for Z_0 (ρ_{2,ψ_0}) and $Z_{i\neq 0}$ ($\rho_{2,\psi}$) can be different.

To answer this question, we vary ρ_{2,ψ_0} and $\rho_{2,\psi}$ independently. $j_0(c)$ and $\mu_h(c)$ are fixed at MAP when $\rho_{2,\psi_0} = \rho_{2,\psi} = 0.01$, hence optimization of $\mathbf{Z}_i$ of each particle can be done independently. The objective function

$$S_i(\mathbf{Z}_i) = \frac{1}{2}\text{SE}(\mathbf{Z}_i) + \frac{1}{2}\rho_{2,\psi_0} Z_{i,0}^2 + \frac{1}{2}\rho_{2,\psi} \sum_{j=1}^{N_{\text{KL}}} Z_{i,j}^2 \tag{S126}$$

is minimized, where $Z_{i,j}$ is the jth coefficient for particle i.

Particles with three episodes are used for the validation study. The first episode with the largest absolute C-rate is chosen for validation. Fig. S38 shows that while $\rho_{2,\psi}$ has the larger influence on the validation error, the validation error also shows a local minimum as a function of ρ_{2,ψ_0} at a given $\rho_{2,\psi}$. The minimum validation error is found at $\rho_{2,\psi} = 5$ and $\rho_{2,\psi_0} = 3$. In addition, increasing $\rho_{2,\psi}$ mostly decreases the intraparticle variance of $\psi(\mathbf{x})$, or V_2. Increasing ρ_{2,ψ_0} mostly decreases interparticle variation V_3. However, at small $\rho_{2,\psi}$, this reduction of V_3 is limited, because as ρ_{2,ψ_0} increases, $Z_{i\neq 0}$ can adjust to compensate for decreasing $|Z_0|$ such that the particle $\ln k$ mean barely changes, hence V_3 only decreases slightly.

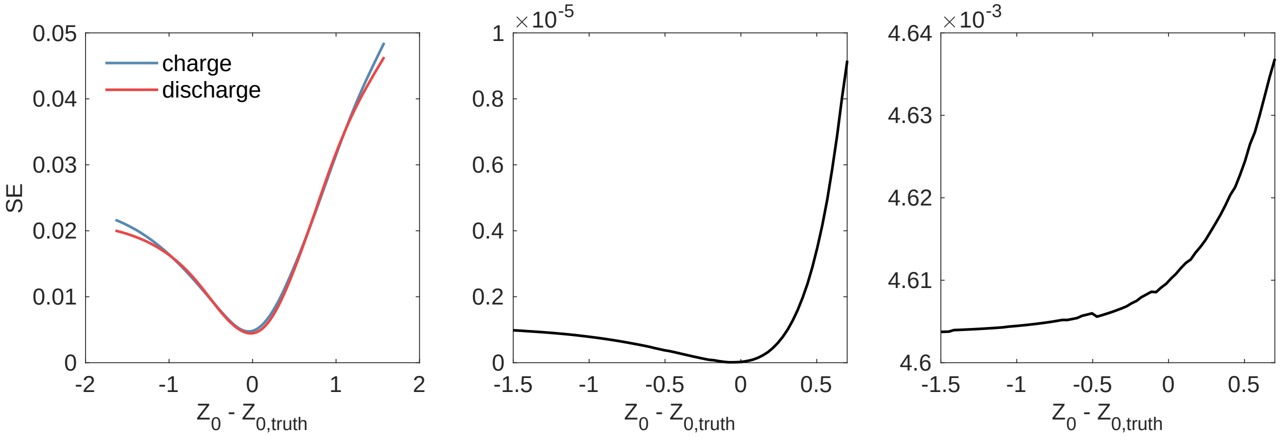

Figure S36: Squared error as a function of $Z_0 - Z_{0,\text{truth}}$ with all other parameters fixed. Datasets are generated with (a) $Z_{0,\text{truth}} = 0$, average reaction rate of 0.3C, with additive noise (b) $Z_{0,\text{truth}} = -1$, average reaction rate of 2C, without noise, (c) $Z_{0,\text{truth}} = -1$, average reaction rate of 2C, with additive noise.

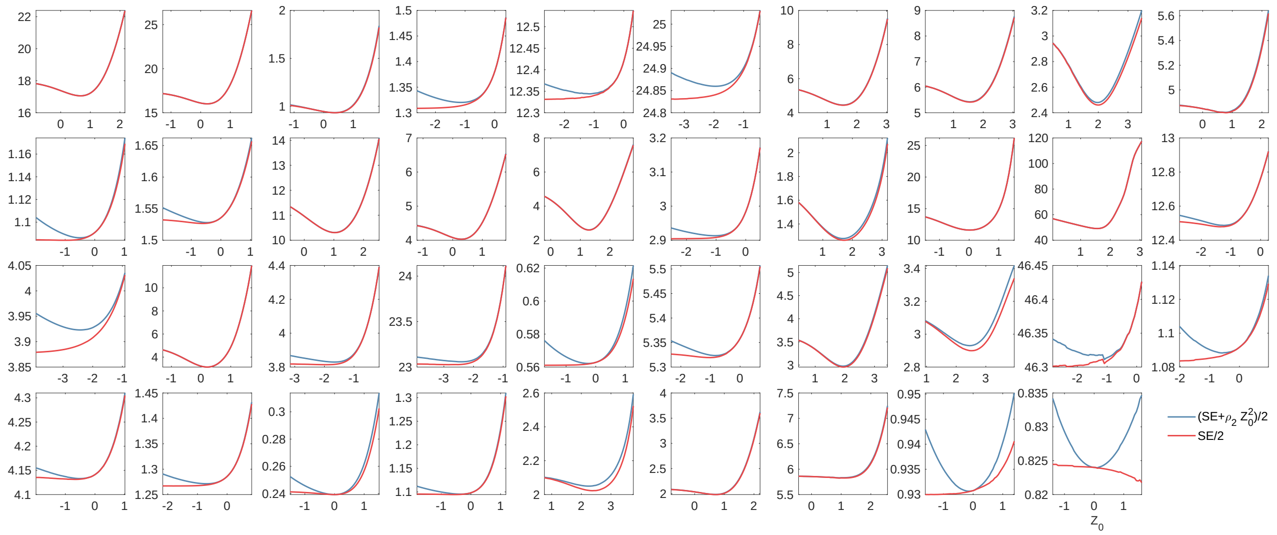

Figure S37: Squared error SE_i and $(\text{SE} + \rho_2 Z_0^2)/2$ of each particle (including all episodes) as a function of its respective Z_0 near the optimum when all other parameters are fixed.

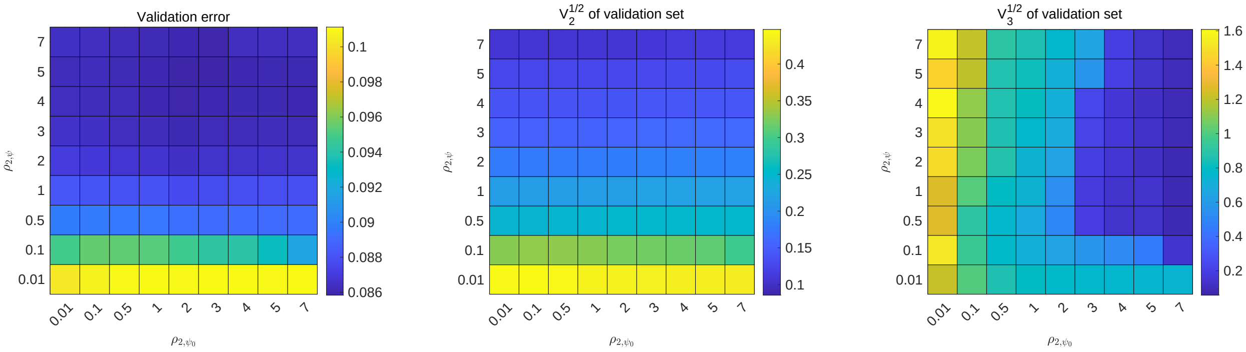

Figure S38: The validation error, intraparticle variation $V_2^{1/2}$, and interparticle variation $V_3^{1/2}$ of the validation dataset when ρ_{2,ψ_0} and $\rho_{2,\psi}$, the regularization coefficients on Z_0 and $Z_{i\neq 0}$ respectively, varies independently.

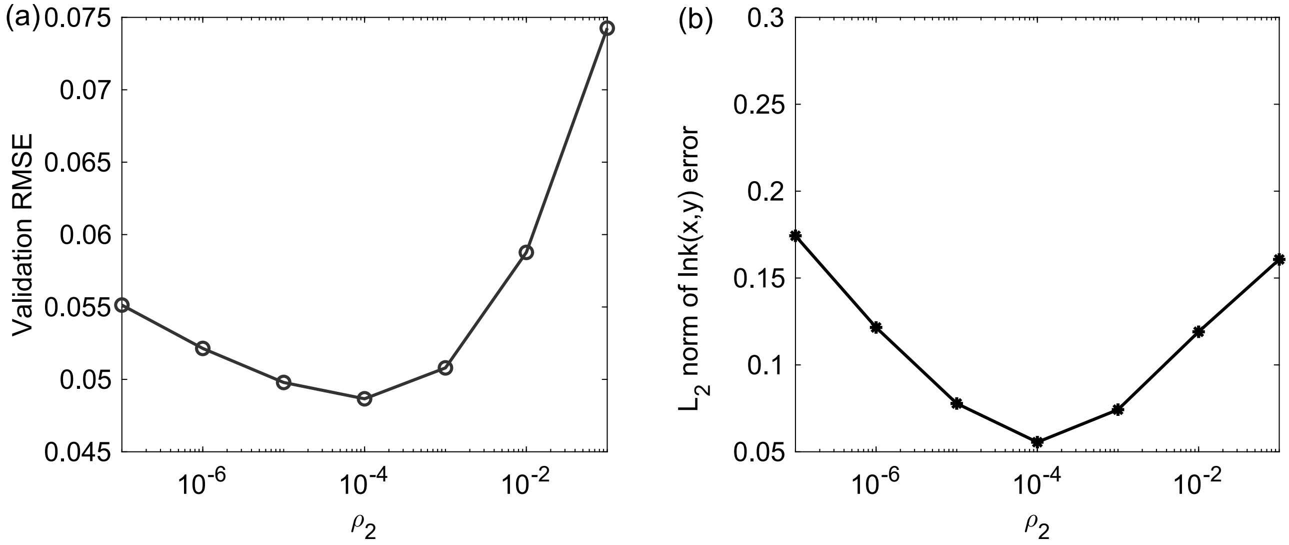

Figure S39: Fixing $\mu_h(c)$ and $j_0(c)$ to be the MAP solution at $\rho_2 = 0$: (a) the validation error as a function of ρ_2 when $\rho_1 = 0$. (b) L_2 norm of the error of $\ln k(\mathbf{x})$ as a function of ρ_2 when $\rho_1 = 0$.

Therefore, regularization on both Z_0 and $Z_{i\neq 0}$ is important and for simplicity, in the following text, we consider only when these two parameters are equal ($\rho_{2,\psi_0} = \rho_{2,\psi}$).

8.5 Method of regularization

Based on the results in sec. 8.1, in this section, we set $\rho_1 = 0$ to reduce the bias of the inferred $\mu_h(c)$ and $j_0(c)$. In 8.1, using synthetic datasets, we have shown that the reduction in the error of $\mu_h(c)$ and $j_0(c)$ with the use of regularization (ρ_2) is much smaller than that of $k(\mathbf{x})$ and it is expected to be even smaller with a larger number of particles. Notice that in Fig. S33(c-e), all parameters are updated when ρ_2 varies. Next, with the same synthetic datasets, we now fix $\mu_h(c)$ and $j_0(c)$ to be the MAP solution at $\rho_2 = 0$, then vary ρ_2, do the optimization with respect to $\mathbf{Z}_i$, and calculate the validation error. By comparing with Fig. S33(c,d), Fig. S39 shows that this alternative approach which fixes $\mu_h(c)$ and $j_0(c)$ results in a similar optimal ρ_2, a similar reduction of the validation error, and a similar reduction of the L_2 norm of the error of $\ln k(x, y)$ compared to allowing $\mu_h(c)$ and $j_0(c)$ to vary.

Therefore, this section compares these two methods of validation with experimental datasets. In the first

approach, $\mu_h(c)$ and $j_0(c)$ are free to change as ρ_2 changes, the optimization is

$$\min_{\mathbf{p}} S(\mathbf{p};\rho_2) = \frac{1}{2}\mathrm{SE}(\mathbf{p}) + \frac{\rho_2}{2}\sum_i \|\mathbf{Z}_i\|^2, \tag{S127}$$

where $\mathbf{p} = [\mathbf{p}_{\text{global}}, \mathbf{Z}_1, \ldots, \mathbf{Z}_N]$ and the normalization condition is imposed on the spatial heterogeneity. All particles are included in the optimization and must be evaluated in parallel.

In this section, we split the entire dataset into training and validation datasets: for particles with three episodes, the first episode with the largest absolute C-rate is chosen for validation, while all the other episodes and particles with less than three episodes are used for training.

The second approach is fixing the parameters of $\mu_h(c)$ and $j_0(c)$ when $\rho_2 = \sigma_\epsilon^2/\sigma_\psi^2 = 0.01$ is small to reduce bias of $\mu_h(c)$ and $j_0(c)$, that is $\mathbf{p}_{\text{global}}$ is the global parameters part of $\arg\min_{\mathbf{p}} S(\mathbf{p};\rho_2 = 0.01)$, where the objective function includes all datasets. As ρ_2 varies, since the global parameters are fixed, particle square error SE_i is only dependent on $\mathbf{Z}_i$, hence the following optimization problem is solved independently for each particle,

$$\min_{\mathbf{Z}_i} \frac{1}{2}\mathrm{SE}_i(\mathbf{p}_{\text{global}}, \mathbf{Z}_i) + \frac{\rho_2}{2}\|\mathbf{Z}_i\|^2. \tag{S128}$$

The same training and validation datasets are used for the second approach. Clearly, the normalization condition cannot be imposed on individual particles, and hence the constraint is removed. The normalization is re-applied after the optimization when $\ln k(\mathbf{x})$ is obtained for all particles.

Fig. S40(a-b) reports the training and validation RMSE. The training error increases with increasing ρ_2, while the validation error shows local minima for both approaches (note that the training and validation error does not include the regularization term). The training error when $\mu_h(c)$ and $j_0(c)$ are free is lower than when they are fixed since the optimization is performed with more degrees of freedom. In contrast, the validation error when $\mu_h(c)$ and $j_0(c)$ are free is higher than when they are fixed, suggesting that a larger error in $\mathbf{p}$ when global parameters are free during regularization coefficient sweep.

Fig. S40c shows that the two approaches produce a similar variance of the spatial heterogeneity, which decreases with increasing ρ_2, while the decrease in the interparticle variation is more significant than intraparticle variation, consistent with the insensitivity of the objective function with respect to Z_0. Fig. S40 also compares $\mu_h(c)$ and $j_0(c)$ at the optimal ρ_2 when the validation error is at its minimum.

Since fixing $\mu_h(c)$ and $j_0(c)$ when performing a sweep of the regularization coefficient yields a smaller validation error, we will use this method in our subsequent discussion and the main text.

8.6 k-fold cross-validation

In the previous validation, we chose a particular partition of training and validation datasets. In this section, we explore different combinations and perform k-fold cross-validation. Since $\mu_h(c)$ and $j_0(c)$ are fixed during the sweep of the regularization coefficient, particles can be simulated, optimized with respect to their spatial heterogeneity, and evaluated on the validation set independently.

Within particles that have three episodes, we denote $\mathrm{SE}_{i,j}$ to be the squared error of the jth episode when it is used as the validation set of particle i while using other episodes of the particle for training. 3-fold cross-validation can be performed, yielding validation error for $j = 1, 2, 3$. Suppose episode $\mathcal{V}(i)$ is chosen for validation for particle i, we define the validation MSE,

$$\mathrm{MSE}_{\mathcal{V}} = \frac{\sum_i \mathrm{SE}_{i,\mathcal{V}(i)}}{\sum_i A_i(N_{T,i,\mathcal{V}(i)} - 1)}, \tag{S129}$$

where the summation is over particles with three episodes, $\mathcal{V}(i) = 1, 2,$ or 3, A_i is the number of pixels in particle i, and $N_{T,i,j}$ is the number of images in the jth episode of particle i. We compute the MSE for all possible $\mathcal{V}$ (3 raised to the power of the number of particles with three episodes, or 3^6 possibilities in our study).

We define the sample mean $\overline{\mathrm{MSE}_{\mathcal{V}}}$ and sample variance $s^2(\mathrm{MSE}_{\mathcal{V}})$ based on all combinations. Fig. S41 plots $\overline{\mathrm{MSE}_{\mathcal{V}}} \pm s(\mathrm{MSE}_{\mathcal{V}})$ as a function of the regularization coefficient ρ_2. Since the validation error is estimated with

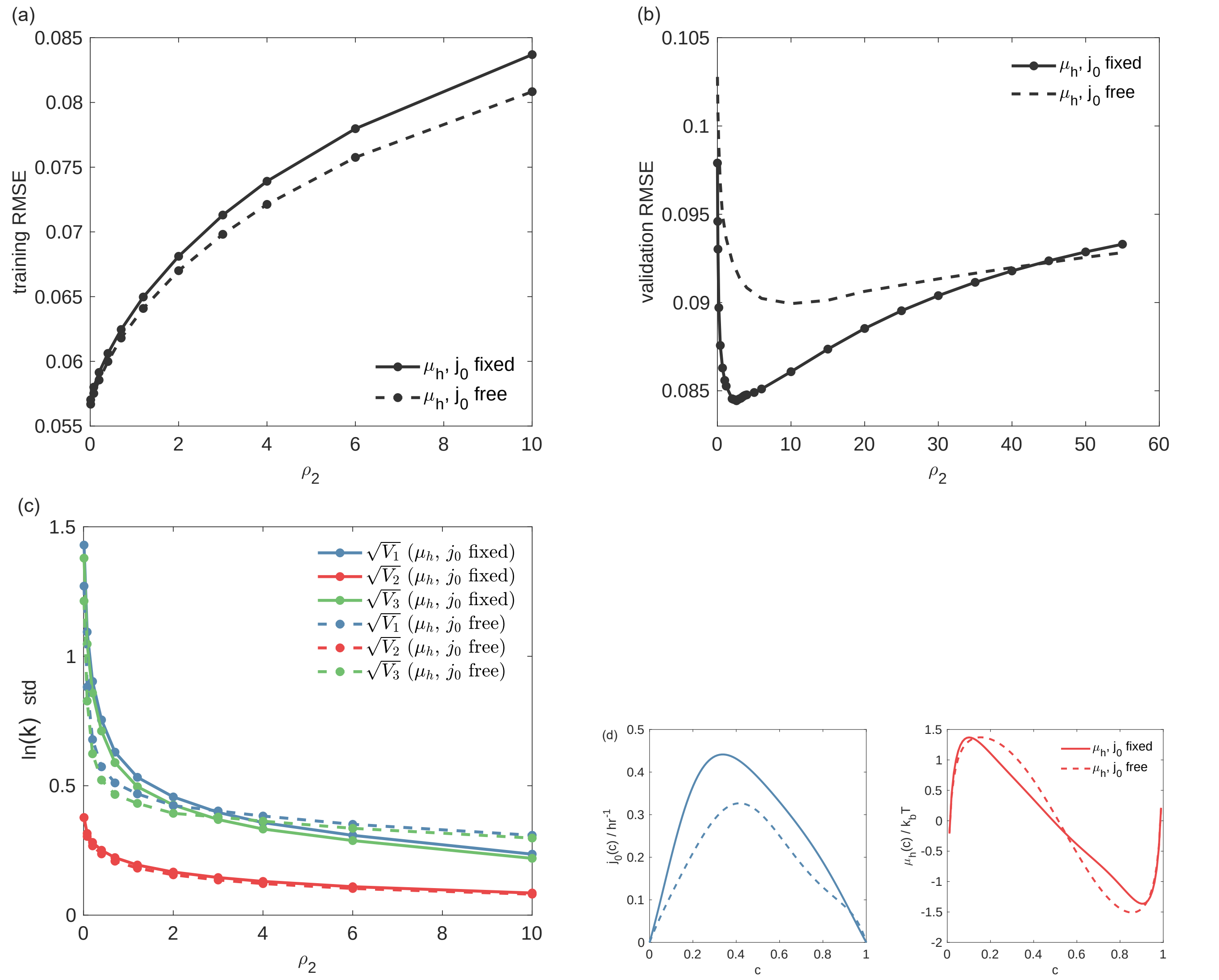

Figure S40: A comparison of (a) training RMSE, (b) validation RMSE, and (c) $\ln k$ statistics as a function of the regularization coefficient ρ_2 when $\mu_h(c)$ and $j_0(c)$ are free to change and when they are fixed during the ρ_2 sweep. d) a comparison of $\mu_h(c)$ and $j_0(c)$ fixed at the MAP and $\rho_2 = 0.01$ with all data included ($j_0(c)$ rescaled so that the normalization condition is satisfied at the optimal ρ_2), and $\mu_h(c)$ and $j_0(c)$ that corresponds to the minimum validation error when they are free to change.

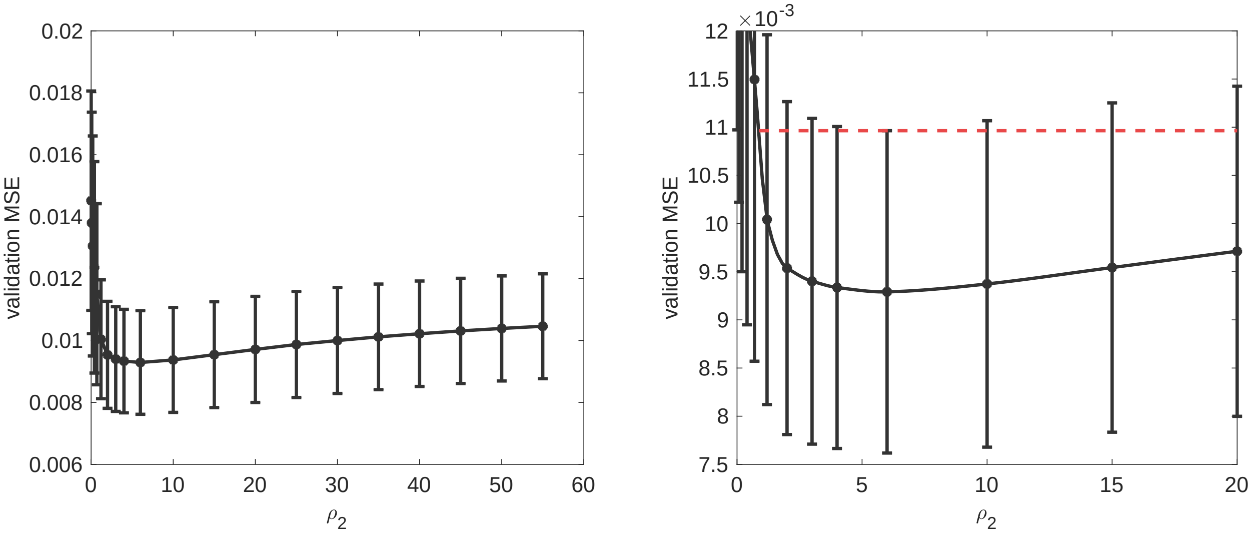

Figure S41: The sample mean and standard deviation of the validation mean squared error $\mathrm{MSE}_{\mathcal{V}}$ as a function of the regularization coefficient ρ_2. The right plot is a zoom-in view of the left plot.

uncertainty, instead of choosing ρ_2 with the smallest mean validation error, we can use the one-standard-error rule, choosing the smallest ρ_2 that is one standard deviation above the minimum validation error [54], which is $\rho_2 = 0.88$.

We can evaluate the performance of the model by analyzing the result of the 3-fold cross-validation. Fig. S42 shows the validation error curve for each particle, including the validation RMSE of each of the 3-fold validation of the particle $\mathrm{RMSE}_{i,j}$ (jth episode is the validation dataset), and the average validation RMSE of each particle, defined by the square root of $\frac{\sum_j \mathrm{SE}_{i,j}}{\sum_j A_i N_{T,i,j}}$, where $\mathrm{SE}_{i,j}$ is the squared error of the validation error of particle i using the jth episode for validation.

The average validation errors of particles 1, 2, 3, 5, and 6 show clearly defined local minimum validation errors. The corresponding optimal ρ_2 are 15, 0.7, 2, 6, and 0.7. The validation error curves of particle 4 and $\mathrm{RMSE}_{1,1}$ of particle 1 do not have a well-defined local minimum and decrease with increasingly large ρ_2 and approaches the asymptotic limit when $\rho_2 \to \infty$, which is indicated by the dashed lines and corresponds to when there is no spatial heterogeneity $\ln k(\mathbf{x}) = 0$. This suggests the model is not generalizable to all episodes of particle 4 and episode 1 of particle 1, in other words, there exist some particles and episodes in the dataset whose dynamics the current model does not fully account for.

By examining the centered $\ln k(\mathbf{x})$ (subtracted by its mean) from each of the 3-fold cross-validation (Fig. S43) at the same regularization coefficient ($\rho_2 = 0.88$), we find that particles 2, 3, 5, and 6 show strong pointwise correlation among all three $\ln k(\mathbf{x})$. The consistency of $\ln k(\mathbf{x})$ indicates that the physical model can explain the data under different charge or discharge rates, therefore, all three validation errors increase at large enough ρ_2.

The other way of evaluating the validation error is to take the average MSE of all particles and k-fold validation tests, or the weighted squared error,

$$\mathrm{WSE} = \sum_{ij} \frac{\mathrm{SE}_{i,j}}{A_i N_{T,i,j}}. \tag{S130}$$

We exclude validation error curves that decrease monotonically in the range of ρ_2 studied and give all variance curves the same weight and compute the WSE. In addition to the 3-fold validation that we performed earlier on particles with 3 episodes, we also do 2-fold validation on particles with 2 episodes, that is, one is used as the training set and the other is the validation set, and vice versa. In Fig. S44, we find that 3-fold and 2-fold minimum validation WSE is found at $\rho_2 = 2$. Comparing this optimal ρ_2 with optimal $\rho_2 = 0.88$ determined previously, we choose the lower value $\rho_2 = 0.88$ since $\rho_2 = 0.88$ produces a validation error in Fig. S44 that is only slightly larger than $\rho_2 = 2$.

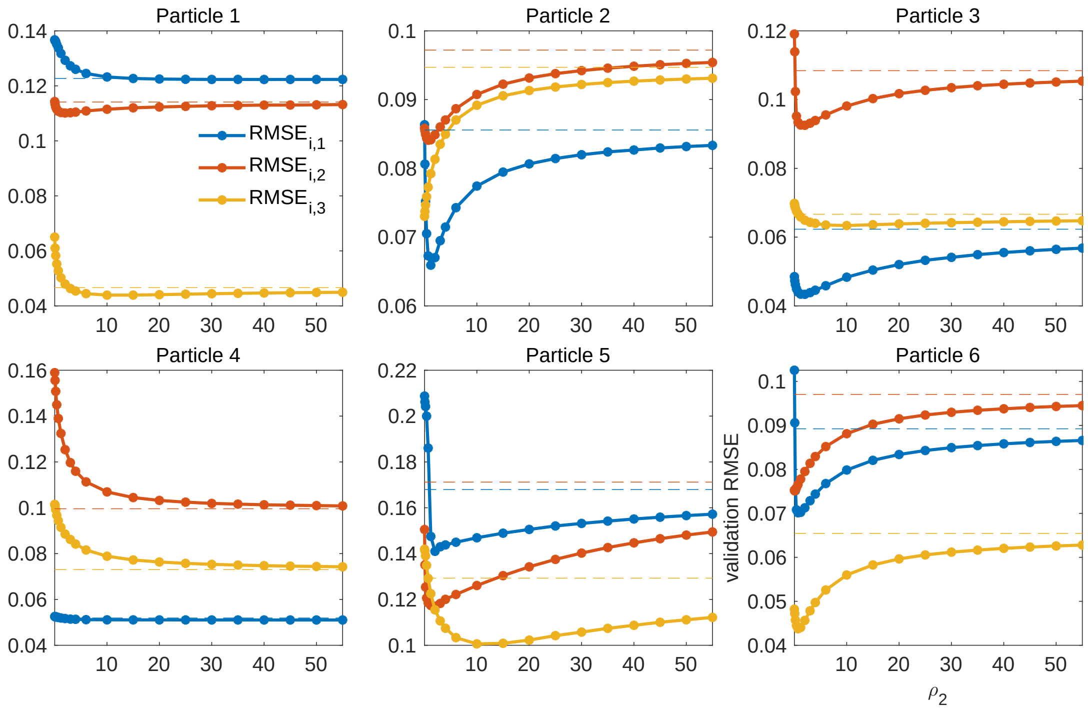

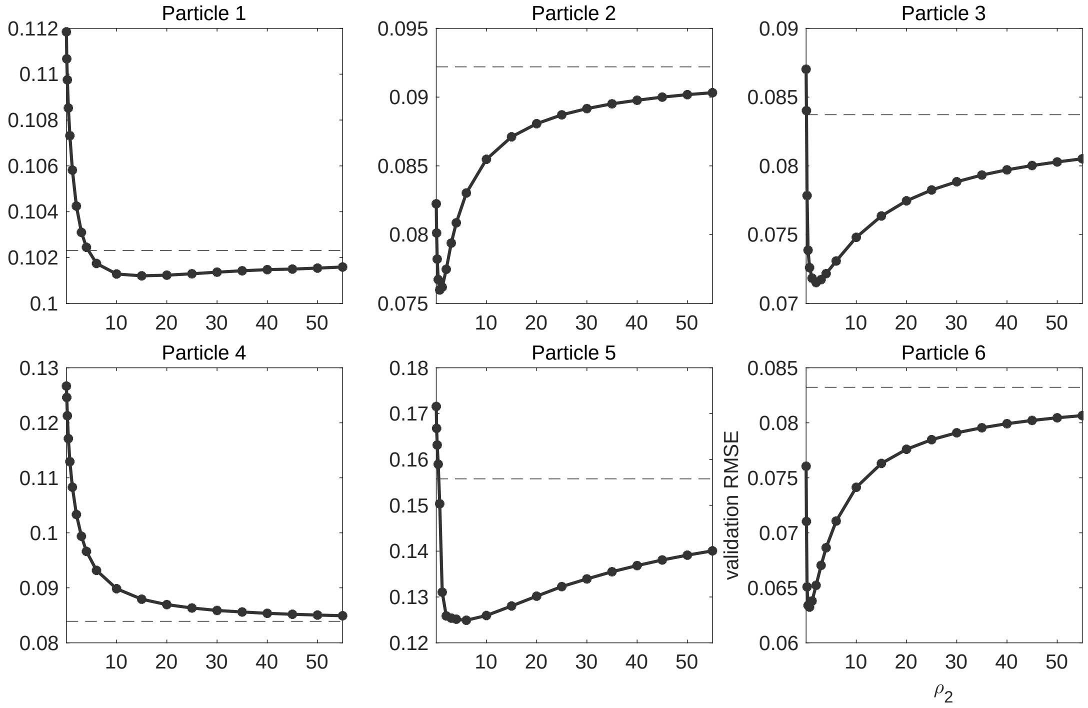

Figure S42: Top: the RMSE of each of the 3-fold validation of the particle $\mathrm{RMSE}_{i,j}$, where $i = 1, \ldots, 6$, corresponding to particle 1 to 6. Bottom: average validation RMSE of each particle RMSE_i. The dashed lines are the asymptotic limit when $\rho_2 = \infty$, that is, when there is no spatial heterogeneity ($\ln k(\mathbf{x}) = 0$).

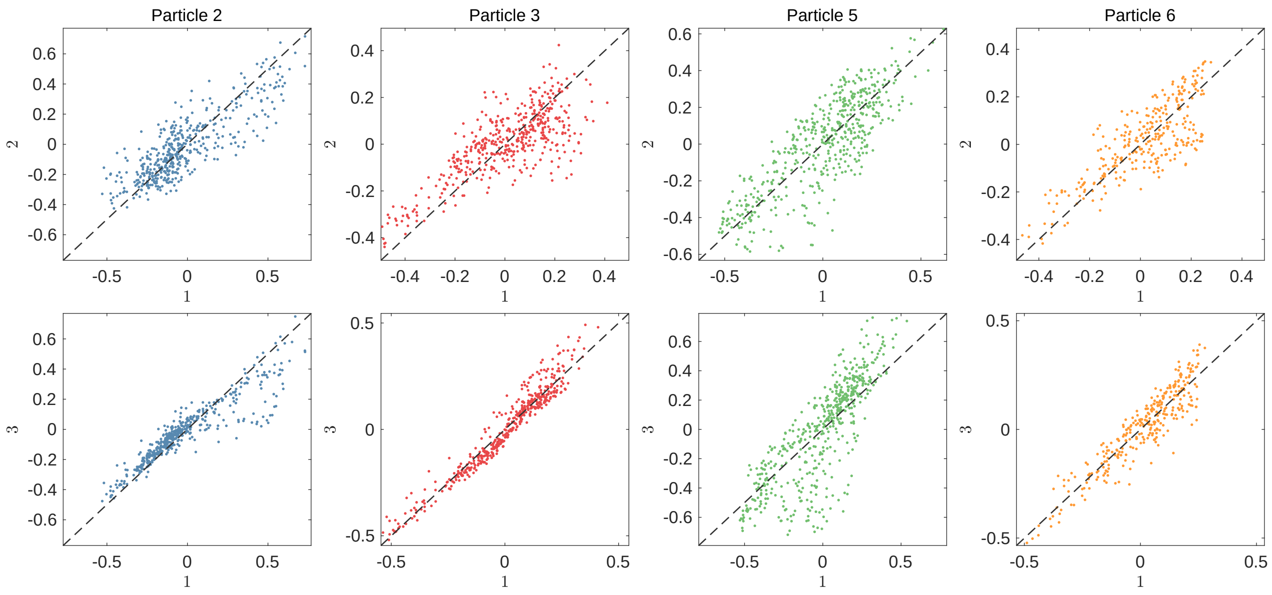

Figure S43: Pointwise correlation plots of centered $\ln k(\mathbf{x})$ from the k-fold validation for particles 2, 3, 5, and 6 when $\rho_2 = 0.7$. The horizontal and vertical axis labels refer to which dataset is used for validation. The other two datasets are then used for training.

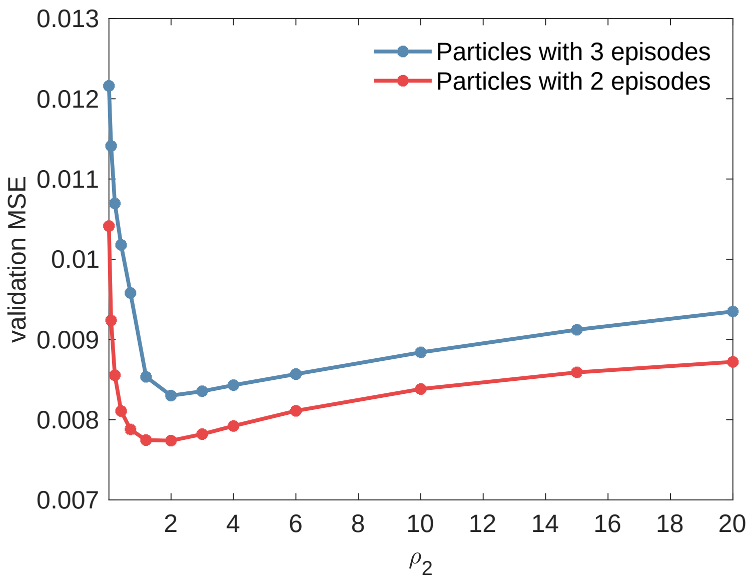

Figure S44: The validation WSE for particles with 3 episodes and 2 episodes as a function of ρ_2.

9 Validation

9.1 Uncertainty quantification

This section considers three methods of uncertainty quantification: linear approximation, Hamiltonian Monte Carlo, and bootstrapping.

Based on the discussion in Section 8.5 and Bayesian inference, the corresponding posterior distribution is

$$P(\mathbf{p}|c_{\text{data}}) \propto \exp -\frac{1}{2}\left[\sum_{i,j}\frac{1}{\sigma_\epsilon^2}\|c_{i,j}(\mathbf{p}_{\text{global}},\mathbf{Z}_i)-c_{\text{data},i,j}\|_2^2+\frac{1}{\sigma_\psi^2}\sum_i\|\mathbf{Z}_i\|^2\right]=\exp -\left(\frac{S(\mathbf{p})}{\sigma_{\text{obs}}^2}\right), \tag{S131}$$

since we set $\rho_1 = 0$.

Linear approximation expands the model linearly at the MAP, hence

$$P(\mathbf{p}|c_{\text{data}}) \propto \exp -\frac{1}{2}\left[\mathbf{p}^*\mathbf{H}\mathbf{p}+\frac{1}{\sigma_\psi^2}\sum_i\|\mathbf{Z}_i\|^2\right], \tag{S132}$$

where

$$\mathbf{H}=\frac{1}{\sigma_\epsilon^2}\sum_{i,j}\left(\frac{\partial c_{i,j}}{\partial \mathbf{p}}\right)^*\frac{\partial c_{i,j}}{\partial \mathbf{p}}=\exp -\frac{1}{2}\mathbf{p}^*\Sigma\mathbf{p}. \tag{S133}$$

$\mathbf{H}$ is evaluated at $\mathbf{p}_{\text{MAP}}$, and Σ is the estimated covariance. In addition, we also impose the normalization constraint on the spatial heterogeneity (Section 4.1); therefore, the posterior distribution is conditional on the constraint (parameters are projected onto the null space of the constraint [32]). Under the linear approximation and given a linear constraint, the posterior distribution of the parameters is Gaussian. We report the marginal distribution of the functions. Due to the representation of $\mu_h(c)$ (Eq. S26),

$$\mathbb{V}\text{ar}[\mu_h(c)]=\sum_{ij}P_i(c)P_j(c)\mathbb{C}\text{ov}(a_i,a_j), \tag{S134}$$

where the covariance is taken over the posterior distribution, and similarly for $\ln j_0(c)$. Recall from the previous discussion that the magnitude of $k(\mathbf{x})$ and $j_0(c)$ has a large uncertainty in this study. Therefore we aim to quantify the uncertainty of intraparticle variation $\psi(\mathbf{x})-\bar{\psi}$. Given Eq. S67, and define the mean of the basis functions $\bar{\phi}_i=\int\phi_i(x)dx/A$, where $A=\int dx$ is the area of the particle, then the marginal posterior mean and variance is

$$\mathbb{E}\left[\psi(\mathbf{x})-\bar{\psi}\right]=\sum_{i=1}^{N_{\text{KL}}}\sqrt{\lambda_i}(\phi_i(x)-\bar{\phi}_i)\mathbb{E}[Z_i] \tag{S135}$$

$$\mathbb{V}\text{ar}\left[\psi(\mathbf{x})-\bar{\psi}\right]=\sum_{i,j=1}^{N_{\text{KL}}}\sqrt{\lambda_i\lambda_j}(\phi_i(x)-\bar{\phi}_i)(\phi_j(x)-\bar{\phi}_j)\mathbb{C}\text{ov}(Z_i,Z_j), \tag{S136}$$

where the expectation and covariance of Z_i are taken over the posterior distribution. We are also interested in quantifying the averaged variance over space,

$$\frac{1}{A}\int\mathbb{V}\text{ar}\left[\psi(\mathbf{x})-\bar{\psi}\right]dx=\sum_i\lambda_i\mathbb{V}\text{ar}[Z_i]-\sum_{ij}\sqrt{\lambda_i\lambda_j}\bar{\psi}_i\bar{\psi}_j\mathbb{C}\text{ov}(Z_i,Z_j). \tag{S137}$$

The linear approximation is very fast because only FSA needs to be performed but the uncertainty is only a very crude estimate. Hamiltonian Monte Carlo (HMC) is commonly used to sample posterior distribution, especially when model evaluation is computationally expensive. For HMC, models and ASA are evaluated on all particles and episodes in parallel at each step. ASA computes the gradient of $S(p)$ efficiently. However, the particles have different sizes and hence the evaluation time varies widely; a small subset of particles and episodes takes significantly longer time than others, creating a bottleneck effect while most other workers become idle. Therefore, we rank the

particles and episodes by their model evaluation time and choose 21 particles (30 episodes in total) that are faster to evaluate, ensuring efficient use of all workers by reducing their idle time so that the Monte Carlo sampling can be achieved in a reasonable amount of time. In total, we obtained a chain of 53,000 samples. Normalization constraint is imposed. The parameter space is transformed based on Cholesky decomposition of the Hessian at the MAP. The preconditioning leads to significantly more efficient chain mixing.

The MAP solution at $\rho_2 = 0.01$ corresponds to the previously estimated variance of the prior for $\psi(\mathbf{x})$ at $\sigma_\psi = 0.7$. Fig. S45a shows the 99% confidence interval of $\mu_h(c)$, $j_0(c)$, and $j_0(c)/j_{0,\max}$ from a HMC of 5.3×10^5 samples on the subset of particles. We also show the standard deviation of the centered spatial heterogeneity $(\psi(\mathbf{x}) - \bar{\psi})$ for each particle (Fig. S45c). HMC results are then compared with the uncertainty and posterior mean estimated using linear approximation based on the same subset of particles (Fig. S45b,d). The magnitude of the uncertainty of intra-particle variation is well captured by the linear approximation. Linear approximation underestimates the uncertainty of $j_0(c)$ compared to the HMC result.

The average standard deviation of $\ln k(\mathbf{x})$ over all particles,

$$\left(\frac{\sum_i \int_i \mathbb{V}\mathrm{ar}\left[\psi(\mathbf{x}) - \bar{\psi}\right] dx}{\sum_i A_i}\right)^{1/2}, \tag{S138}$$

is 0.24, which is much larger than the variation due to the difference in boundary condition or mesh size, justifying the choice of boundary condition and mesh size made in Sections 6.9 and 6.5.

There exists a small difference between the posterior mean of $\mu_h(c)$ and $j_0(c)$ based on a subset of particles from HMC and the MAP based on the entire dataset shown in Fig. S40, which is due to the presence of particles and episodes that are not completely accounted for by the model. Hence the heterogeneity of the dataset may also contribute to the uncertainty of the inferred models. For this reason, we next use bootstrapping to estimate the uncertainty of the MAP estimators for $\mu_h(c)$ and $j_0(c)$.

Specifically, we sample N_b times from all particles ($N_b = 470$) with replacement. Images from different particles can be assumed to be independent and hence the noise of each particle can be assumed to satisfy the iid condition for bootstrapping. For each sample, optimization with regularization ($\rho_2 = 0.01$) is performed to find the MAP. Here we do not use optimal ρ_2 because we are interested in estimating the uncertainty of $\mu_h(c)$ and $j_0(c)$ and in previous discussions, their MAPs are obtained at $\rho_2 = 0.01$. Some particles may be drawn n times and hence their squared errors are multiplied by n in the objective function. Based on the bootstrapping method, the ensemble of MAP serves as an estimation of the uncertainty of $\mu_h(c)$ and $j_0(c)$ [33]. Fig. S46a shows the MAP of all samples. Fig. S46b shows the 99% confidence interval from this ensemble of MAP results. We notice that there is a significant spread in the magnitude of $j_0(c)$ due to the insensitivity to its magnitude as explained above but the normalized $j_0(c)/j_{0,\max}$ is consistent among different samples. Fig. S46c shows the histogram of the training RMSE.

We remark that the uncertainty of $\mu_h(c)$ and $j_0(c)$ is reduced significantly compared to those inferred based on the uniformity coefficient (Fig. S14).

9.2 Comparison of spatial heterogeneity estimated using finite difference and inversion

We relax the criteria used in Section 4.2 to find pairs of images eligible for finite difference (FD) estimate of $\psi(\mathbf{x}) - \bar{\psi}$ in order to have a particle whose all three episodes are eligible for the estimate. The second criterion is relaxed to: the larger variance (V_2) of the two frames must be smaller than 0.04.

In Fig. S47a, we highlight pairs chosen for FD estimates by coloring frame edges with the same color. FD estimates for $\psi(\mathbf{x}) - \bar{\psi}$ are plotted next to the images. The scatter plots for all three pairs are shown in Fig. S47b. We find that the correlation between estimates based on the three episodes is poor. The range of $\ln k(\mathbf{x})$ from the second episode is much larger than the other two.

$\psi(\mathbf{x}) - \bar{\psi}$ from the full inversion using an optimal regularization parameter $\rho_2 = 0.88$ are also shown in Fig. S47a next to the FD estimate. The label indicates which dataset is used for training. For example, (1,2) means trained on episodes 1 and 2. Comparing the FD estimate and inversion result, we find that the pattern and magnitude of spatial

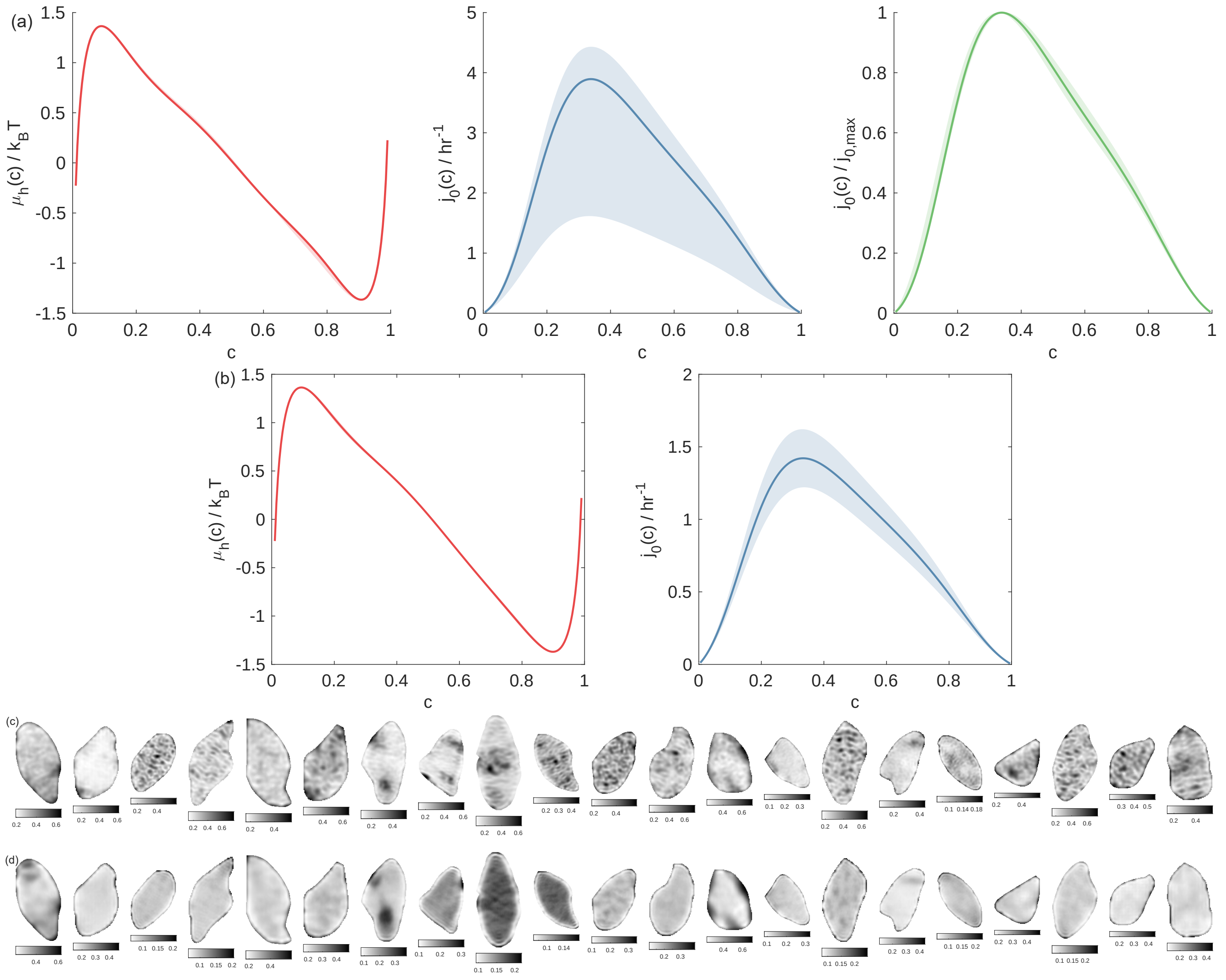

Figure S45: (a) 99% confidence interval and posterior mean of $\mu_h(c)$, $j_0(c)$, and $j_0(c)/j_{0,\max}$ from Hamiltonian Monte Carlo. (b) Estimated uncertainty based on linear approximation. 99% confidence interval of $\ln k(\mathbf{x}) - \overline{\ln k(\mathbf{x})}$ estimated using HMC (c) and linear approximation (d).

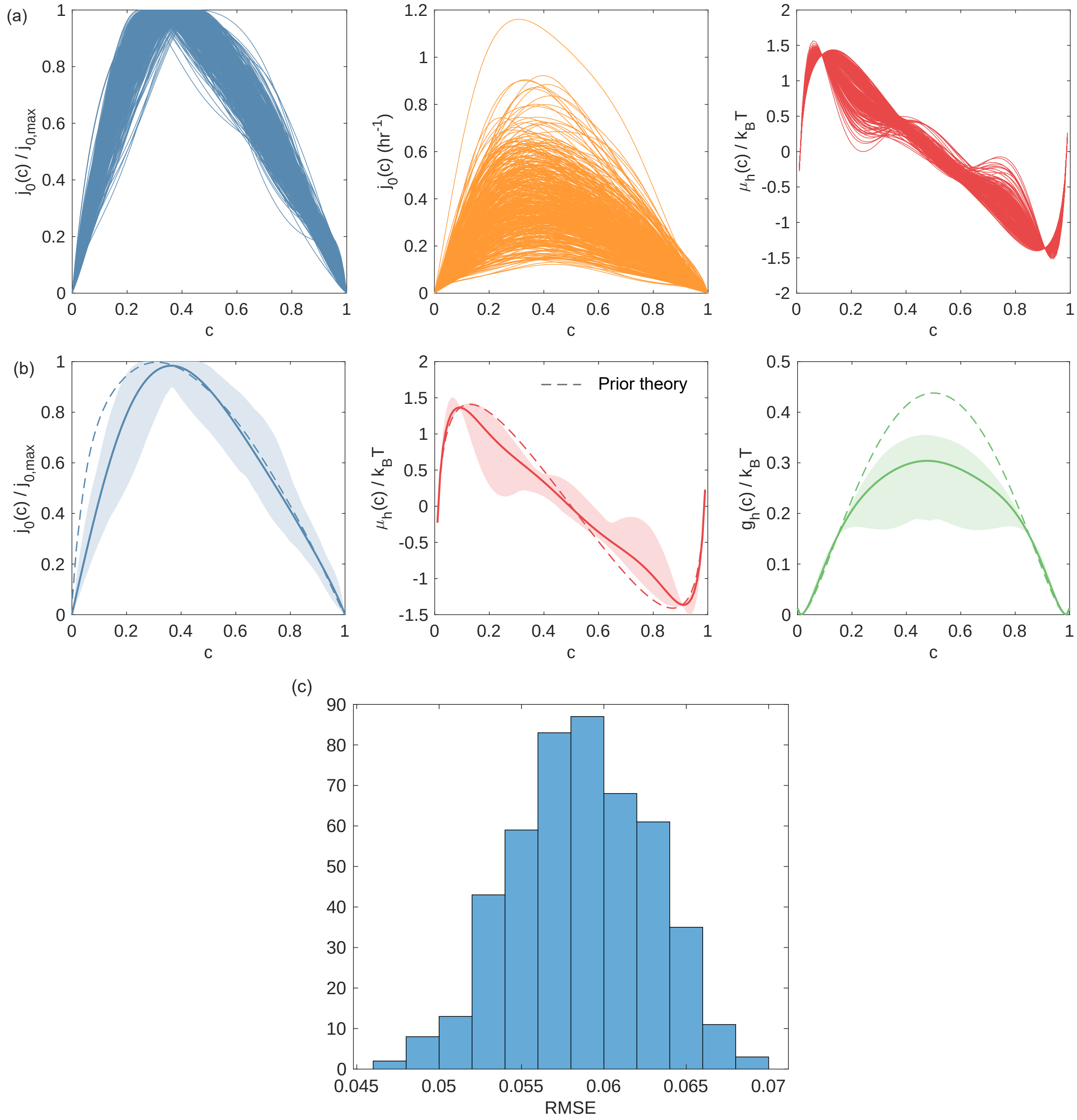

Figure S46: (a) MAP of all bootstrapping samples. (b) the mean (solid curves) and the 99% confidence interval (shaded region) of normalized exchange current $j_0(c)/j_{0,\max}$, homogeneous chemical potential and corresponding free energy $g_h(c) = d\mu_h(c)/dc$. (c) the training RMSE at the MAP for all samples.

heterogeneity revealed from the FD estimate of episode 1 is mostly consistent with inversion, except in a small region near the left boundary. In comparison with the poor correlation of the FD estimates, We find remarkably good correlation and match between each pair of $\psi(\mathbf{x}) - \bar{\psi}$ from the full inversion using different training datasets (Fig. S47c,d).

These results suggest that PDE-constrained optimization and regularization may be needed to obtain a solution to the spatial heterogeneity that is consistent across different episodes.

Fig. S48 shows an example of a particle with 2 episodes. We see a significant improvement in the correlation between the $\psi(\mathbf{x}) - \bar{\psi}$ trained on two different episodes using full inversion than using FD. The optimization reveals details of $\ln k(\mathbf{x})$ that must rely on the entire sequence, which the FD estimate cannot capture due to nonlinearity and the constraints on the frames that can be chosen. For example, the full inversion based on either episode can reveal the two small kinetically fast regions in the left half of the particle, while FD misses that. FD also fails to identify the right half of the particle as a kinetically fast region and only identifies the isolated island in the middle.

9.3 Comparison with auger electron microscopy (AEM)

The model assumes the rate of change of the local composition is $k(\mathbf{x})$ multiplied by the local surface reaction rate given by the Butler-Volmer kinetics. Because the local Li fraction c is depth-averaged, the spatial heterogeneity comes from the nonuniform thickness of the particle and the heterogeneity in the surface reaction rate. Based on the depth-averaged model and the reaction that happens on the top and bottom surface of the LFP nanoparticles, the spatial heterogeneity is

$$k(\mathbf{x}) = \frac{j_s(\mathbf{x})}{h(\mathbf{x})} \tag{S139}$$

where $j_s(\mathbf{x})$ is the kinetic prefactor for the surface reaction. A factor of 2 is absorbed into $h(\mathbf{x})$ because we can only measure its relative magnitude. The particle thickness is proportional to the optical density $\mathrm{OD}(x)$ from the STXM and we average the optical density over all snapshots and define nondimensionalized thickness $h(\mathbf{x}) = \mathrm{OD}(x)/\overline{\mathrm{OD}}$ where $\overline{\mathrm{OD}}$ is the spatially averaged optical density of the particle.

As explained in sec. 1.1, the LFP particles are coated with carbon which may be nonuniform due to particle-to-particle contact and local gas flow during the coating process. We confirm this with AEM which measures the signal from surface carbon and reveals that the carbon distribution on the surface is indeed heterogeneous. Next, we compare the inverted $j_s(\mathbf{x})$ with AEM carbon signal, in order to understand the origin of the spatial heterogeneity.

It is known in the literature that while pristine LFP without carbon coating has poor rate capability due to low electron conductivity, a carbon coating that is too thick also reduces the reversible capacity due to hindered electrolyte penetration and increased resistance in Li^+ transport between the electrolyte and LFP as revealed by the charge transfer resistance[43, 44, 45, 46, 75], therefore, there exists an optimal carbon coating thickness for the electrochemical performance, which, depending on the coating method and precursor, is around 1-2 nm [43], 5 nm [44], 4.2 nm [45]. In these studies, the authors found that the rate capabilities decrease when the thickness is 2-3, 8nm, and 14nm, respectively. Other studies also found that a small amount of carbon can increase the rate capability significantly [46, 75]. The results suggest that the kinetics of Li insertion and de-insertion is very sensitive to carbon coating thickness. Therefore, we compare $j_s(\mathbf{x})$ with the AEM carbon images as shown in Fig. 3b in the main text. We see that the "islands" of kinetically fast regions correspond well to the region with thin carbon coating. Fig. 3c plots the pixel-wise comparison of the values of $j_s(\mathbf{x})$ and AEM carbon image intensity after being centered (shifted by their respective mean) and scaled by their respective standard deviation $j_s(\mathbf{x})$ (also known as the z-score). The correlation coefficient is -0.4. The results show that the rate heterogeneity is well correlated with carbon thickness and thicker carbon coating slows down the reaction, suggesting our observed carbon coating thickness is likely greater than the optimal thickness for rate capability.

Here, we note that Ref. [76] found that in large LFP particles ($1-5\mu$m or larger), the carbon coating process using various carbon precursors leads to the formation of a secondary lithium deficient phase $Fe_2P_2O_7$, which is an inactive material. A nonuniform distribution of such a secondary phase on the LFP particle surface (which is observed in Ref. [76]) may also lead to heterogeneity in reaction kinetics. However, the authors have shown that

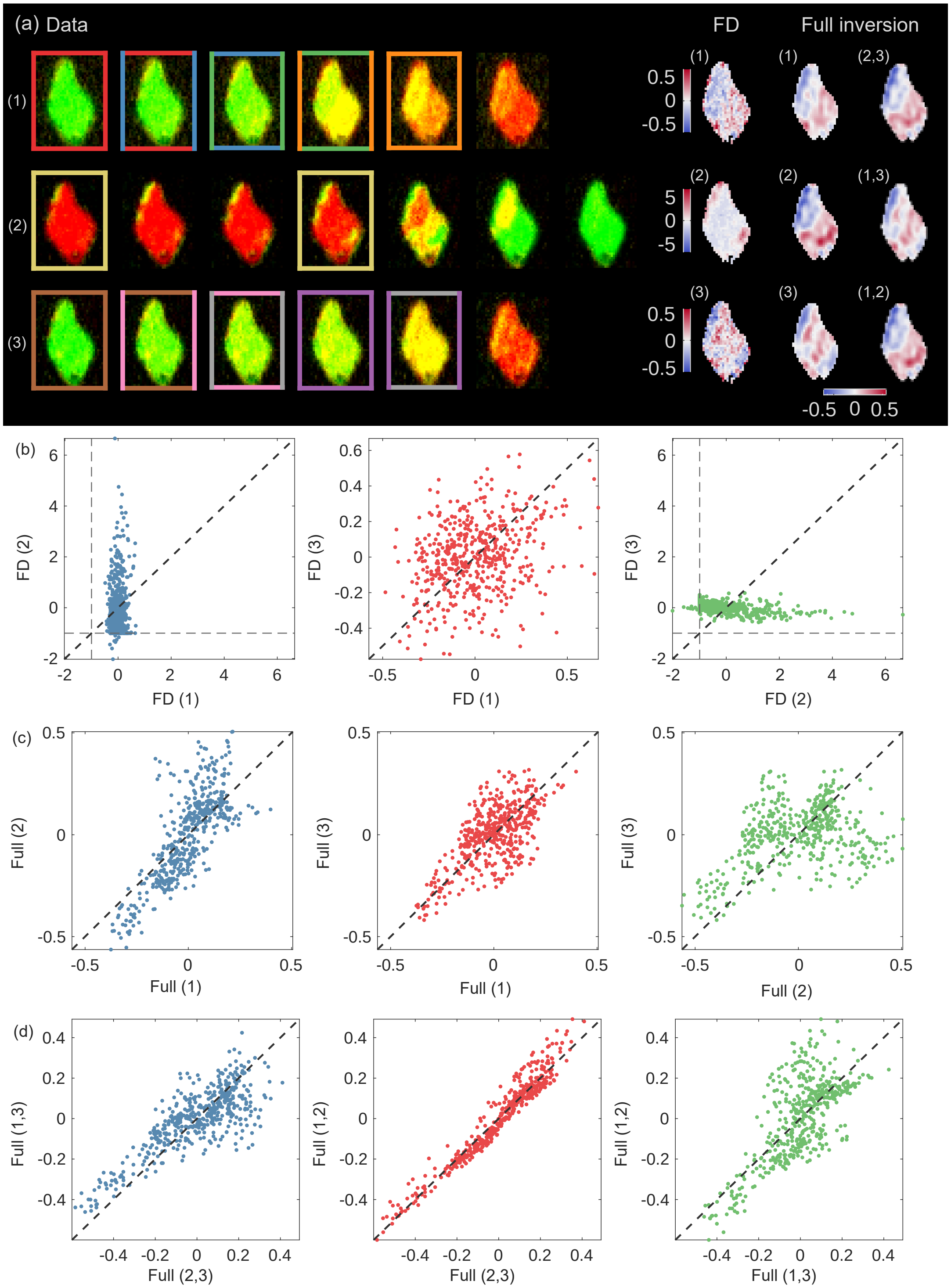

Figure S47: (a) Frames of all three episodes and estimated spatial heterogeneity $\psi(\mathbf{x}) - \bar{\psi}$. Frames chosen for FD estimate of $\ln k(\mathbf{x})$ are highlighted. The same pair is highlighted with the same color. On the right are the FD estimate for each episode and the inversion result at $\rho_2 = 0.88$. The label indicates which dataset is used for training. For example, (1,2) means training on episodes 1 and 2. (b) scatter plots of each pair of the FD estimates. (c,d) Scatter plots of each pair of the full inversion result. Diagonal dashed lines are $y = x$.

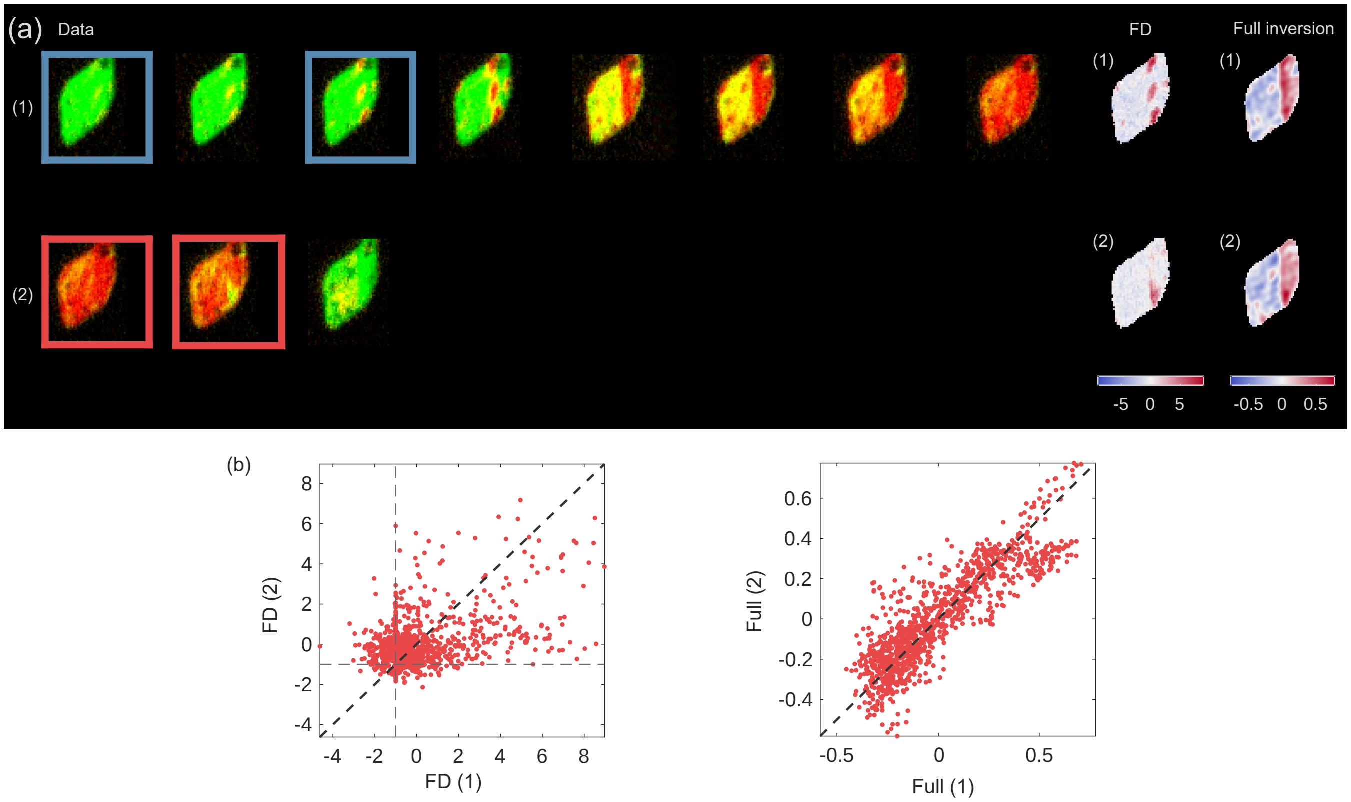

Figure S48: (a) All the frames and estimated spatial heterogeneity of a particle with two episodes. Frames chosen for FD estimate of $\ln k(\mathbf{x})$ are highlighted. The same pair is highlighted with the same color. On the right are the FD estimate for each episode and the inversion result at $\rho_2 = 0.88$. The first and second rows are trained on episodes 1 and 2, respectively. (b) Scatter plots of each pair of FD and full inversion estimates. Diagonal dashed lines are $y = x$.

Table S3: List of optimal RMSE and RMSEV using different models and objective functions. $j_0(c)$, $\mu_h(c)$, and $k(\mathbf{x})$ may either be specified or optimized based on the chosen objective function. lit. refers to the model in the literature (Section 2.3).

Model and objective functions	RMSE	RMSEV
(a) $j_0(c) = \sqrt{c(1-c)}$, $\mu_h(c)$ = lit., $k(\mathbf{x}) = 1$	11.2%	0.0177
(b) $j_0(c)$ and $\mu_h(c)$ are MAP (sec. 8.5), $k(\mathbf{x}) = 1$	11.0%	0.0143
(c) $\min_{j_0(c),\mu_h(c)}$ RMSE, $k(\mathbf{x}) = 1$	10.6%	0.0167
(d) $\min_{j_0(c),\mu_h(c)}$ RMSEV, $k(\mathbf{x}) = 1$	12.8%	0.0104
(e) $j_0(c) = \sqrt{c(1-c)}$, $\mu_h(c)$ = lit., $\min_{k(\mathbf{x})} S(\mathbf{p})$ with $\rho_2 = 0.01$	7.2%	0.0118
(f) $\min_{j_0(c),\mu_h(c),k(\mathbf{x})} S(\mathbf{p})$ with $\rho_2 = 0.01$	6.0%	0.0077
(g) $\min_{j_0(c),\mu_h(c),k(\mathbf{x})} S(\mathbf{p})$ with $\rho_2 = 0.88$	6.8%	0.0095

the secondary phase forms at 650 °C or above, while surface phase change is not observed at 600 °C. Because the carbon coating process in our study is conducted at 600 °C as mentioned in sec. 1.1, the formation of the secondary phase is a less likely explanation for the surface heterogeneity observed in our study compared to the variation of the carbon coating thickness itself.

9.4 Comparison of different models

Table S3 and Fig. S49 compare the RMSE using different models. We introduce another objective function which is the difference in the variance of the concentration field $v(t) = \int (c(x,t) - \bar{c}(t))^2 dx / \int dx$ between model and data. Define square error of variance by

$$\text{SEV} = \sum_i \sum_j \|v_{i,j}(\mathbf{p}_{\text{global}}, \mathbf{Z}_i) - v_{\text{data},i,j}\|^2, \tag{S140}$$

where $v_{i,j}$ and $v_{\text{data},i,j}$ are the model prediction and experimental variance of the concentration field of the jth episode of particle i and the squared L_2 norm is the sum of the squared error over all frames of the episode. Similar to RMSE, define root mean squared error of the variance,

$$\text{RMSEV} = \frac{\text{SEV}}{\sum_i \sum_j N_{p,i}(N_{T,i,j} - 1)}. \tag{S141}$$

The largest reduction in the RMSE (compare (a) and (f) or (g)) is due to the introduction of spatial heterogeneity, i.e., the optimization of $\mu_h(c)$, $j_0(c)$, and $j_0(x)$ simultaneously. We show that when there is no spatial heterogeneity, using the optimal $\mu_h(c)$ and $j_0(c)$ from the full optimization captures the trend in uniformity better than the symmetric model, and results in a lower error in the variance of the concentration field (RMSEV) even though the RMSE does not differ greatly (compare (a) and (b)). We demonstrate this visually with a few examples in Fig. S50, comparing cases (a), (b), and (g). Notice that the baseline (a) results in most uniform concentration fields, hence both RMSE and RMSEV are large; with the optimal $\mu_h(c)$ and $j_0(c)$ (b), the variance of the concentration v is much closer to the data, but details of the simulated patterns may differ from data. By including spatial heterogeneity, both RMSV and RMSEV decrease further and a closer pixel-to-pixel agreement between model and data is achieved.

Without spatial heterogeneity, the optimization of SE can only decrease the RMSE slightly (compare (a) and (c)). This case is equivalent to setting the regularization coefficient for $\ln k(\mathbf{x})$, or ρ_2, to infinity.

Alternatively, when we set the objective function to be RMSEV, or the error in the variance of the concentration field, which measures the heterogeneity of the concentration field and is known to largely be influenced by the form of $j_0(c)$ and $\mu_h(c)$, the resulting RMSEV is significantly reduced compared to using the pixel-wise error in the objective function (compare (c) and (d)).

Comparing the results when spatial heterogeneity is included in the optimization, we find that using the optimal $\mu_h(c)$ and $j_0(c)$ (all three optimized simultaneously) results in a smaller RMSE than when $\mu_h(c)$ and $j_0(c)$ are

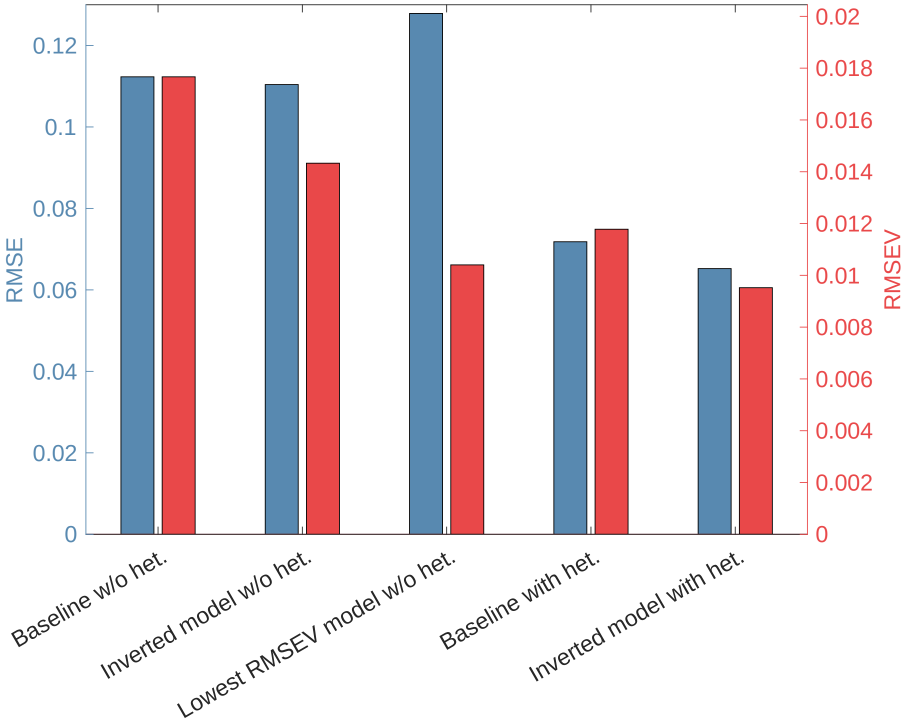

Figure S49: Comparison of the RMSE and RMSEV of some methods in table. S3.

fixed at the baseline and $k(\mathbf{x})$ alone is optimized (compare (e) and (f) or (g)). More notably, the former approach leads to a larger percentage decrease in RMSEV than RMSE, again reflecting the importance of $\mu_h(c)$ and $j_0(c)$ in determining the uniformity of the concentration field.

9.5 Reaction kinetics

In this section, we briefly describe the coupled-ion-electron transfer (CIET) theory [29, 30]. In the case of electron transfer from the metallic carbon coating to the $Fe^{3+/2+}$ redox site [28] coupled with Li^+ ion transfer from the coating into the crystal lattice, excluding one site [7], CIET predicts the Li intercalation rate to be

$$R = j_r^*(1-c)\int_{-\infty}^{\infty}[\tilde{c}_+ n_e(\epsilon)p_{\text{red}}(\epsilon) - c(1-n_e(\epsilon)p_{\text{ox}}(\epsilon)]\rho(\epsilon)d\epsilon \tag{S142}$$

where j_r^* is a constant that depends on the ion transfer energy, electronic coupling, and temperature [29], $\tilde{c}_+$ is the fractional coverage of reactive surface sites by absorbed Li^+, $n_e(\epsilon) = 1/(1+e^{\epsilon/k_BT})$ is the Fermi distribution, ϵ is the electron energy level, $p_{\text{red}}(\epsilon)$ and $p_{\text{ox}}(\epsilon)$ are the normalized electron transfer probabilities of reduction and oxidation, respectively and

$$p_{\text{red}} = \frac{1}{\sqrt{4\pi\lambda k_BT}}\exp\left[-\frac{\lambda + e\eta_f - \epsilon}{4\lambda k_BT}\right] \tag{S143}$$

and

$$p_{\text{ox}} = \frac{1}{\sqrt{4\pi\lambda k_BT}}\exp\left[-\frac{\lambda - e\eta_f + \epsilon}{4\lambda k_BT}\right] \tag{S144}$$

where λ is the reorganization energy of the solid host, which is set to $\lambda = 8.3k_BT$ based on previous estimate[29], η_f is the formal overpotential, defined to be

$$\eta_f = \frac{k_BT}{e}\left(\eta + \ln\frac{\tilde{c}_+}{c}\right) + RR_c \tag{S145}$$

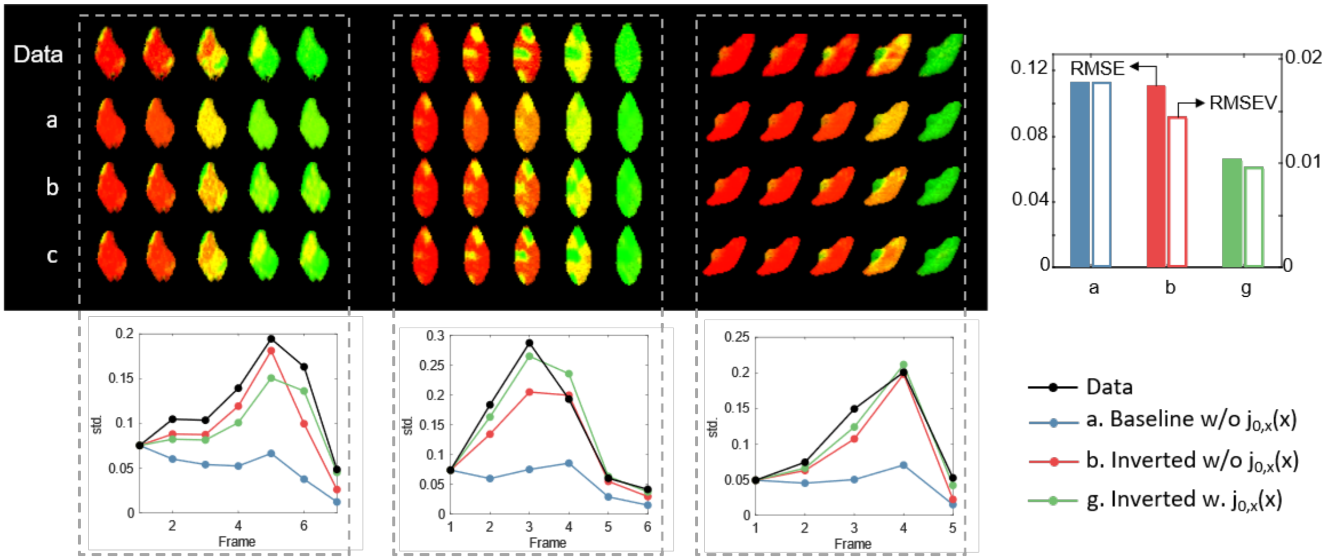

Figure S50: Comparison of experimental (data) and simulated Li concentration field based on three methods (a,b,g) in Table S3 for key frames of three selected episodes. The methods chosen are (based on the numbering in Table S3) (a) baseline model without spatial heterogeneity, (b) $j_0(c)$ and $g_h(c)$ from MAP without spatial heterogeneity, and (g) inverted model with spatial heterogeneity included. The standard deviation of the concentration field (std $= v^{1/2}$) of all frames (from data and models) are shown below each episode. The RMSE and RMSEV of these three models quantified by all episodes (including those not shown) are shown in the bar graph.

where R_c is the ionic resistance of the carbon coating on the surface. R_c can be related to the surface heterogeneity j_s. Here, we focus only on the non-spatially dependent reaction kinetics and hence set $R_c = 0$. Assuming surface adsorption of Li^+ is fast compared to CIET, the surface coverage can be related to the bulk via Langmuir isotherm for non-interacting surface sites

$$\tilde{c}_+ = \frac{a_+ e^{-w_+/k_B T}}{1 + a_+ e^{-w_+/k_B T}} \tag{S146}$$

where a_+ is the activity of Li^+ in the electrolyte and w_+ is the Li^+ surface adsorption energy.

Ref. [35] derived a simple formula that accurately approximates the full expression in Eq. S142,

$$R = j_r^* (1 - c) \left(\frac{\tilde{c}_+}{1 + e^{\tilde{\eta}_f}} - \frac{c}{1 + e^{-\tilde{\eta}_f}} \right) \operatorname{erfc} \left(\frac{\tilde{\lambda} - \sqrt{1 + \sqrt{\tilde{\lambda}} + \tilde{\eta}_f^2}}{2\sqrt{\tilde{\lambda}}} \right) \tag{S147}$$

where for simplicity of notation, we define the normalized reorganization energy $\tilde{\lambda} = \lambda/(k_B T)$, and normalized formal overpotential $\tilde{\eta}_f = \eta_f e/(k_B T)$. From Eq. S147, we can obtain the exchange current predicted by the CIET theory when at low overpotential ($\eta \to 0$) shown in Eq. 3 in the main text. At low overpotential, $R = -j_{0,\mathrm{CIET}}(c)\tilde{\eta}$, which can be directly compared with Butler-Volmer (BV) kinetics at low overpotential because from Eq. S8, we have $R \to -j_0(c)\tilde{\eta}$. Fig. 3a in the main text compares $j_{0,\mathrm{CIET}}(c)$ and $j_0(c)$ from the inversion.

Next, we would also like to compare CIET with the inferred reaction kinetics based on BV at higher overpotential. Because the dependence of CIET reaction rate on c and η is non-separable, in order to compare with Butler-Volmer (BV) kinetics, we need to first quantify the distribution of η.

Fig. S51 shows the distribution of overpotential at all frames and pixel locations when trained using all datasets and at the optimal regularization coefficient $\rho_2 = 0.88$, as well as the distribution for each episode. The standard deviation is $2.7 k_B T/e$, which is consistent with the estimate of the overpotential in Ref. [1] ($2.5 k_B T/e$) which is used to compare an experimental estimate of reaction rate with CIET prediction in Ref. [29].

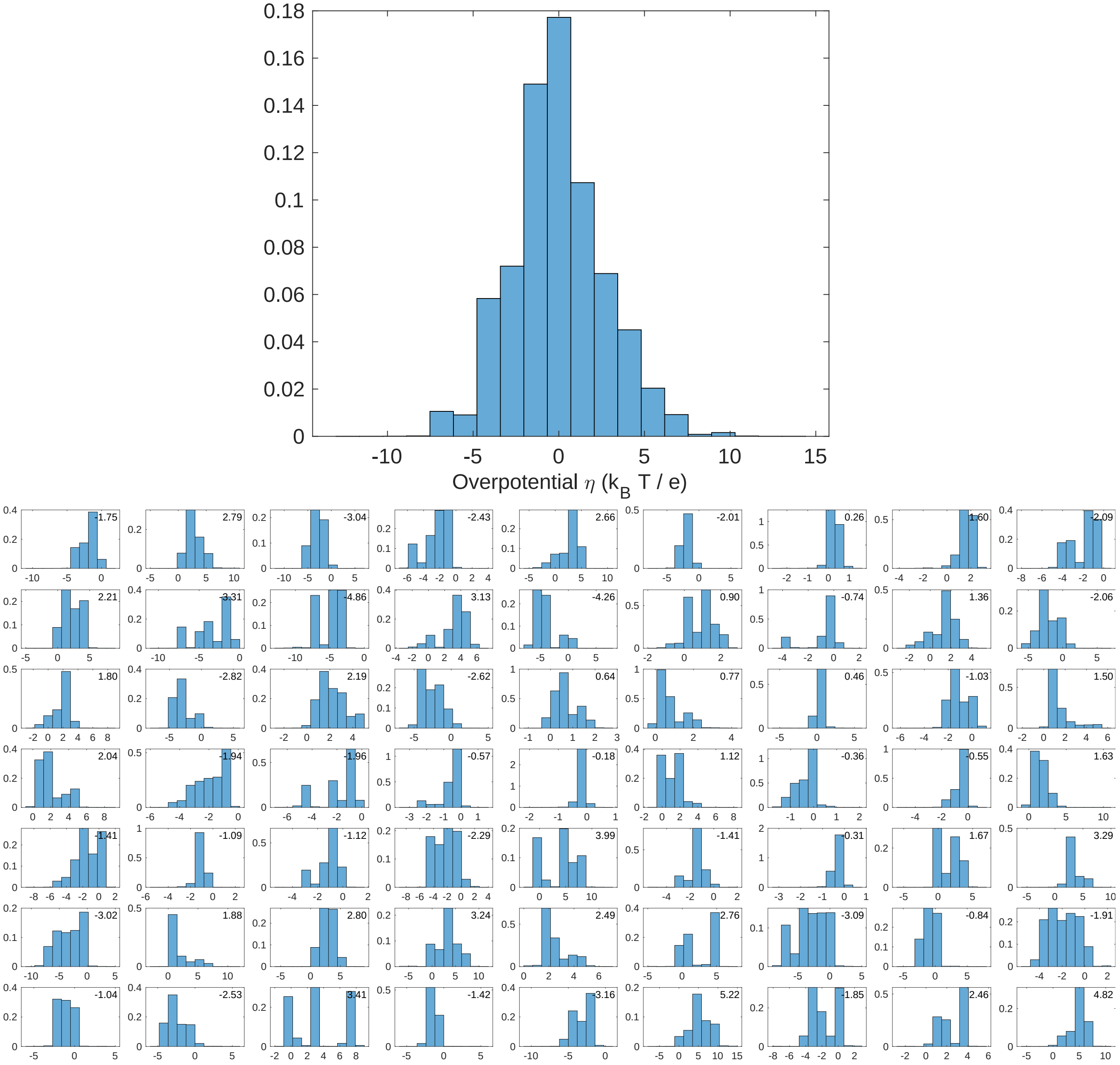

Figure S51: The histogram of overpotential (a) at all frames and pixel locations (b) for each episode when trained using all datasets and at the optimal regularization coefficient $\rho_2 = 0.88$. The label is the mean overpotential of the episode.

We first compare CIET and BV reaction rates at various overpotential by assuming the surface coverage $\tilde{c}_+ = 1$ (which corresponds to a strong affinity of Li^+ to the surface).

Fig. S52 compares $R(c, \eta)$ between CIET and BV using inverted $j_0(c)$ as well as $|R(c, \eta)|/\max_c |R(c, \eta)|$, because we are more interested in the normalized reaction rate since the magnitude of $j_0(c)$ cannot be accurately inferred. For BV, $|R(c, \eta)|/|\max_c R(c, \eta)| = j_0(c)/j_{0,\max}$. We find that the normalized reaction rate predicted by CIET in the range of $[-5, 5]k_BT/e$ is mostly within the 99% confidence interval of the normalized reaction rate inferred from the images.

Next, we study the effect of $\tilde{c}_+$. The electrolyte used in this study is 1M $LiClO_4$ in tetraethylene glycol dimethyl ether. $\tilde{c}_+$ cannot be directly measured. As a reference, we use the activity of 0.358 mol/kg $LiClO_4$ in dimethoxyethane $a_+ = 0.015$ [77] as a reference and vary w_+/k_BT from -10 to 10. Fig. S53 shows the normalized rate $|R(c, \eta)|/\max_c |R(c, \eta)|$ at $\eta = -5, 0, 5k_BT/e$, $a_+ = 0.015$ and at various w_+. As w_+ decreases (increasing affinity), the peak of the normalized rate shifts to higher values of c, asymptotically approaching the limit when $\tilde{c}_+ = 1$.

Finally, we compare the inverted reaction rate with an extension of the CIET theory to the case where the rate is limited by ion transfer (ion-coupled electron transfer, ICET), which predicts the same overpotential dependence as BV up to large overpotential with a new functional form for the exchange current $j_{0,\mathrm{ICET}}(c) = c^\alpha(1 - c)$ [34]. Fig. S54 shows the comparison of inverted and its best fit to $j_{0,\mathrm{ICET}}(c)$ with $\alpha = 0.66$. The inverted concentration dependence is quantitatively consistent with the unified quantum theory of CIET for intercalation, whether rate is limited by ion transfer (ICET) or by electron transfer (electron-coupled ion transfer, ECIT)[34]. In contrast, the prevailing empirical BV model, $j_0(c) = \sqrt{c(1 - c)}$[39], lies well outside the error bounds and cannot fit the experimental data.

9.6 Variation in concentration field

Fig. S55 shows the uniformity coefficient versus I/I_0, where the uniformity coefficient is defined as (see also 5.1)

$$\mathrm{UC} = 1 - \frac{\sum_i \left(v(t_i)\bar{c}(t_i)(1 - \bar{c}(t_i))\right)^{1/2}}{\sum_i \bar{c}(t_i)(1 - \bar{c}(t_i))} \tag{S148}$$

where the summation is taken over all frames of each episode, $\bar{c}(t_i)$ is the spatially average composition at time t_i, and again, the variance of the concentration field is

$$v = \frac{\int (c(x) - \bar{c})^2 dx}{\int dx} \tag{S149}$$

where the integral here is performed by summing over all pixels that belong to the particle. The uniformity coefficient UC fits the standard deviation (std) of the concentration field to std = $(1 - \mathrm{UC})\sqrt{\bar{c}(1 - \bar{c})}$. When UC is 1, the concentration field is uniform; when UC is 0, the std is maximum, i.e., consisting of 0 and 1. I is defined to be the average reaction rate, that is, $I = (\bar{c}(t_n) - \bar{c}(t_1))/(t_n - t_1)$, where t_1 and t_n are the time of the first and last frames, respectively. $I_0 = \int k(\mathbf{x})dx / \int dx$. We find that, while there is a significant spread due to the different initial conditions, geometry, spatial heterogeneity, and the non-constant total reaction rate $I(t)$, the general trend is that the concentration field $c(x)$becomes more uniform with increasing reaction rate and that $c(x)$ is more uniform during Li insertion than extraction.

10 Inversion result

Fig. S56 compares the standard deviation ($v^{1/2}$) of $c(x)$ of all frames between data and model. We find good agreement between the two.

Fig. S57 displays all the experimental images and the inversion result using the optimal regularization parameter $\rho_2 = 0.88$ determined in Section 8.6 trained on the entire dataset. The inversion results include $k(\mathbf{x})$ of each particle and the concentration field $c(x, t)$ predicted by the inferred model.

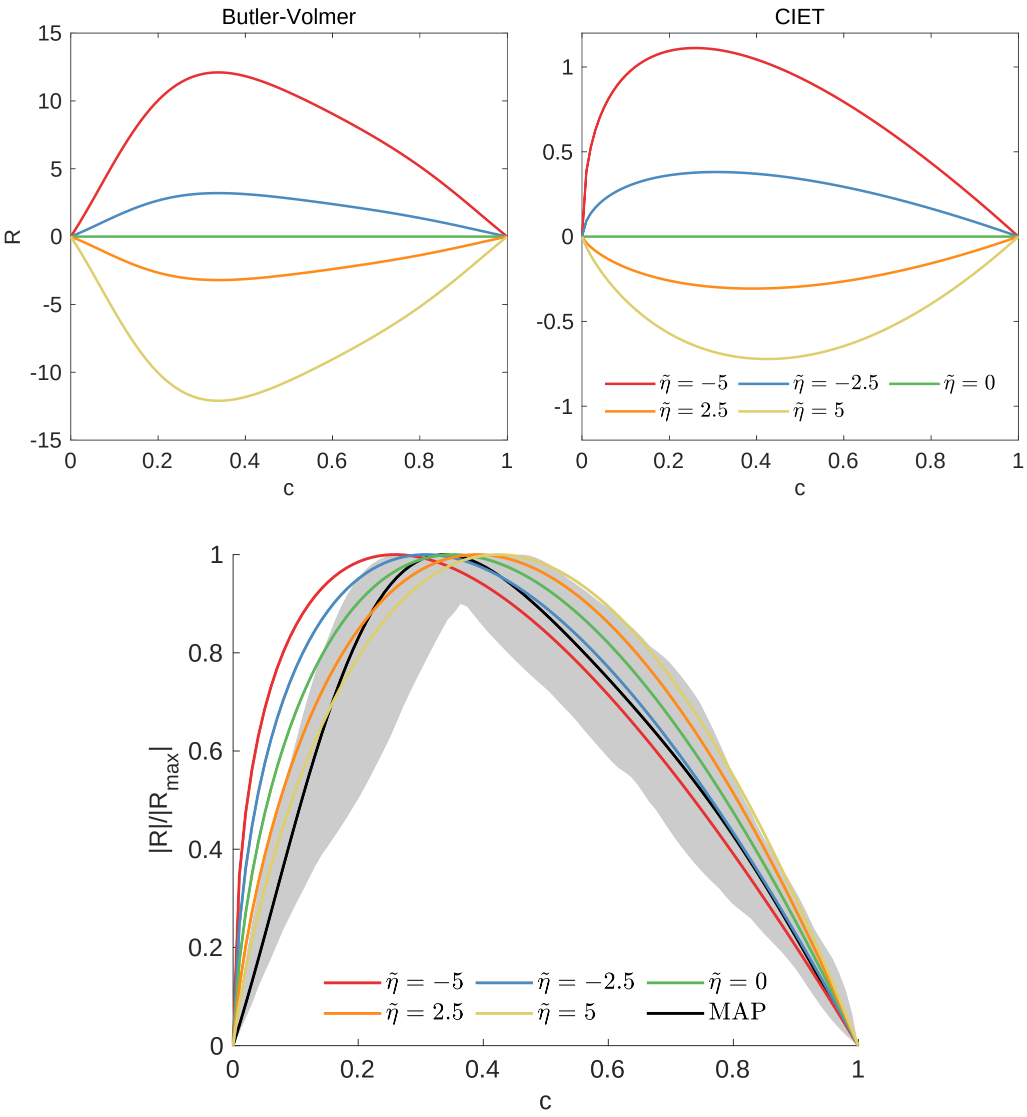

Figure S52: Comparison of $R(c, \eta)$ and $|R(c, \eta)|/|\max_c R(c, \eta)|$ between CIET ($\tilde{c}_+ = 1$) at different overpotential and the inferred reaction kinetics based on Butler-Volmer kinetics. For the latter, $R(c, \eta) = 2j_0(c)\sinh\tilde{\eta}/2$ where $j_0(c)$ is from the MAP result, and $|R(c, \eta)|/|\max_c R(c, \eta)| = j_0(c)/j_{0,\max}$ is shown with the shaded 99% confidence interval as well as the MAP result in the black curve (same as Fig. 3a in the main text).

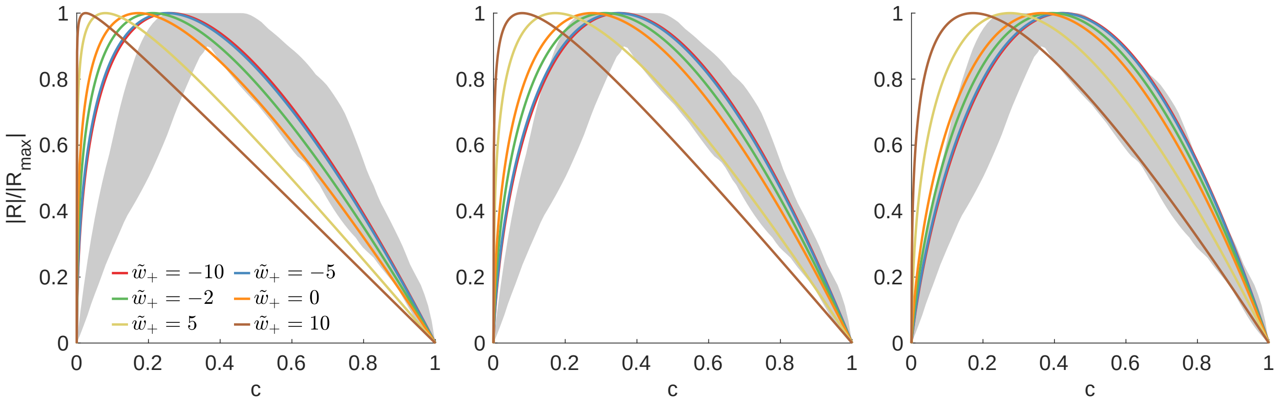

Figure S53: Comparison of $|R(c,\eta)|/|\max_c R(c,\eta)|$ predicted by CIET at $\eta = 2.5k_BT/e$, $a_+ = 0.015$ and various values of surface adsorption energy $\tilde{w}_+ = w_+/k_BT$ with the inferred reaction kinetics based on Butler-Volmer kinetics.

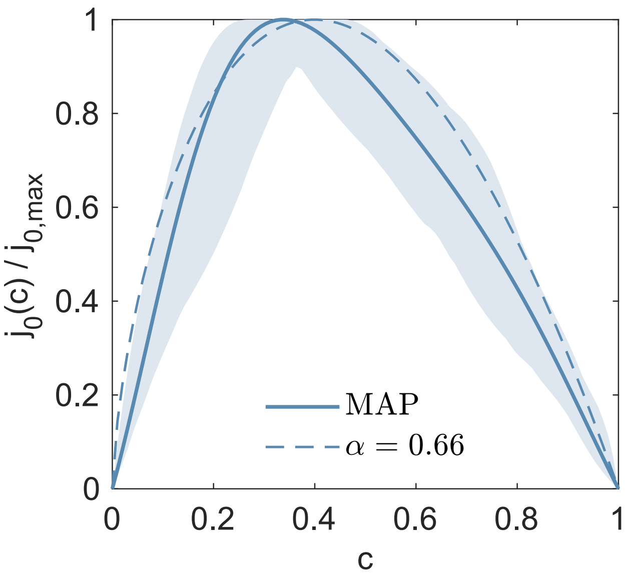

Figure S54: Comparison between the inferred exchange current $j_0(c)$ (MAP in solid curve and 99% confidence region in the shaded region) and the best fit $j_{0,\mathrm{ICET}}(c) = c^{\alpha}(1-c)$ which is based on an extension of the CIET theory to ion transfer limitation.

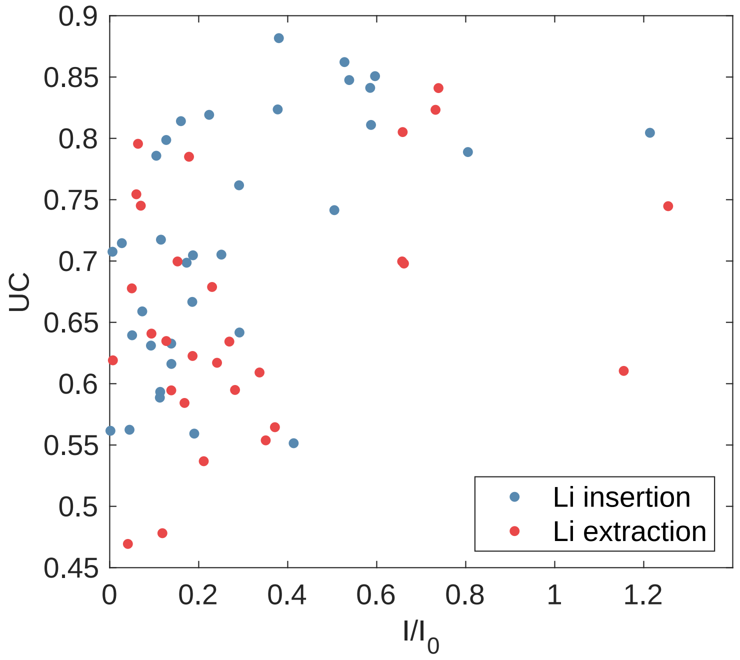

Figure S55: The uniformity coefficient of each episode computed from image data versus I/I_0.

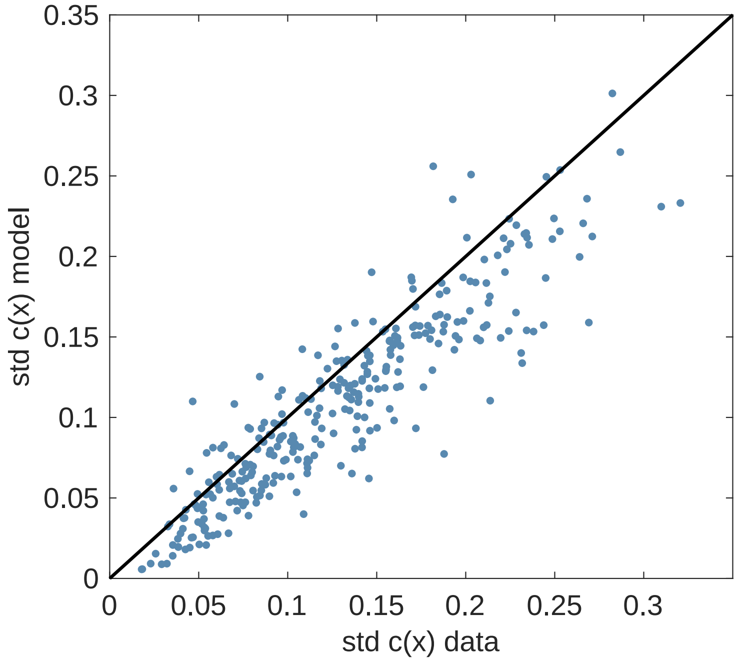

Figure S56: Standard deviation (std = $v^{1/2}$) of $c(x)$ of all frames between data and learned model.

SI Video 1 shows the same comparison of all experimental images and the inversion result as in Fig. S57 interpolated in time using pchip. The scale bar is 1 mm. The arms with green and red-dotted ends indicate hours and minutes since the first frame, or the beginning of the movie, respectively.

Supplementary Information References

[55] Qin, X. *et al.* Hydrothermally synthesized lifepo 4 crystals with enhanced electrochemical properties: Simultaneous suppression of crystal growth along [010] and antisite defect formation. *Physical Chemistry Chemical Physics* **14**, 2669–2677 (2012).

[56] Chen, J. & Graetz, J. Study of antisite defects in hydrothermally prepared lifepo4 by in situ x-ray diffraction. *ACS Applied Materials and Interfaces* **3**, 1380–1384 (2011).

[57] Shapiro, D. A. *et al.* Chemical composition mapping with nanometre resolution by soft x-ray microscopy. *Nature Photonics* **8**, 765–769 (2014).

[58] May, B. M. *et al.* Nanoscale detection of intermediate solid solutions in equilibrated lixfepo4 microcrystals. *Nano Letters* **17**, 7364–7371 (2017).

[59] Chueh, W. C. *et al.* Intercalation pathway in many-particle $LiFePO_4$ electrode revealed by nanoscale state-of-charge mapping. *Nano Letters* **13**, 866–872 (2013).

[60] Li, Y. *et al.* Current-induced transition from particle-by-particle to concurrent intercalation in phase-separating battery electrodes. *Nature Materials* **13**, 1149–1156 (2014). .

[61] Hedberg, C. L. *Handbook of Auger electron spectroscopy: a book of reference data for identification and interpretation in Auger electron spectroscopy* (Physical Electronics Incorporated, 1995).

[62] Carlson, T. A. *Photoelectron and Auger Spectroscopy* (Springer US, 1975).

[63] Li, Y. *et al.* Dichotomy in the lithiation pathway of ellipsoidal and platelet $LiFePO_4$ particles revealed through nanoscale operando state-of-charge imaging. *Advanced Functional Materials* **25**, 3677–3687 (2015).

[64] Malik, R., Burch, D., Bazant, M. & Ceder, G. Particle size dependence of the ionic diffusivity. *Nano Letters* **10**, 4123–4127 (2010). .

[65] Newman, J. & Thomas-Alyea, K. E. *Electrochemical Systems* (John Wiley & Sons, Hoboken, New Jersey, 2012).

[66] Ramana, C. V., Mauger, A., Gendron, F., Julien, C. M. & Zaghib, K. Study of the li-insertion/extraction process in lifepo4 / fepo4. *J. Power Sources* **187**, 555–564 (2009).

[67] Smith, K. C., Mukherjee, P. P. & Fisher, T. S. Columnar order in jammed lifepo4 cathodes: ion transport catastrophe and its mitigation. *Physical Chemistry Chemical Physics* **14**, 7040 (2012). .

[68] Ohmer, N. *et al.* Phase evolution in single-crystalline lifepo4 followed by in situ scanning x-ray microscopy of a micrometre-sized battery. *Nature Communications* **6** (2015).

[69] Zhu, Y. *et al.* In situ atomic-scale imaging of phase boundary migration in fepo 4 microparticles during electrochemical lithiation. *Advanced Materials* **25**, 5461–5466 (2013).

[70] Laffont, L. *et al.* Study of the $LiFePO_4/FePO_4$ two-phase system by high-resolution electron energy loss spectroscopy. *Chemistry of Materials* **18**, 5520–5529 (2006). .

[71] Kobayashi, S., Kuwabara, A., Fisher, C. A. J., Ukyo, Y. & Ikuhara, Y. Microscopic mechanism of biphasic interface relaxation in lithium iron phosphate after delithiation. *Nature Communications* **9**, 2863 (2018). .

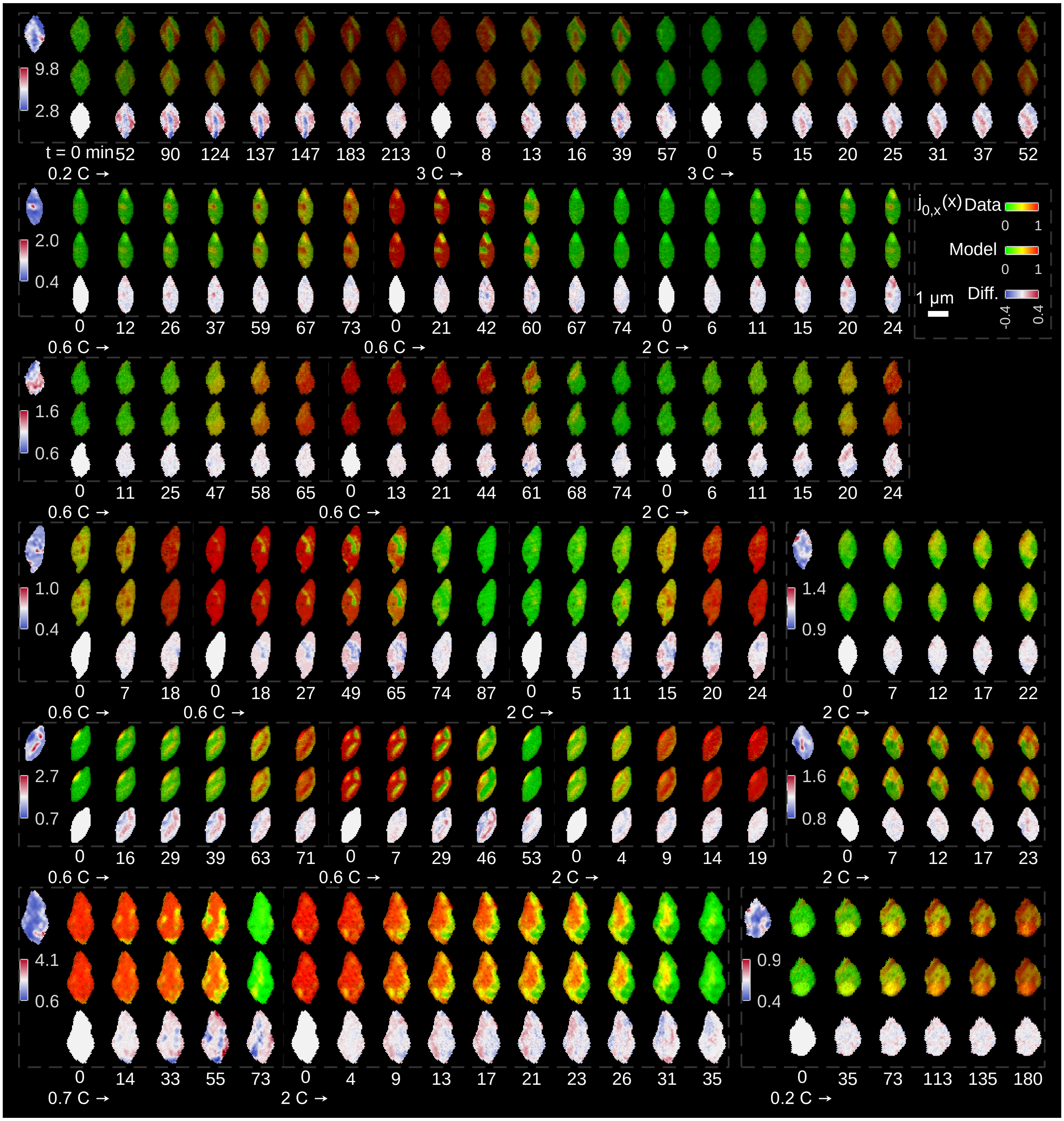
9.8
2.8
t = 0 min 52 90 124 137 147 183 213 0 8 13 16 39 57 0 5 15 20 25 31 37 52
0.2 C →
3 C →
3 C →
j0,x(x) Data
0 1
Model
0 1
1 μm
Diff.
-0.4
0.4
2.0
0.4
0 12 26 37 59 67 73 0 21 42 60 67 74 0 6 11 15 20 24
0.6 C →
0.6 C →
2 C →
1.6
0.6
0 11 25 47 58 65 0 13 21 44 61 68 74 0 6 11 15 20 24
0.6 C →
0.6 C →
2 C →
1.0
0.4
0 7 18 0 18 27 49 65 74 87 0 5 11 15 20 24
0.6 C →
0.6 C →
2 C →
1.4
0.9
0 7 12 17 22
2 C →
2.7
0.7
0 16 29 39 63 71 0 7 29 46 53 0 4 9 14 19
0.6 C →
0.6 C →
2 C →
1.6
0.8
0 7 12 17 23
2 C →
4.1
0.6
0 14 33 55 73 0 4 9 13 17 21 23 26 31 35
0.7 C →
2 C →
0.9
0.4
0 35 73 113 135 180
0.2 C →

Figure S57

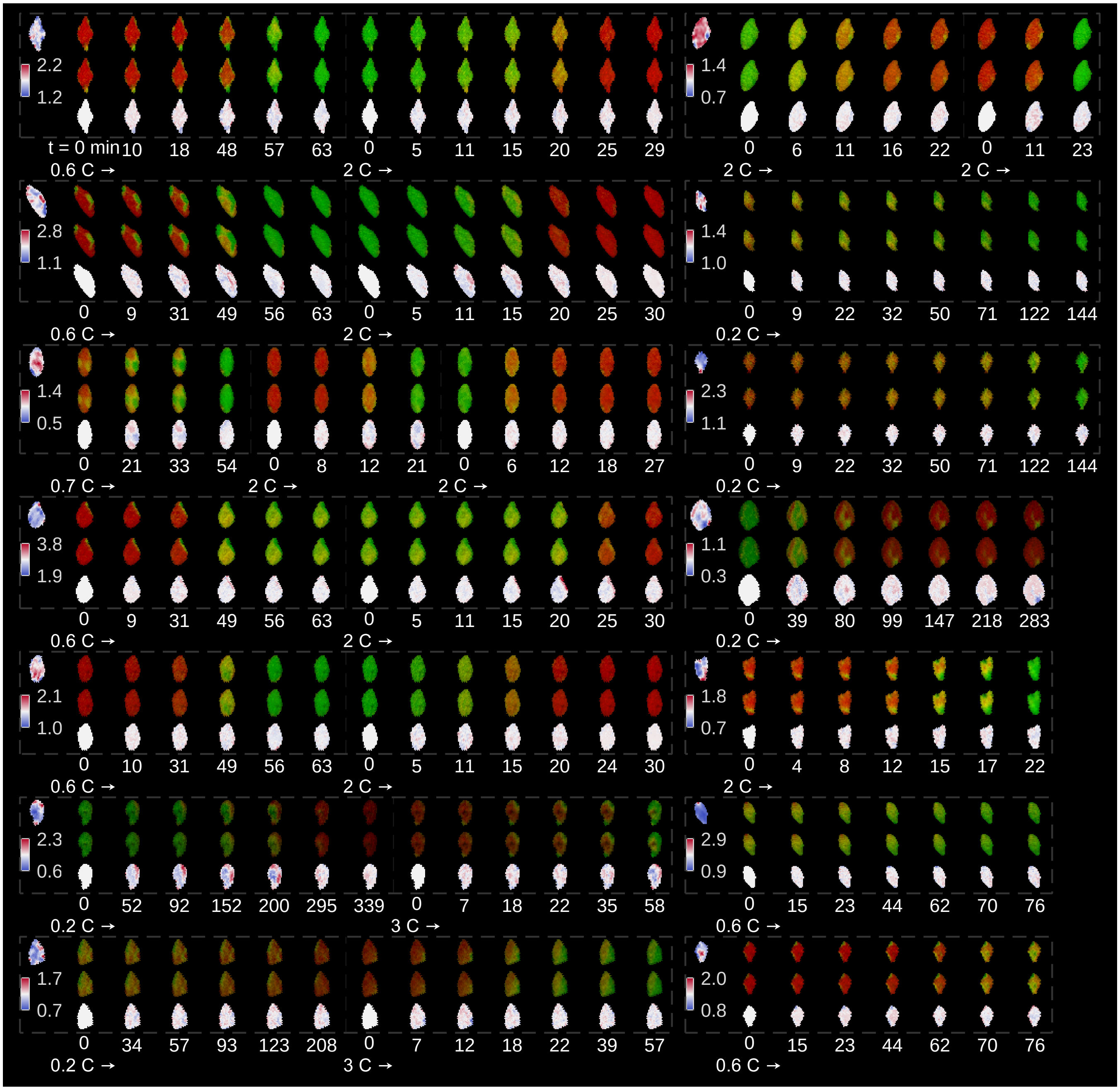
2.2
1.2
t = 0 min 10 18 48 57 63 0 5 11 15 20 25 29
0.6 C →
2 C →
1.4
0.7
0 6 11 16 22 0 11 23
2 C →
2 C →
2.8
1.1
0 9 31 49 56 63 0 5 11 15 20 25 30
0.6 C →
2 C →
1.4
1.0
0 9 22 32 50 71 122 144
0.2 C →
1.4
0.5
0 21 33 54 0 8 12 21 0 6 12 18 27
0.7 C →
2 C →
2 C →
2.3
1.1
0 9 22 32 50 71 122 144
0.2 C →
3.8
1.9
0 9 31 49 56 63 0 5 11 15 20 25 30
0.6 C →
2 C →
1.1
0.3
0 39 80 99 147 218 283
0.2 C →
2.1
1.0
0 10 31 49 56 63 0 5 11 15 20 24 30
0.6 C →
2 C →
1.8
0.7
0 4 8 12 15 17 22
2 C →
2.3
0.6
0 52 92 152 200 295 339 0 7 18 22 35 58
0.2 C →
3 C →
2.9
0.9
0 15 23 44 62 70 76
0.6 C →
1.7
0.7
0 34 57 93 123 208 0 7 12 18 22 39 57
0.2 C →
3 C →
2.0
0.8
0 15 23 44 62 70 76
0.6 C →

Figure S57

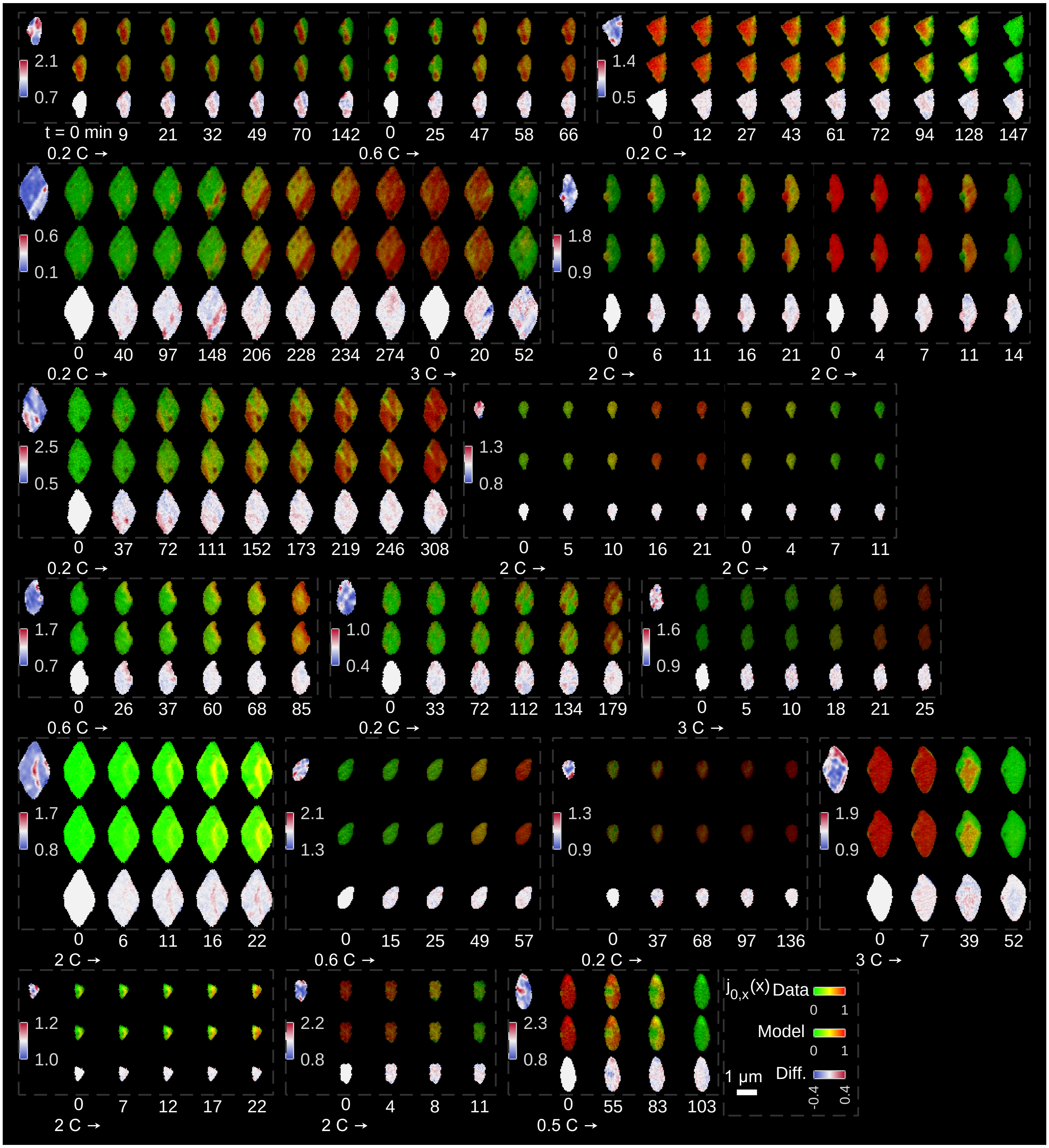

Figure S57: All the experimental images and the inversion result using the optimal regularization parameter $\rho_2 = 0.88$. The inferred $k(\mathbf{x})$ and the corresponding colormap of each particle is displayed to the left of the concentration fields. For each particle, the first row is the experimental data, the second row is the model, and the third row is the difference between data and model. Colorbars for them and the scale bar are shared and shown on the right. The time duration from the first frame and the global C-rate of each episode is shown below the images.

[72] Torchio, M., Magni, L., Gopaluni, R. B., Braatz, R. D. & Raimondo, D. M. LIONSIMBA: A Matlab framework based on a finite volume model suitable for Li-ion battery design, simulation, and control. *Journal of The Electrochemical Society* **163**, A1192–A1205 (2016).

[73] Shampine, L. & Reichelt, M. Ode matlab solvers. *Journal of Scientific Computing* **18**, 1–22 (1997).

[74] McLean, R. A., Sanders, W. L. & Stroup, W. W. A unified approach to mixed linear models. *American Statistician* **45**, 54–64 (1991).

[75] Wang, J. & Sun, X. Understanding and recent development of carbon coating on $LiFePO_4$ cathode materials for lithium-ion batteries. *Energy Environ. Sci.* **5**, 5163–5185 (2012).

[76] Wang, J. *et al.* Size-dependent surface phase change of lithium iron phosphate during carbon coating. *Nature Communications* **5**, 3415 (2014). .

[77] Barthel, J., Neueder, R., Poepke, H. & Wittmann, H. Osmotic coefficients and activity coefficients of nonaqueous electrolyte solutions. part 2. lithium perchlorate in the aprotic solvents acetone, acetonitrile, dimethoxyethane, and dimethylcarbonate. *Journal of Solution Chemistry* **28** (1999).
